# SuperSILAC Quantitative Proteome Profiling of Murine Middle Ear Epithelial Cell Remodeling with NTHi

**DOI:** 10.1371/journal.pone.0148612

**Published:** 2016-02-09

**Authors:** Stéphanie Val, Katelyn Burgett, Kristy J. Brown, Diego Preciado

**Affiliations:** 1 Sheikh Zayed Center for Pediatric Surgical Innovation, Children’s National Health System, Washington, DC, United States of America; 2 Center for Genetic Medicine Research, Children’s National Health System, Washington, DC, United States of America; 3 Division of Pediatric Otolaryngology, Children’s National Health System, Washington, DC, United States of America; Public Health England, UNITED KINGDOM

## Abstract

**Background:**

Chronic Otitis Media with effusion (COME) develops after sustained inflammation and is characterized by secretory middle ear epithelial metaplasia and effusion, most frequently mucoid. Non-typeable *Haemophilus influenzae (NTHi)*, the most common acute Otitis Media (OM) pathogen, is postulated to promote middle ear epithelial remodeling in the progression of OM from acute to chronic. The goals of this study were to examine histopathological and quantitative proteomic epithelial effects of NTHi challenge in a murine middle ear epithelial cell line.

**Methods:**

NTHi lysates were generated and used to stimulate murine epithelial cells (mMEEC) cultured at air-liquid interface over 48 hours– 1 week. Conditional quantitative Stable Isotope Labeling with Amino Acids in Cell Culture (SILAC) of cell lysates was performed to interrogate the global protein production in the cells, using the SuperSILAC technique. Histology of the epithelium over time was done to measure bacterial dependent remodeling.

**Results:**

Mass spectrometry analysis identified 2,565 proteins across samples, of which 74 exhibited differential enrichment or depletion in cell lysates (+/-2.0 fold-change; p value<0.05). The key molecular functions regulated by NTHi lysates exposure were related to cell proliferation, death, migration, adhesion and inflammation. Finally, chronic exposure induced significant epithelial thickening of cells grown at air liquid interface.

**Conclusions:**

NTHi lysates drive pathways responsible of cell remodeling in murine middle ear epithelium which likely contributes to observed epithelial hyperplasia *in vitro*. Further elucidation of these mediators will be critical in understanding the progression of OM from acute to chronic at the molecular level.

## Introduction

Otitis Media (OM) is one of the most common conditions of early childhood accounting for a very high proportion of all pediatric physician office visits annually [1] at a national health care cost estimated to be greater than $1 billion[2,3]. In the first years of life, a majority of children at some point will experience OM[4,5]. Tympanostomy tube placement to treat OM is the most common pediatric surgical procedure requiring anesthesia in the United States[6]. Acute Otitis Media (AOM) is defined as acute onset of middle ear effusion along with signs and symptoms of middle ear inflammation and infection. Chronic Otitis Media (COM) *subsequently* results as a long term sequelae of the acute middle ear infection, and is characterized by secretory epithelial metaplasia and persistence of middle ear effusion, most frequently mucoid[7,8,9].

NTHi is the most common infectious pathogen in AOM[10,11]. Previous work from our group and others has demonstrated that middle ear NTHi challenge in mice leads to chronic epithelial mucosal metaplasia and over-expression of inflammatory mediators[12] [13]. Exact mechanisms of epithelial hyperplasia, however, remain markedly understudied.

We hypothesized that NTHi lysate stimulation results in measureable altered protein levels in mMEEC over time. We postulated that induced mediators are likely to be important in cell-to-cell interactions and epithelial remodeling. Further, we aimed to determine whether this response correlated to middle ear thickening and metaplasia, similar to what we observed *in vivo[13]*. For this purpose, we developed an *in vitro* model of differentiated mouse middle ear epithelial cells cultured at air-liquid interface (ALI) and treated chronically with NTHi lysates in order to mimic chronic otitis media events and characterize and observe the remodeling of the epithelium. We evaluated quantitatively the proteome of mMEEC using the SuperSILAC technique after 1 treatment (48hrs), 2 treatments (96hrs) or 3 treatments (1 week) to NTHi lysates. These studies were completed by the histological observation of cells after chronic treatment.

## Methods

### Bacteria culture and preparation of lysates

NTHi was grown on chocolate agar at 37°C in 5% CO2 overnight and inoculated in brain heart infusion (BHI, BD Laboratories, Franklin Lakes, NY), broth supplemented with 10 mg of nicotinamide adenine dinucleotide per ml (Sigma-Aldrich, Saint Louis, MI). After overnight incubation, bacteria were subcultured into 500 ml of fresh BHI; upon reaching log phase growth, bacteria were centrifuged 10 min at 10,000g at room temperature and suspended in Ham’s F-12 Nutrient Mix supplemented with L-Glutamine (Life Technologies, Carsbad, CA). Sonication was then performed 15 times 15 seconds by 4mL batches in 15mL tubes staying in ice. Then the lysates were centrifuged at 10,000g for 10 min at 4°C to remove the remaining not lysed bacteria as well as debris of cells. Stock solutions of 3 to 8mg/ml were aliquoted by 1mL and stored at -20°C. The usage of lysates was chosen as this is what is released in children after antibiotic treatment for acute otitis media, and is what the vast majority of groups have used to study middle ear and respiratory inflammation effects[14,15]. Indeed we have revently reported on the protein make-up of NTHi lysates[16] and that bacterial lysate preparations are often more pro-inflammatory that live bacteria[17].

### Cell lines and treatments

The mouse middle ear epithelial cell line mMEEC was graciously provided by Dr. Jizhen Lin (University of Minnesota, Minneapolis, MN). These cells are immortalized by a temperature sensitive simian virus 40 (SV40), allowing for a proliferative phenotype at 33°C and for differentiation at 37°C [18]. mMEEC were maintained and passaged in full growth media (FGM) as previously described [19]. Prior to experimentation, cells were transferred to a 37°C, 5% CO2 humidified atmosphere to inactivate the SV-40 virus. For experimentation, cell lines were cultured under differentiating conditions after three weeks of growth on collagen coated transwells at air-liquid interface (ALI) as previously described[19]. Six wells were done for each expermiental and control condition. mMEEC were treated at the apical side of the membrane with 1ml of serum free medium with 200 μg/ml NTHi lysates during only 2 hour maximum to avoid cell death. The cells were recovered after 1 treatment (48hrs), 2 treatments (96hrs) or 3 treatments (1 week). For the histopathological observations, the cells were treated longer times: 1, 2 or 3 weeks with 3 treatments per week.

### SuperSILAC

**Fig 1:**
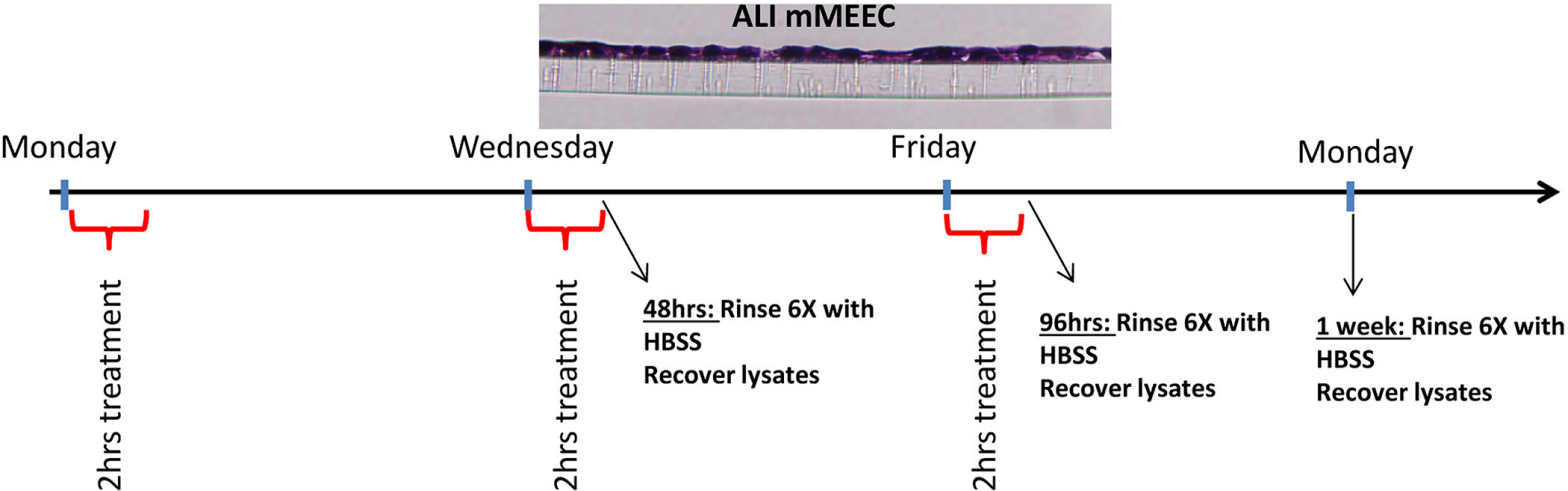
Experimental Design for the SuperSILAC experiments. mMEEC were differentiated at air-liquid interface (ALI) on membranes during 3 weeks. Cells were treated one time on Monday only; Monday and Tuesday; or Monday, Tuesday and Friday for 2 hours in serum free medium with medium alone or supplemented with 200 μg/ml of NTHi lysates at the apical side. Cells were recovered 48 hours (1 treatment), 96 hours (2 treatments) or 1 week (3 treatments) after the first treatment. Cell lysates were recovered after rinsing 6 times with HBSS to remove any trace of serum.

**Fig 2:**
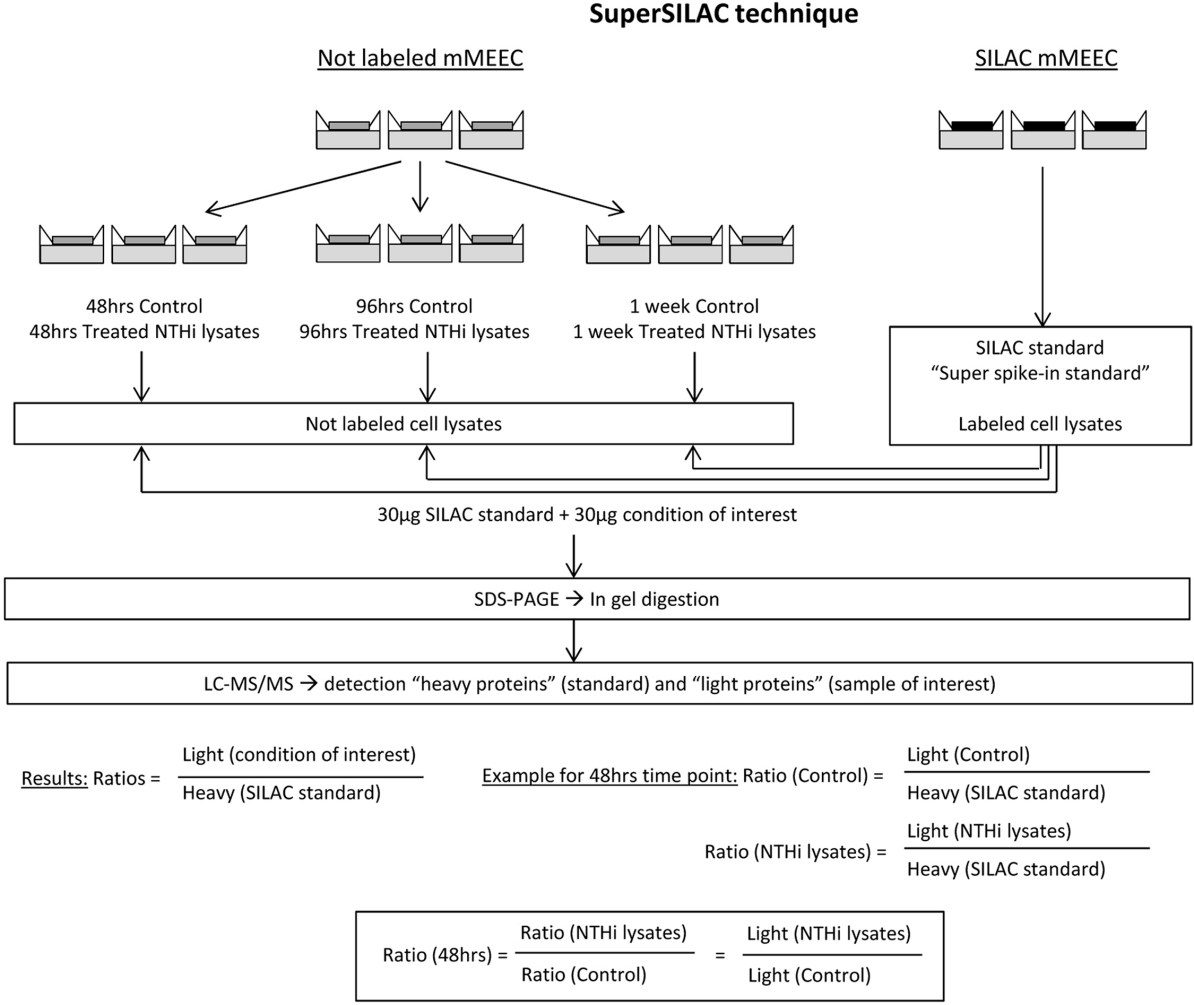
SuperSILAC technique applied to our experiments. Differentiated mMEEC were cultured with labeled arginine and lysine (SILAC mMEEC on the right) or in regular medium (not labeled mMEEC). The lysates of mMEEC with labeled proteins were recovered and called Super spike-in standard, or SILAC standard. Unlabeled mMEEC were treated with NTHi lysates vs. control at the depicted times and the lysates were recovered after 48 hours, 96 hours or 1 week. 30 μg of unlabeled proteins (conditions of interest) were spiked with 30 μg labeled standard, a SDS PAGE was performed followed by in gel digestion to generate peptides for LC-MS/MS detection. The results were expressed as ratios of light proteins (condition of interest) over heavy proteins (standard). With each sample being expressed over the same reference, time point ratios of treated over control were then calculated.

mMEEC cell line was cultured at ALI in Ham’s without arginine and lysine (Thermo Scientific, Belfonte, PA) supplemented with^13^C_6_, ^15^N_2_-Labeled Lysine and ^13^C_6_-labeled Arginine (Cambridge Isotope Laboratories, Andover, MA) with supplements (see Cell culture part) for 5 cell passages until complete incorporation of the labeled amino-acids (verified by LC-MS/MS preliminary experiment). The labeled cells were rinsed 6 times with HBSS with calcium and magnesium (Thermo Scientific, Belfonte, PA) and the cell lysates were recovered in RIPA buffer with 1% of anti-protease inhibitor cocktail (Sigma-Aldrich, Saint Louis, MI), this was called the SuperSpike-in standard. In parallel, mMEEC were cultured in regular medium at ALI and with 200 μg/ml of NTHi lysates or control vehicle for 2 hours once and some cells were lysed 48hrs after. In some cells, at 48 hours, NTHi vs. control treatment was repeated for another 2 hours and some cells were then lysed at 96 hours. Finally, after 96 hours, NTHi vs. control treatment was repeated for another 2 hours and these cells were then recovered at 1 week (**Fig 1**). The cell lysates were recovered in RIPA buffer with 1% anti-protease inhibitors (Sigma-Aldrich, Saint Louis, MI). 30 μg of total proteins from not labeled cells (conditions of interest) were spiked with 30 μg of total proteins from labeled cells (SuperSpike-in standard) and processed for SDS-PAGE. In-gel digestion was performed cutting protein bands and LC–MS/MS was ran on the peptides generated as previously described[17,20]. The overall protocol and the protein quantification calculation are illustrated in the **Fig 2** and as previously described[21]. Briefly, raw files were processed for protein identification and quantification using Integrated Proteomics Pipeline (IP2) version 1.01 software developed by Integrated Proteomics Applications, Inc. (http://www.integratedproteomics.com/). Mass spectral data were uploaded into IP2 software and searched against the forward and reverse Uniprot murine database for tryptic peptides allowing two missed cleavages, possible modification of oxidized methionine (15.99492 Da) and heavy arginine (6.0204 Da) and heavy lysine (8.0142 Da). IP2 uses the Sequest 2010 (06_10_13_1836) search engine. Mass tolerance was set at ±30 ppm for MS and ±1.5 Da for MS/MS. Data were filtered based on a 1% protein false discovery rate. All the bands from each lane were summed in the analysis. Census software version 1.77, built into the IP2 platform, was used to determine the ratios of unlabeled and labeled peptide count (PC) pairs using an extracted chromatogram approach. Then each treatment ratio was divided by the corresponding control ratio, thereby cancelling out the SuperSILAC common denominator, and enabling comparison of the effect of NTHi treatment over time, with the number of PC corresponding to each identified protein specified. Proteins with a fold change +/- 2 were retained for further evaluation.

### Histopathological evaluation of middle ear cells grown at ALI

mMEEC cells were cultured on transwells at air-liquid interface and treated with serum free medium vs. 200 μg/ml NTHi lysates in serum free medium as explained before. For histology, cells were fixed with 10% formalin for 24h, embedded in paraffin and processed for H&E (hematoxylin and eosin) coloration by the Pathology service of Children’s National. The slides were observed with a BX51 Olympus microscope (Olympus, Rocklin, CA) with the objective 40X. The anti-pan Cytokeratin antibody [AE1/AE3] (ab27988) (Abcam, Cambridge, MA) at 1:1000 was used for immunohistochemical staining of epithelial keratins by the Pathology Service of Children’s National. For quantification of thickness the epithlelial layer was measured in 3 different areas per slide at 20x magnification. There were 5 slides per condition.

### Western blot analyses

A previously described protocol was used for the western blotting[22] of selected proteins for validation. Briefly, 15μl of each effusion sample was separated by electrophoresis on NuPAGE Novex 4–12% Bis-Tris gels (Life technologies, Carlsbad, CA). The molecular weight marker Kaleidoscope was used as a standard (Bio-Rad, Hercules, CA). The proteins were then transferred to a nitrocellulose membrane (Invitrogen). Membranes were blocked with 5% non-fat dry milk in PBS with 0.05% Tween-20 (PBST), and incubated used to incubate primary anti-mouse antibodies for Plakoglobin (PG-11E4, Thermo Scientific, Waltham, MA), periplakin (C-20, Santa Cruz, Dallas, TX), Histone H1 (C-17, Santa Cruz, Dallas, TX), keratin 10 (K-14, Santa Cruz, Dallas, TX), keratin 15 (G-12, Santa Cruz, Dallas, TX). Secondary antibody anti-goat at 1:5000 coupled to horseradish peroxidase (SigmaAldrich, St. Louis, MO). Detection was performed with a SuperSignal® West Dura Extended Duration Substrate kit (Pierce, Rockland, IL) according to the manufacturer’s instructions.

### Statistical analysis

The statistical difference between experimental and control groups for all experiments was determined by two-tailed paired Student T-tests. Significance level was set at p<0.05.

## Results

### SILAC

Mass spectrometry analysis identified and quantitated 2565 proteins across samples, of these 1596 proteins were identified with a total peptide count of at least 6 across all samples (**S1 Table**). The 30 most overall abundant proteins identified were not regulated by NTHi treatment across time points. Clathrin heavy chain, a peptide implicated in the formation of vesicles, was the most abundant protein overall. Several cytoskeletal and cytoskeletal binding proteins also demonstrated a high PC, such as actin, spectrin, filamin, plectin and myosin-9. Two heat shock proteins (HSP), HSP71 and HSP90 were detected in high abundance, as well as ubiquitin proteins (polyubiquitin-B and polyubiquitin-C) and metabolic enzymes (pyruvate kinase isoenzymes M1/M2 and ATP synthase).

Among the 1596 proteins detected, 74 proteins exhibited differential enrichment or depletion in cell lysates (+/-2.0 fold-change; p value<0.05) in at least one of the time points with NTHi lysates relative to control.

In order to better understand the intracellular effects of NTHi over time we analyzed the most dysregulated proteins in mMEEC at each time point, with the highest PC. **Table 1** lists the 35 most abundant proteins that were either significantly enriched or depleted across time points (p<0.05). Among these 35 proteins, 14 were enriched and 21 were depleted by NTHi lysates treatment. At 48 hours, the most abundant proteins that were statistically enriched by NTHi lysate treatment were the cytokeratin subtypes keratins 16, 6, 75, 17, 15, 79 and 5. Collectively, this group of cytokeratins is typically expressed during epithelial basal layer activation and cellular proliferation. Other mediators significantly up-regulated at 48 hours included CD44, Na+/H+ exchange regulatory co-factor, and Nuclear Pore Complex Protein 133. At 96 hours, ferritin, CD-44, and gamma-synuclein were key mediators enriched while Histone H1, involucrin, plakoglobin, periplakin, and retinal dehydrogenase were key mediators depleted at 96 hours. At 1 week, a majority significantly regulated proteins were depleted. Notably, keratin 10, a marker for suprabasal terminal differentiation and keratinization, was one of the few, (and the most) enriched mediator at 1 week. Depleted proteins again included many of the plakin group of proteins, involucrin, and Histone H1 along with nucleolin, and glutahione transferase.

**Table 1:** 35 most abundant proteins significantly regulated by NTHi lysate treatment.

Protein symbol	Description	Total peptide counts	N/C 48 hrs	N/C 96 hrs	N/C 1 wk
K2C6A	Keratin, type II cytoskeletal 6A	376	**2.32**	0.93	0.91
K1C17	Keratin, type I cytoskeletal 17	314	**2.19**	1.14	0.95
K2C5	Keratin, type II cytoskeletal 5	303	**2.01**	0.91	0.83
K2C75	Keratin, type II cytoskeletal 75	196	**2.31**	0.94	0.99
K1C15	Keratin, type I cytoskeletal 15	169	**2.00**	1.00	0.80
K1C16	Keratin, type I cytoskeletal 16	106	**2.41**	1.25	ND
SYUG	Gamma-synuclein	56	**1.66**	**1.73**	2.40
K2C79	Keratin, type II cytoskeletal 79	36	**2.19**	1.04	**0.67**
FRIL1	Ferritin light chain 1	34	1.15	**2.27**	1.89
PURB	Transcriptional activator protein Pur-beta	32	0.97	3.02	0.94
CD44	CD44 antigen	24	**2.09**	**1.57**	1.15
K1C10	Keratin, type I cytoskeletal 10	24	1.55	1.00	16.51
NHRF1	Na(+)/H(+) exchange regulatory cofactor NHE-RF1	19	**2.12**	0.80	0.90
NU133	Nuclear pore complex protein Nup133	15	**1.19**	ND	6.53
PEPL	Periplakin	327	**0.76**	**0.75**	**0.50**
NUCL	Nucleolin	283	1.04	0.95	**0.47**
EVPL	Envoplakin	98	**0.68**	**0.85**	**0.44**
H12	Histone H1.2	85	0.88	**0.29**	**0.36**
H13	Histone H1.3	85	0.88	0.50	**0.36**
LEG3	Galectin-3	74	0.98	**0.80**	**0.50**
GSTM2	Glutathione S-transferase Mu 2	73	0.87	**0.66**	**0.41**
H14	Histone H1.4	72	**0.85**	0.42	**0.36**
INVO	Involucrin	68	**0.59**	**0.45**	**0.29**
PLAK	Junction plakoglobin	61	**0.84**	**0.51**	**0.52**
PURA2	Adenylosuccinate synthetase isozyme 2	55	0.91	**0.77**	**0.48**
AL1A1	Retinal dehydrogenase 1	54	**0.60**	**0.51**	**0.25**
GSTM1	Glutathione S-transferase Mu 1	52	**0.67**	**0.59**	**0.29**
G6PD1	Glucose-6-phosphate 1-dehydrogenase X	48	**0.79**	**0.81**	**0.48**
CES1D	Carboxylesterase 1D	32	0.53	0.67	**0.35**
EPIPL	Epiplakin	29	1.82	**0.49**	0.50
RMXL1	RNA binding motif protein, X-linked-like-1	25	1.12	**1.16**	**0.41**
H11	Histone H1.1	21	0.00	**0.18**	0.44
PP2BA	Serine/threonine-protein phosphatase 2B catalytic subunit alpha isoform	20	0.45	0.85	**0.72**
5NTD	5'-nucleotidase	18	0.84	**0.42**	0.29
HNRPC	Heterogeneous nuclear ribonucleoproteins C1/C2	16	ND	**0.82**	0.38

Fold change ratios represent treated with NTHi lysates over control (N/C). (n = 6 replicates for each condition; bolded numbers represent p<0.05)

**Fig 3:**
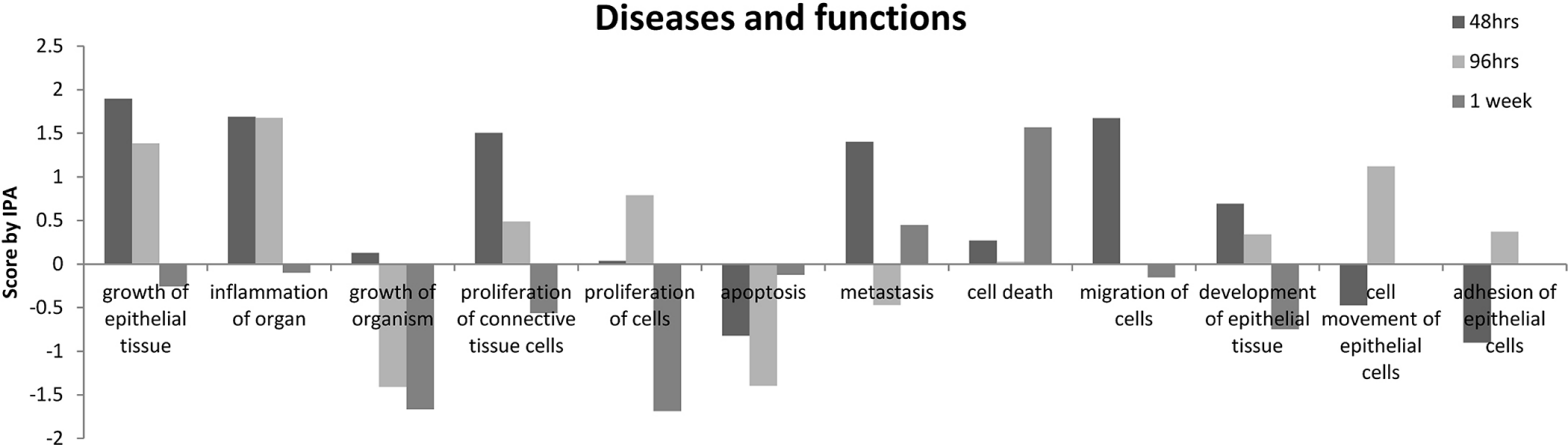
Diseases and biological functions associated with conditional proteomics results. The diseases and functions associated with the expression patterns of the regulated proteins during the treatment were generated by Ingenuity Pathway Analysis and scored.

The diseases and biological functions associated with our conditional proteomics results were then generated through the use of QIAGEN’s Ingenuity Pathway Analysis (IPA® QIAGEN Redwood City, www.qiagen.com/ingenuity). **Fig 3** demonstrates results of this analysis. At early time points (48 and 96 hours) there was increase noted in “growth of epithelial tissue”, “inflammation of organ”, “proliferation of connective tissue/cells, and “metastasis” with a decrease in “apoptosis”. At the late time point (1 week), no biological functions were noted to be increased, however there were decreases in “proliferation of connective tissue/cells”, “growth of epithelial tissue”, and “inflammation”.

### Histology of mMEEC grown at ALI

**Fig 4:**
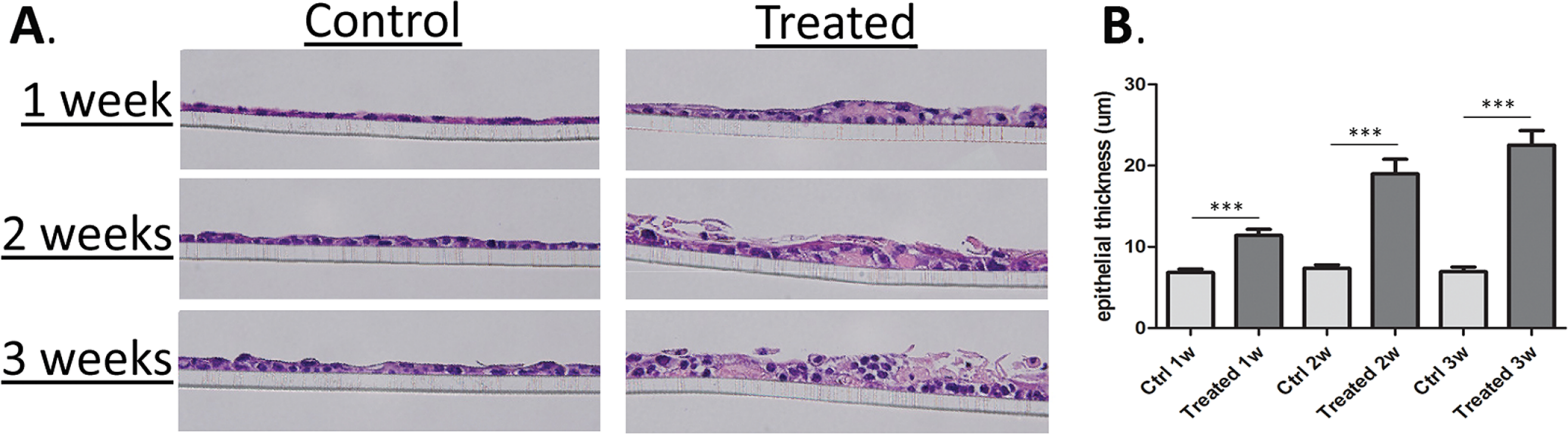
Histopathology of mMEEC at ALI exposed to NTHi lysates over time. A. mMEEC were differentiated and treated or not with NTHi lysates 3 times a week for 1, 2 or 3 consecutive weeks. At the end of the treatments, the cells were fixed 24 hours in 10% formalin, dehydrated, embedded in paraffin, cut and stained by the Pathology Service of Children’s National Health System. B. Measurements of epithelial thickness demonstrated statistically significant increases at each time point relative to control (***p<0.001).

**Fig 5:**
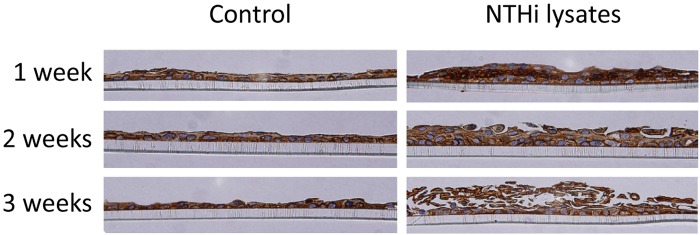
Pan cytokertin expression in mMEEC. Anti-pan Cytokeratin antibody [AE1/AE3] (ab27988) was used to identify localization of epithelial keratins. With treatment, an abundance is seen at 1 week in the suprabasal staining. Over time the epithelium gets looser, with release of keratin from the epithelial surface.

mMEEC were cultured at ALI and treated until longer time points (1, 2 and 3 weeks) to better observe epithelium remodeling. From 1–3 weeks of NTHi exposure there is a progressive and marked degree in the thickness of the epithelial layer, with the lysates inducing a dramatic 2–3 fold increase in the thickness of mMEEC cultures at ALI over control (**Fig 4**). Moreover, the histology demonstrates a significant difference in the organization of the epithelium, with the treated epithelium looking progressively “looser”, with increased intracellular spaces, along with the deposition of cellular debris and keratinacious material on the surface (**Fig 5**).

### Western blots

**Fig 6:**
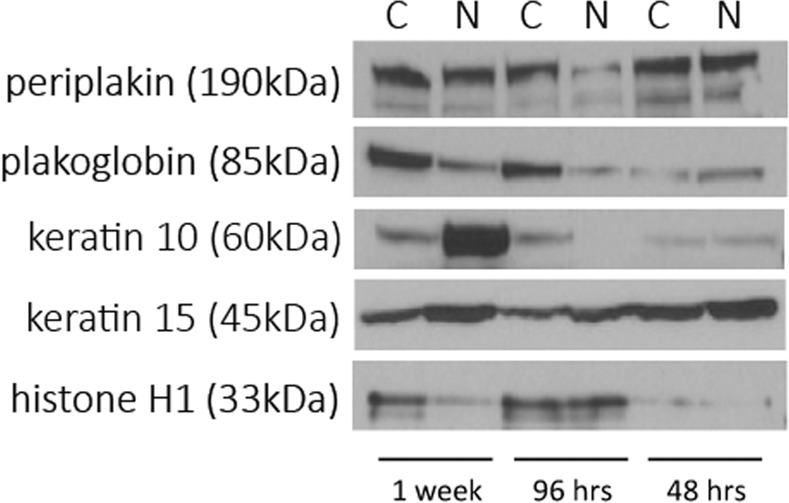
Western Blots for selected proteins of note. C = control, N = NTHI lysates, note the times are depicted as 1 week at the left, 96 hours in the middle, and 48 hours on the right of the images.

On order to validate the SILAC findings, western blots were performed and added as **Fig 6**. Five separate mediators found to significantly change by SILAC were assayed by immunoblotting. We noted that keratin 15- a proliferative keratin, overall mildly increased with NTHi lysates by Western blot at all time points. Keratin 10-a differentiation keratin, was found to markedly increase at 1 week with treatment, consistent with the 16-fold change noted on SILAC. Periplakin, plakoglobin, and hsitone H1, were all found to decrease with NTHi treatment at 96 hours and 1 week, also consistent with the quantitative proteomics result.

## Discussion

A key component of OM progression from acute to chronic is epithelial remodeling during middle ear inflammation, or upon bacterial exposure. Normally middle ear mucosa is comprised by a simple squamous epithelium, which during the acutely infected and inflamed state has the capacity to grow and proliferate to many times its original thickness, into a thick, often pseudostratified, columnar epithelial complex[7,23]. Several groups including ours have demonstrated that the middle ear infection in animals by *NTHi*, *S*. *Pneumoniae*, or lipopolysaccharide results in middle ear mucous metaplasia similar to that seen in human tissue samples[12,13,24,25]. To our knowledge, no groups have directly studied these events *in vitro* using middle ear cell lines. Characterizing and quantifying intracellular changes at the protein level during the process of bacterially induced mucosal hyperplasia, could help elucidate key mechanisms contributing to the progression of OM. This was the goal of our study and we are the first group using a differentiated middle ear epithelial cell culture model permitting chronic exposures to bacterial components over long periods of time. We are also the first ones employing powerful proteomics techniques such as SILAC to explore this question. Importantly we used immunoblotting to validate the SILAC results, and indeed found that SILAC appears to be more sensitive for quantification changes in protein levels than Western Blot. Large fold changes in protein levels are needed before obvious changes in western blot signal is noted (as is the case for keratin 10 at one week).

Our time course conditional quantitative proteomics data of abundant intracellular proteins uncovered some interesting and key findings, the first one being the regulation of the keratins by NTHi lysates treatment in mMEEC. First, at 48 hours Keratins (K) 16, 6, 75, 17, 15 and 5 are overabundant after NTHi treatment relative to control. As part of the epithelial cytoskeleton, keratins are important for the mechanical stability and integrity of epithelial cells and tissues[26]. Importantly, keratin expression patterns not only characterize cells as “epithelial”, they are also characteristic for distinct stages during cellular epithelial differentiation. K17/16/15/5 are all expressed in the basal layer of epithelia. K5 is strongly expressed in the undifferentiated basal cell layer containing the stem cells, is down-regulated in the differentiating suprabasal cell layers and is known to be absent in simple/one-layered epithelia[27]. Similarly K15 is known to be restricted in expression to the basal layer of stratified epithelium[28]. K16 and 6 are both constitutive keratins of stratified epithelia built up by cells of relatively high proliferative state. They are known to be induced during pulmonary respiratory epithelium metaplasia[29,30]. K17 is also a marker of “activated” basal epithelium, and is inducible upon cell injury an event critical in epithelial wound injury repair. K17 knockout mouse embryos show a delay in the closure of surface ectoderm wounds [31]. K16/6/17 are all inducible upon stress, injury, or inflammation, and as it is not surprising that squamous cell carcinomas express these three keratins[32]. Sparse expression of K75, typically a hair follicle keratin, has also been identified in proliferative tissues, such as squamous cell carcinoma[26]. Collectively, the enriched finding of these keratin sub-types in bacterially challenged middle ear epithelium is highly suggestive of basal cell activation and proliferation upon NTHi exposure. Conversely, K10 which we identified to be significantly upregulated at the 1 week time point, is typically expressed in suprabasal epithelial layers, and represents inhibited cell proliferation and decreased cell cycle progression[33,34]. A loss of K10 increases keratinocyte turnover, and as such K10 is thought to represent the major keratin of epithelial cell terminal differentiation and keratinization[26]. Conclusively, our data show that NTHi induces a transition of the epithelium ranging from activation of proliferation of epithelial cells at early time points, to terminal differentiation of suprabasal cell layers at chronic time points, highlighted by a profound change in keratin subtype expression.

Along the same lines, a second key finding in our data is the collective depletion of the plakin group of proteins, including periplakin (0.5 fold change at 1 week), envoplakin (0.44 fold change at 1 week), junction plakoglobin (0.51 fold change at 96 hours) and epiplakin (0.49 and 0.50 fold change at 96 hours and 1 week respectively). The plakins are a family of proteins that crosslink cytoskeletal filaments and attach them to membrane-associated complexes at cell junctions. As such, they play an important role in maintaining the integrity of the epithelial layer and comprise the molecular link between the cytoskeleton and the extracellular matrix. Periplakin and envoplakin have been identified as important elements of the epithelial cornified layer and of desmosomes. Together with epiplakin they function to bundle and assemble intermediate filaments[35,36]. Plakoglobin is a key constituent of desmosomes, which are important cell to cell adhesion complexes. Plakoglobin is the linker between intracellular intermediate filaments and the desmoplakin molecule of the desmosome complex[36]. Without these important linkers epithelial organization and integrity is affected. Knockout mice deficient in involucrin, envoplakin, and periplakin have a markedly defective ultrastructure of epithelium, with impairment of the epidermal barrier, and a change in the T-cell composition of their skin[37]. This goes along with the findings of our histopathology showing that with bacterial treatment, although the epithelium is thicker, there is a dramatic increase in intracellular spaces and breaks in the layer, giving the appearance of a ‘looser’ lining.

A third significant finding of our SILAC data was the consistently decreased levels of Histone H1 at 96 hours and 1 week. Histones are highly conserved proteins which primarily function to form nucleosomes and compact chromatin in the cell. Recent evidence also has found that histones can be released extracellularily where they act as key pro-inflammatory mediators in a plethora of disease processes such as sepsis[38]. H1 specifically is a linker histone in chromatin (not a core histone), and is known to be released from cells upon injury and apoptosis. As such, it is quite possible that the histones are being released from the epithelium over time upon the bacterial insult, and therefore they were found to be depleted in the cell lysates.

The protein gamma-synuclein was also shown to be induced by NTHi lysates at all time points. This protein has been suggested to modulate the keratin network and to play a role in cell adhesion and microtubule structure[39], which seems relevant with the keratin regulation discussed above. The silencing of the gamma-synuclein gene was also shown to inhibit cell proliferation and migration[40], which goes along with the differentiation hypothesis explained before. Interestingly, nucleolin was also downregulated at 1 week of exposure, this protein is the major nuclear protein of eukaryotic cells, and is a chaperone of histones; its overexpression is characterized in proliferative cancer cells[41]. Finally, results uncovered the regulation of several glutathione-S-transferases (mu 1, mu 2 and A3) that are antioxidant proteins and the galectin-3 that has a high affinity to the beta galactosidases of bacteria. They are all downregulated especially at 1 week, which might signify an immunosuppressor effect of NTHi lysates on mMEEC.

## Conclusion

In conclusion, quantitative proteomics reveals that NTHi lysates drive events resulting in cell remodeling in murine middle ear epithelium. The remodeling is characterized by an initial proliferative response followed by terminal differentiation and decreased epithelial integrity. These factors all play a role in observed epithelial hyperplasia in OM progression from acute to chronic. Further elucidation of these mediators will be critical in understanding this progression at the molecular level.

## Supporting Information

S1 Table Mass spectrometry of identified proteins in mMEEC expressed as peptide counts (PC) and ratios treated with NTHi lysates over control (L/C). (DOC)

## References

[1] Cherry DK, Woodwell DA (2002) National Ambulatory Medical Care Survey: 2000 summary. Adv Data: 1–32. 12661586

[2] Ahmed S, Shapiro NL, Bhattacharyya N Incremental health care utilization and costs for acute otitis media in children. Laryngoscope 124: 301–305. 10.1002/lary.24190 23649905

[3] Rosenfeld RM, Casselbrant ML, Hannley MT (2001) Implications of the AHRQ evidence report on acute otitis media. Otolaryngol Head Neck Surg 125: 440–448; discussion 439. 11700439 10.1067/mhn.2001.119326

[4] Media AAoPSoMoAO (2004) Diagnosis and management of acute otitis media. Pediatrics 113: 1451–1465. 15121972 10.1542/peds.113.5.1451

[5] Effusion AAoPSoOMW (2004) Otitis media with effusion. Pediatrics 113: 1412–1429. 15121966 10.1542/peds.113.5.1412

[6] Bluestone CD, Stool SE, Kenna MA (1996) Pediatric otolaryngology Philadelphia: Saunders.

[7] Lim DJ, Birck H (1971) Ultrastructural pathology of the middle ear mucosa in serous otitis media. Ann Otol Rhinol Laryngol 80: 838–853. 5127754 10.1177/000348947108000611

[8] Tos M, Bak-Pedersen K (1977) Goblet cell population in the pathological middle ear and eustachian tube of children and adults. Ann Otol Rhinol Laryngol 86: 209–218. 848832 10.1177/000348947708600212

[9] Ryan AF, Jung TT, Juhn SK, Li JD, Andalibi A, Lin J, et al (2005) Recent advances in otitis media. 4A. Molecular biology. Ann Otol Rhinol Laryngol Suppl 194: 42–49. 15700934

[10] Casey JR, Pichichero ME (2004) Changes in frequency and pathogens causing acute otitis media in 1995–2003. Pediatr Infect Dis J 23: 824–828. 15361720 10.1097/01.inf.0000136871.51792.19

[11] Klein JO (2002) What's new in the diagnosis and management of otitis media? Pediatr Ann 31: 777–778. 12503435 10.3928/0090-4481-20021201-06

[12] Maeda K, Hirano T, Ichimiya I, Kurono Y, Suzuki M, Mogi G (2004) Cytokine expression in experimental chronic otitis media with effusion in mice. Laryngoscope 114: 1967–1972. 15510024 10.1097/01.mlg.0000147930.29261.51

[13] Preciado D, Burgett K, Ghimbovschi S, Rose M NTHi induction of Cxcl2 and middle ear mucosal metaplasia in mice. Laryngoscope 123: E66–71. 10.1002/lary.24097 23553435 PMC3740030

[14] Lee HY, Takeshita T, Shimada J, Akopyan A, Woo JI, Pan H, et al (2008) Induction of beta defensin 2 by NTHi requires TLR2 mediated MyD88 and IRAK-TRAF6-p38MAPK signaling pathway in human middle ear epithelial cells. BMC Infect Dis 8: 87 10.1186/1471-2334-8-87 18578886 PMC2447838

[15] Moghaddam SJ, Clement CG, De la Garza MM, Zou X, Travis EL, Young HW, et al (2008) Haemophilus influenzae lysate induces aspects of the chronic obstructive pulmonary disease phenotype. Am J Respir Cell Mol Biol 38: 629–638. 18096867 10.1165/rcmb.2007-0366OC PMC2396243

[16] Preciado D PM, Tsai S, Tomney A, Brown K, Val S. (2015) A Proteomic Characterization of NTHi Lysates. International Journal of Pediatric Otorhinolaryngology *in press*. 10.1016/j.ijporl.2015.11.016 PMC4706994 26746604

[17] Val S, Mubeen H, Tomney A, Chen S, Preciado D Impact of Staphylococcus epidermidis lysates on middle ear epithelial proinflammatory and mucogenic response. J Investig Med 63: 258–266. 10.1097/JIM.0000000000000127 25503091

[18] Tsuchiya K, Kim Y, Ondrey FG, Lin J (2005) Characterization of a temperature-sensitive mouse middle ear epithelial cell line. Acta Otolaryngol 125: 823–829. 16158528 10.1080/00016480510031533

[19] Preciado D, Lin J, Wuertz B, Rose M (2008) Cigarette smoke activates NF kappa B and induces Muc5b expression in mouse middle ear cells. Laryngoscope 118: 464–471. 18091336 10.1097/MLG.0b013e3185aedc7 PMC2692718

[20] Preciado D, Goyal S, Rahimi M, Watson AM, Brown KJ, Hathout Y, et al (2010) MUC5B Is the Predominant Mucin Glycoprotein in Chronic Otitis Media Fluid. Pediatr Res 68: 231–236. 10.1203/00006450-201011001-00451 20531251 PMC3679171

[21] Brown KJ, Seol H, Pillai DK, Sankoorikal BJ, Formolo CA, Mac J, et al The human secretome atlas initiative: implications in health and disease conditions. Biochim Biophys Acta 1834: 2454–2461. 10.1016/j.bbapap.2013.04.007 PMC3755092 23603790

[22] Wu X, Peters-Hall JR, Ghimbovschi S, Mimms R, Rose MC, Pena MT Glandular gene expression of sinus mucosa in chronic rhinosinusitis with and without cystic fibrosis. Am J Respir Cell Mol Biol 45: 525–533. 10.1165/rcmb.2010-0133OC 21177983 PMC3175585

[23] Ryan AF, Baird A (1993) Growth factors during proliferation of the middle ear mucosa. Acta Otolaryngol 113: 68–74. 8442425 10.3109/00016489309135769

[24] Tsuboi Y, Kim Y, Giebink GS, Le C, Paparella MM, Chen N, et al (2002) Induction of mucous cell metaplasia in the middle ear of rats using a three-step method: an improved model for otitis media with mucoid effusion. Acta Otolaryngol 122: 153–160. 11936906 10.1080/00016480252814153

[25] Caye-Thomasen P, Tos M (2002) Histopathologic differences due to bacterial species in acute otitis media. Int J Pediatr Otorhinolaryngol 63: 99–110. 11955601 10.1016/s0165-5876(01)00641-3

[26] Moll R, Divo M, Langbein L (2008) The human keratins: biology and pathology. Histochem Cell Biol 129: 705–733. 10.1007/s00418-008-0435-6 18461349 PMC2386534

[27] Moll R, Dhouailly D, Sun TT (1989) Expression of keratin 5 as a distinctive feature of epithelial and biphasic mesotheliomas. An immunohistochemical study using monoclonal antibody AE14. Virchows Arch B Cell Pathol Incl Mol Pathol 58: 129–145. 2482572 10.1007/BF02890064

[28] Waseem A, Dogan B, Tidman N, Alam Y, Purkis P, Jackson S, et al (1999) Keratin 15 expression in stratified epithelia: downregulation in activated keratinocytes. J Invest Dermatol 112: 362–369. 10084315 10.1046/j.1523-1747.1999.00535.x

[29] Stosiek P, Kasper M, Moll R (1992) Changes in cytokeratin expression accompany squamous metaplasia of the human respiratory epithelium. Virchows Arch A Pathol Anat Histopathol 421: 133–141. 1381128 10.1007/BF01607046

[30] Leube RE, Rustad TJ (1991) Squamous cell metaplasia in the human lung: molecular characteristics of epithelial stratification. Virchows Arch B Cell Pathol Incl Mol Pathol 61: 227–253. 1723555 10.1007/BF02890425

[31] Mazzalupo S, Wong P, Martin P, Coulombe PA (2003) Role for keratins 6 and 17 during wound closure in embryonic mouse skin. Dev Dyn 226: 356–365. 12557214 10.1002/dvdy.10245

[32] Chu PG, Weiss LM (2002) Keratin expression in human tissues and neoplasms. Histopathology 40: 403–439. 12010363 10.1046/j.1365-2559.2002.01387.x

[33] Paramio JM (1999) A role for phosphorylation in the dynamics of keratin intermediate filaments. Eur J Cell Biol 78: 33–43. 10082422 10.1016/S0171-9335(99)80005-3

[34] Koch PJ, Roop DR (2004) The role of keratins in epidermal development and homeostasis—going beyond the obvious. J Invest Dermatol 123: x–xi. 15482464 10.1111/j.0022-202X.2004.23495.x

[35] Ruhrberg C, Watt FM (1997) The plakin family: versatile organizers of cytoskeletal architecture. Curr Opin Genet Dev 7: 392–397. 9229116 10.1016/s0959-437x(97)80154-2

[36] Jefferson JJ, Leung CL, Liem RK (2004) Plakins: goliaths that link cell junctions and the cytoskeleton. Nat Rev Mol Cell Biol 5: 542–553. 15232572 10.1038/nrm1425

[37] Sevilla LM, Nachat R, Groot KR, Klement JF, Uitto J, Djian P, et al (2007) Mice deficient in involucrin, envoplakin, and periplakin have a defective epidermal barrier. J Cell Biol 179: 1599–1612. 10.1083/jcb.200706187 18166659 PMC2373502

[38] Xu J, Zhang X, Pelayo R, Monestier M, Ammollo CT, Semeraro F, et al (2009) Extracellular histones are major mediators of death in sepsis. Nat Med 15: 1318–1321. 10.1038/nm.2053 19855397 PMC2783754

[39] Ninkina NN, Privalova EM, Pinon LG, Davies AM, Buchman VL (1999) Developmentally regulated expression of persyn, a member of the synuclein family, in skin. Exp Cell Res 246: 308–311. 9925745 10.1006/excr.1998.4292

[40] He J, Xie N, Yang J, Guan H, Chen W, Wu H, et al siRNA-Mediated Suppression of Synuclein gamma Inhibits MDA-MB-231 Cell Migration and Proliferation by Downregulating the Phosphorylation of AKT and ERK. J Breast Cancer 17: 200–206. 10.4048/jbc.2014.17.3.200 25320617 PMC4197349

[41] Storck S, Shukla M, Dimitrov S, Bouvet P (2007) Functions of the histone chaperone nucleolin in diseases. Subcell Biochem 41: 125–144. 17484127 10.1007/1-4020-5466-1_7

