# Supplementary material for: SuperSILAC Quantitative Proteome Profiling of Murine Middle Ear Epithelial Cell Remodeling with NTHi
Source: PLoS One. 2016 Feb 9;11(2):e0148612. doi: 10.1371/journal.pone.0148612 (PMC4747582; doi:10.1371/journal.pone.0148612)
Supplement: S1 Table — (DOC) [file pone.0148612.s001.doc]

| locus | description | Total PC | L/C 48 | L/C 96 | L/C week |
| --- | --- | --- | --- | --- | --- |
| sp|Q68FD5|CLH_MOUSE | Clathrin heavy chain 1 OS=Mus musculus GN=Cltc PE=1 SV=3 | 960 | 1.056 | 0.943 | 0.881 |
| sp|Q8VDD5|MYH9_MOUSE | Myosin-9 OS=Mus musculus GN=Myh9 PE=1 SV=4 | 757 | 1.099 | 0.955 | 0.957 |
| sp|Q9QXS1|PLEC_MOUSE | Plectin OS=Mus musculus GN=Plec PE=1 SV=2 | 683 | 1.533 | 1.156 | 0.938 |
| sp|P48678|LMNA_MOUSE | Prelamin-A/C OS=Mus musculus GN=Lmna PE=1 SV=2 | 662 | 1.000 | 0.917 | 0.934 |
| sp|P16546|SPTN1_MOUSE | Spectrin alpha chain, non-erythrocytic 1 OS=Mus musculus GN=Sptan1 PE=1 SV=4 | 657 | 0.906 | 0.860 | 0.706 |
| sp|P10107|ANXA1_MOUSE | Annexin A1 OS=Mus musculus GN=Anxa1 PE=1 SV=2 | 651 | 0.870 | 0.831 | 0.660 |
| sp|Q8BTM8|FLNA_MOUSE | Filamin-A OS=Mus musculus GN=Flna PE=1 SV=5 | 641 | 1.378 | 1.232 | 1.175 |
| sp|P62983|RS27A_MOUSE | Ubiquitin-40S ribosomal protein S27a OS=Mus musculus GN=Rps27a PE=1 SV=2 | 613 | 1.105 | 0.886 | 0.933 |
| sp|P10126|EF1A1_MOUSE | Elongation factor 1-alpha 1 OS=Mus musculus GN=Eef1a1 PE=1 SV=3 | 595 | 1.082 | 0.962 | 0.851 |
| sp|Q9JKF1|IQGA1_MOUSE | Ras GTPase-activating-like protein IQGAP1 OS=Mus musculus GN=Iqgap1 PE=1 SV=2 | 554 | 1.065 | 0.942 | 0.869 |
| sp|P63017|HSP7C_MOUSE | Heat shock cognate 71 kDa protein OS=Mus musculus GN=Hspa8 PE=1 SV=1 | 527 | 1.067 | 0.991 | 0.887 |
| sp|P11499|HS90B_MOUSE | Heat shock protein HSP 90-beta OS=Mus musculus GN=Hsp90ab1 PE=1 SV=3 | 508 | 1.126 | 1.008 | 0.951 |
| sp|P0CG49|UBB_MOUSE | Polyubiquitin-B OS=Mus musculus GN=Ubb PE=1 SV=1 | 493 | 1.105 | 0.000 | 0.933 |
| sp|P62984|RL40_MOUSE | Ubiquitin-60S ribosomal protein L40 OS=Mus musculus GN=Uba52 PE=1 SV=2 | 493 | 1.105 | 0.000 | 0.933 |
| sp|P0CG50|UBC_MOUSE | Polyubiquitin-C OS=Mus musculus GN=Ubc PE=1 SV=2 | 493 | 1.100 | 0.000 | 0.941 |
| sp|P07901|HS90A_MOUSE | Heat shock protein HSP 90-alpha OS=Mus musculus GN=Hsp90aa1 PE=1 SV=4 | 480 | 1.096 | 0.958 | 0.899 |
| sp|Q9JHU4|DYHC1_MOUSE | Cytoplasmic dynein 1 heavy chain 1 OS=Mus musculus GN=Dync1h1 PE=1 SV=2 | 464 | 1.032 | 0.905 | 0.706 |
| sp|P52480|KPYM_MOUSE | Pyruvate kinase isozymes M1/M2 OS=Mus musculus GN=Pkm PE=1 SV=4 | 455 | 1.134 | 1.032 | 0.898 |
| sp|P57780|ACTN4_MOUSE | Alpha-actinin-4 OS=Mus musculus GN=Actn4 PE=1 SV=1 | 432 | 1.022 | 1.040 | 0.751 |
| sp|P56480|ATPB_MOUSE | ATP synthase subunit beta, mitochondrial OS=Mus musculus GN=Atp5b PE=1 SV=2 | 400 | 0.994 | 0.910 | 0.828 |
| sp|O09131|GSTO1_MOUSE | Glutathione S-transferase omega-1 OS=Mus musculus GN=Gsto1 PE=2 SV=2 | 397 | 0.945 | 0.808 | 0.726 |
| sp|P20029|GRP78_MOUSE | 78 kDa glucose-regulated protein OS=Mus musculus GN=Hspa5 PE=1 SV=3 | 395 | 1.189 | 1.078 | 0.893 |
| sp|P51881|ADT2_MOUSE | ADP/ATP translocase 2 OS=Mus musculus GN=Slc25a5 PE=1 SV=3 | 392 | 0.971 | 0.882 | 0.815 |
| sp|P58252|EF2_MOUSE | Elongation factor 2 OS=Mus musculus GN=Eef2 PE=1 SV=2 | 377 | 1.130 | 1.026 | 0.920 |
| sp|P50446|K2C6A_MOUSE | Keratin, type II cytoskeletal 6A OS=Mus musculus GN=Krt6a PE=2 SV=3 | 376 | 2.315 | 0.934 | 0.905 |
| sp|Q69ZN7|MYOF_MOUSE | Myoferlin OS=Mus musculus GN=Myof PE=1 SV=2 | 369 | 1.022 | 0.914 | 0.629 |
| sp|Q61781|K1C14_MOUSE | Keratin, type I cytoskeletal 14 OS=Mus musculus GN=Krt14 PE=1 SV=2 | 368 | 1.887 | 0.952 | 0.706 |
| sp|P63260|ACTG_MOUSE | Actin, cytoplasmic 2 OS=Mus musculus GN=Actg1 PE=1 SV=1 | 356 | 1.019 | 0.986 | 0.766 |
| sp|P60710|ACTB_MOUSE | Actin, cytoplasmic 1 OS=Mus musculus GN=Actb PE=1 SV=1 | 356 | 1.025 | 0.993 | 0.767 |
| sp|Q62261|SPTB2_MOUSE | Spectrin beta chain, non-erythrocytic 1 OS=Mus musculus GN=Sptbn1 PE=1 SV=2 | 353 | 0.926 | 0.921 | 0.712 |
| sp|Q99P72|RTN4_MOUSE | Reticulon-4 OS=Mus musculus GN=Rtn4 PE=1 SV=2 | 351 | 1.037 | 0.882 | 0.779 |
| sp|P99024|TBB5_MOUSE | Tubulin beta-5 chain OS=Mus musculus GN=Tubb5 PE=1 SV=1 | 344 | 1.174 | 1.018 | 0.960 |
| sp|P68372|TBB4B_MOUSE | Tubulin beta-4B chain OS=Mus musculus GN=Tubb4b PE=1 SV=1 | 342 | 1.105 | 1.009 | 0.971 |
| sp|P07356|ANXA2_MOUSE | Annexin A2 OS=Mus musculus GN=Anxa2 PE=1 SV=2 | 339 | 1.000 | 0.891 | 0.807 |
| sp|Q03265|ATPA_MOUSE | ATP synthase subunit alpha, mitochondrial OS=Mus musculus GN=Atp5a1 PE=1 SV=1 | 336 | 1.109 | 0.926 | 0.826 |
| sp|Q7TPR4|ACTN1_MOUSE | Alpha-actinin-1 OS=Mus musculus GN=Actn1 PE=1 SV=1 | 334 | 1.107 | 0.991 | 0.925 |
| sp|Q80X90|FLNB_MOUSE | Filamin-B OS=Mus musculus GN=Flnb PE=1 SV=3 | 330 | 1.347 | 1.187 | 1.160 |
| sp|Q9R269|PEPL_MOUSE | Periplakin OS=Mus musculus GN=Ppl PE=1 SV=1 | 327 | 0.765 | 0.747 | 0.503 |
| sp|P68373|TBA1C_MOUSE | Tubulin alpha-1C chain OS=Mus musculus GN=Tuba1c PE=1 SV=1 | 316 | 1.181 | 1.055 | 0.971 |
| sp|Q9QWL7|K1C17_MOUSE | Keratin, type I cytoskeletal 17 OS=Mus musculus GN=Krt17 PE=1 SV=3 | 314 | 2.194 | 1.140 | 0.953 |
| sp|P38647|GRP75_MOUSE | Stress-70 protein, mitochondrial OS=Mus musculus GN=Hspa9 PE=1 SV=3 | 311 | 1.050 | 0.933 | 0.844 |
| sp|Q01853|TERA_MOUSE | Transitional endoplasmic reticulum ATPase OS=Mus musculus GN=Vcp PE=1 SV=4 | 310 | 1.127 | 1.115 | 0.989 |
| sp|Q922U2|K2C5_MOUSE | Keratin, type II cytoskeletal 5 OS=Mus musculus GN=Krt5 PE=1 SV=1 | 303 | 2.011 | 0.914 | 0.831 |
| sp|P19096|FAS_MOUSE | Fatty acid synthase OS=Mus musculus GN=Fasn PE=1 SV=2 | 296 | 1.045 | 0.870 | 0.674 |
| sp|P51125|ICAL_MOUSE | Calpastatin OS=Mus musculus GN=Cast PE=1 SV=2 | 296 | 1.138 | 0.911 | 0.753 |
| sp|P48962|ADT1_MOUSE | ADP/ATP translocase 1 OS=Mus musculus GN=Slc25a4 PE=1 SV=4 | 295 | 1.007 | 0.936 | 0.833 |
| sp|P40142|TKT_MOUSE | Transketolase OS=Mus musculus GN=Tkt PE=1 SV=1 | 291 | 1.075 | 0.916 | 0.899 |
| sp|Q9Z331|K2C6B_MOUSE | Keratin, type II cytoskeletal 6B OS=Mus musculus GN=Krt6b PE=1 SV=3 | 287 | InD | 0.926 | 0.912 |
| sp|P09405|NUCL_MOUSE | Nucleolin OS=Mus musculus GN=Ncl PE=1 SV=2 | 283 | 1.038 | 0.952 | 0.469 |
| sp|Q99K51|PLST_MOUSE | Plastin-3 OS=Mus musculus GN=Pls3 PE=1 SV=3 | 274 | 1.132 | 0.941 | 0.986 |
| sp|P08113|ENPL_MOUSE | Endoplasmin OS=Mus musculus GN=Hsp90b1 PE=1 SV=2 | 274 | 1.241 | 1.222 | 1.034 |
| sp|P63101|1433Z_MOUSE | 14-3-3 protein zeta/delta OS=Mus musculus GN=Ywhaz PE=1 SV=1 | 271 | 1.030 | 0.919 | 0.784 |
| sp|P68369|TBA1A_MOUSE | Tubulin alpha-1A chain OS=Mus musculus GN=Tuba1a PE=1 SV=1 | 271 | 1.144 | 1.063 | 1.019 |
| sp|P63038|CH60_MOUSE | 60 kDa heat shock protein, mitochondrial OS=Mus musculus GN=Hspd1 PE=1 SV=1 | 266 | 1.097 | 1.037 | 0.967 |
| sp|Q7TPV4|MBB1A_MOUSE | Myb-binding protein 1A OS=Mus musculus GN=Mybbp1a PE=1 SV=2 | 260 | 1.167 | 1.045 | 1.016 |
| sp|Q9CWF2|TBB2B_MOUSE | Tubulin beta-2B chain OS=Mus musculus GN=Tubb2b PE=1 SV=1 | 259 | 1.156 | 1.018 | 0.961 |
| sp|Q7TMM9|TBB2A_MOUSE | Tubulin beta-2A chain OS=Mus musculus GN=Tubb2a PE=1 SV=1 | 259 | 1.155 | 1.018 | 0.971 |
| sp|A2A863|ITB4_MOUSE | Integrin beta-4 OS=Mus musculus GN=Itgb4 PE=2 SV=1 | 253 | 1.014 | 0.877 | 0.758 |
| sp|P17182|ENOA_MOUSE | Alpha-enolase OS=Mus musculus GN=Eno1 PE=1 SV=3 | 253 | 1.113 | 0.974 | 0.876 |
| sp|Q02053|UBA1_MOUSE | Ubiquitin-like modifier-activating enzyme 1 OS=Mus musculus GN=Uba1 PE=1 SV=1 | 252 | 1.097 | 1.121 | 0.913 |
| sp|Q8BL66|EEA1_MOUSE | Early endosome antigen 1 OS=Mus musculus GN=Eea1 PE=2 SV=2 | 251 | 1.066 | 0.951 | 0.871 |
| sp|P23116|EIF3A_MOUSE | Eukaryotic translation initiation factor 3 subunit A OS=Mus musculus GN=Eif3a PE=1 SV=5 | 250 | 1.082 | 1.010 | 0.913 |
| sp|P68134|ACTS_MOUSE | Actin, alpha skeletal muscle OS=Mus musculus GN=Acta1 PE=1 SV=1 | 239 | 1.000 | 0.979 | 0.770 |
| sp|P68033|ACTC_MOUSE | Actin, alpha cardiac muscle 1 OS=Mus musculus GN=Actc1 PE=1 SV=1 | 239 | 1.013 | 0.986 | 0.771 |
| sp|Q8CGC7|SYEP_MOUSE | Bifunctional glutamate/proline--tRNA ligase OS=Mus musculus GN=Eprs PE=2 SV=4 | 238 | 1.049 | 0.953 | 0.963 |
| sp|P09411|PGK1_MOUSE | Phosphoglycerate kinase 1 OS=Mus musculus GN=Pgk1 PE=1 SV=4 | 234 | 1.127 | 1.000 | 0.875 |
| sp|Q6URW6|MYH14_MOUSE | Myosin-14 OS=Mus musculus GN=Myh14 PE=1 SV=1 | 232 | 0.989 | 0.855 | 0.695 |
| sp|P26039|TLN1_MOUSE | Talin-1 OS=Mus musculus GN=Tln1 PE=1 SV=2 | 225 | 1.095 | 0.982 | 0.800 |
| sp|P27773|PDIA3_MOUSE | Protein disulfide-isomerase A3 OS=Mus musculus GN=Pdia3 PE=1 SV=2 | 222 | 1.202 | 1.103 | 0.901 |
| sp|P05213|TBA1B_MOUSE | Tubulin alpha-1B chain OS=Mus musculus GN=Tuba1b PE=1 SV=2 | 221 | InD | 1.055 | 0.971 |
| sp|P17751|TPIS_MOUSE | Triosephosphate isomerase OS=Mus musculus GN=Tpi1 PE=1 SV=4 | 220 | 1.044 | 0.986 | 0.909 |
| sp|Q6ZQ38|CAND1_MOUSE | Cullin-associated NEDD8-dissociated protein 1 OS=Mus musculus GN=Cand1 PE=2 SV=2 | 214 | 1.027 | 0.968 | 0.924 |
| sp|O35639|ANXA3_MOUSE | Annexin A3 OS=Mus musculus GN=Anxa3 PE=1 SV=4 | 213 | 1.051 | 1.004 | 0.784 |
| sp|P09103|PDIA1_MOUSE | Protein disulfide-isomerase OS=Mus musculus GN=P4hb PE=1 SV=2 | 212 | 1.078 | 0.981 | 0.828 |
| sp|P61979|HNRPK_MOUSE | Heterogeneous nuclear ribonucleoprotein K OS=Mus musculus GN=Hnrnpk PE=1 SV=1 | 210 | 1.037 | 0.894 | 0.884 |
| sp|P26041|MOES_MOUSE | Moesin OS=Mus musculus GN=Msn PE=1 SV=3 | 209 | 1.286 | 1.190 | 1.317 |
| sp|P14602|HSPB1_MOUSE | Heat shock protein beta-1 OS=Mus musculus GN=Hspb1 PE=1 SV=3 | 204 | 0.919 | 0.686 | 0.609 |
| sp|O88569|ROA2_MOUSE | Heterogeneous nuclear ribonucleoproteins A2/B1 OS=Mus musculus GN=Hnrnpa2b1 PE=1 SV=2 | 201 | 0.922 | 1.301 | 0.830 |
| sp|P50516|VATA_MOUSE | V-type proton ATPase catalytic subunit A OS=Mus musculus GN=Atp6v1a PE=1 SV=2 | 197 | 1.099 | 1.019 | 1.014 |
| sp|P68368|TBA4A_MOUSE | Tubulin alpha-4A chain OS=Mus musculus GN=Tuba4a PE=1 SV=1 | 196 | 1.139 | 1.017 | 0.000 |
| sp|P26040|EZRI_MOUSE | Ezrin OS=Mus musculus GN=Ezr PE=1 SV=3 | 196 | 1.000 | 0.895 | 0.837 |
| sp|Q61316|HSP74_MOUSE | Heat shock 70 kDa protein 4 OS=Mus musculus GN=Hspa4 PE=1 SV=1 | 196 | 1.094 | 1.033 | 0.938 |
| sp|Q8BGZ7|K2C75_MOUSE | Keratin, type II cytoskeletal 75 OS=Mus musculus GN=Krt75 PE=1 SV=1 | 196 | 2.308 | 0.935 | 0.989 |
| sp|Q9ERD7|TBB3_MOUSE | Tubulin beta-3 chain OS=Mus musculus GN=Tubb3 PE=1 SV=1 | 193 | 1.170 | 0.983 | 0.915 |
| sp|E9Q557|DESP_MOUSE | Desmoplakin OS=Mus musculus GN=Dsp PE=3 SV=1 | 192 | 1.186 | 0.706 | 0.528 |
| sp|P62806|H4_MOUSE | Histone H4 OS=Mus musculus GN=Hist1h4a PE=1 SV=2 | 190 | 0.882 | 1.085 | 0.928 |
| sp|P16858|G3P_MOUSE | Glyceraldehyde-3-phosphate dehydrogenase OS=Mus musculus GN=Gapdh PE=1 SV=2 | 190 | 0.979 | 0.878 | 0.966 |
| sp|P17879|HS71B_MOUSE | Heat shock 70 kDa protein 1B OS=Mus musculus GN=Hspa1b PE=1 SV=3 | 188 | 1.184 | 1.031 | 0.897 |
| sp|Q61696|HS71A_MOUSE | Heat shock 70 kDa protein 1A OS=Mus musculus GN=Hspa1a PE=1 SV=2 | 188 | 1.164 | 1.019 | 0.898 |
| sp|P29341|PABP1_MOUSE | Polyadenylate-binding protein 1 OS=Mus musculus GN=Pabpc1 PE=1 SV=2 | 187 | 1.152 | 0.869 | 0.701 |
| sp|P17742|PPIA_MOUSE | Peptidyl-prolyl cis-trans isomerase A OS=Mus musculus GN=Ppia PE=1 SV=2 | 182 | 1.031 | 0.932 | 0.845 |
| sp|Q99KI0|ACON_MOUSE | Aconitate hydratase, mitochondrial OS=Mus musculus GN=Aco2 PE=1 SV=1 | 182 | 1.072 | 0.885 | 0.859 |
| sp|P35564|CALX_MOUSE | Calnexin OS=Mus musculus GN=Canx PE=1 SV=1 | 178 | 1.158 | 1.127 | 1.009 |
| sp|Q9Z1Q9|SYVC_MOUSE | Valine--tRNA ligase OS=Mus musculus GN=Vars PE=2 SV=1 | 176 | 1.365 | 1.312 | 1.295 |
| sp|O70133|DHX9_MOUSE | ATP-dependent RNA helicase A OS=Mus musculus GN=Dhx9 PE=1 SV=2 | 175 | 1.032 | 0.867 | 0.786 |
| sp|Q9CY58|PAIRB_MOUSE | Plasminogen activator inhibitor 1 RNA-binding protein OS=Mus musculus GN=Serbp1 PE=1 SV=2 | 174 | 1.087 | 0.975 | 0.908 |
| sp|Q8CIE6|COPA_MOUSE | Coatomer subunit alpha OS=Mus musculus GN=Copa PE=1 SV=2 | 172 | 1.095 | 0.970 | 0.917 |
| sp|Q8BMS1|ECHA_MOUSE | Trifunctional enzyme subunit alpha, mitochondrial OS=Mus musculus GN=Hadha PE=1 SV=1 | 171 | 1.034 | 0.891 | 0.731 |
| sp|Q8BMK4|CKAP4_MOUSE | Cytoskeleton-associated protein 4 OS=Mus musculus GN=Ckap4 PE=2 SV=2 | 170 | 1.145 | 1.011 | 0.832 |
| sp|P70168|IMB1_MOUSE | Importin subunit beta-1 OS=Mus musculus GN=Kpnb1 PE=1 SV=2 | 170 | 1.058 | 0.955 | 0.904 |
| sp|P80314|TCPB_MOUSE | T-complex protein 1 subunit beta OS=Mus musculus GN=Cct2 PE=1 SV=4 | 170 | 1.115 | 1.053 | 0.906 |
| sp|Q61414|K1C15_MOUSE | Keratin, type I cytoskeletal 15 OS=Mus musculus GN=Krt15 PE=1 SV=2 | 169 | 2.000 | 1.000 | 0.797 |
| sp|Q99KK7|DPP3_MOUSE | Dipeptidyl peptidase 3 OS=Mus musculus GN=Dpp3 PE=2 SV=2 | 168 | 1.141 | 1.051 | 0.965 |
| sp|Q8CAQ8|IMMT_MOUSE | Mitochondrial inner membrane protein OS=Mus musculus GN=Immt PE=1 SV=1 | 166 | 1.050 | 1.064 | 0.771 |
| sp|Q61768|KINH_MOUSE | Kinesin-1 heavy chain OS=Mus musculus GN=Kif5b PE=1 SV=3 | 166 | 1.068 | 0.746 | 0.844 |
| sp|P13020|GELS_MOUSE | Gelsolin OS=Mus musculus GN=Gsn PE=1 SV=3 | 165 | 0.898 | 0.731 | 0.559 |
| sp|P06151|LDHA_MOUSE | L-lactate dehydrogenase A chain OS=Mus musculus GN=Ldha PE=1 SV=3 | 165 | 1.118 | 0.962 | 0.877 |
| sp|Q61595|KTN1_MOUSE | Kinectin OS=Mus musculus GN=Ktn1 PE=1 SV=1 | 162 | 0.845 | 0.911 | 0.665 |
| sp|O35350|CAN1_MOUSE | Calpain-1 catalytic subunit OS=Mus musculus GN=Capn1 PE=2 SV=1 | 162 | 1.011 | 0.740 | 0.701 |
| sp|P16627|HS71L_MOUSE | Heat shock 70 kDa protein 1-like OS=Mus musculus GN=Hspa1l PE=2 SV=4 | 161 | 1.136 | 0.979 | 0.870 |
| sp|Q9D8N0|EF1G_MOUSE | Elongation factor 1-gamma OS=Mus musculus GN=Eef1g PE=1 SV=3 | 161 | 1.132 | 1.024 | 0.913 |
| sp|Q78PY7|SND1_MOUSE | Staphylococcal nuclease domain-containing protein 1 OS=Mus musculus GN=Snd1 PE=1 SV=1 | 160 | 1.268 | 1.010 | 0.791 |
| sp|Q62167|DDX3X_MOUSE | ATP-dependent RNA helicase DDX3X OS=Mus musculus GN=Ddx3x PE=1 SV=3 | 160 | 1.070 | 0.912 | 0.807 |
| sp|Q60932|VDAC1_MOUSE | Voltage-dependent anion-selective channel protein 1 OS=Mus musculus GN=Vdac1 PE=1 SV=3 | 158 | 0.932 | 0.905 | 0.819 |
| sp|P60843|IF4A1_MOUSE | Eukaryotic initiation factor 4A-I OS=Mus musculus GN=Eif4a1 PE=2 SV=1 | 158 | 1.150 | 1.026 | 0.923 |
| sp|P26350|PTMA_MOUSE | Prothymosin alpha OS=Mus musculus GN=Ptma PE=1 SV=2 | 157 | 1.086 | 1.044 | 1.054 |
| sp|Q64727|VINC_MOUSE | Vinculin OS=Mus musculus GN=Vcl PE=1 SV=4 | 156 | 1.064 | 0.921 | 0.860 |
| sp|O70456|1433S_MOUSE | 14-3-3 protein sigma OS=Mus musculus GN=Sfn PE=1 SV=2 | 155 | 0.984 | 0.850 | 0.727 |
| sp|Q6NZJ6|IF4G1_MOUSE | Eukaryotic translation initiation factor 4 gamma 1 OS=Mus musculus GN=Eif4g1 PE=1 SV=1 | 154 | 1.070 | 1.054 | 0.755 |
| sp|P27546|MAP4_MOUSE | Microtubule-associated protein 4 OS=Mus musculus GN=Map4 PE=1 SV=3 | 153 | 1.158 | 1.119 | 0.791 |
| sp|Q99NB9|SF3B1_MOUSE | Splicing factor 3B subunit 1 OS=Mus musculus GN=Sf3b1 PE=1 SV=1 | 153 | 1.066 | 1.000 | 0.980 |
| sp|P62270|RS18_MOUSE | 40S ribosomal protein S18 OS=Mus musculus GN=Rps18 PE=1 SV=3 | 152 | 1.146 | 1.000 | 0.923 |
| sp|Q9JKR6|HYOU1_MOUSE | Hypoxia up-regulated protein 1 OS=Mus musculus GN=Hyou1 PE=1 SV=1 | 147 | 1.198 | 0.993 | 0.902 |
| sp|O54988|SLK_MOUSE | STE20-like serine/threonine-protein kinase OS=Mus musculus GN=Slk PE=1 SV=2 | 146 | 1.029 | 0.929 | 0.784 |
| sp|P99027|RLA2_MOUSE | 60S acidic ribosomal protein P2 OS=Mus musculus GN=Rplp2 PE=1 SV=3 | 146 | 1.061 | 0.954 | 0.902 |
| sp|P70124|SPB5_MOUSE | Serpin B5 OS=Mus musculus GN=Serpinb5 PE=2 SV=1 | 146 | 0.993 | 0.972 | 0.920 |
| sp|P06745|G6PI_MOUSE | Glucose-6-phosphate isomerase OS=Mus musculus GN=Gpi PE=1 SV=4 | 145 | 1.029 | 0.892 | 0.738 |
| sp|Q8VEM8|MPCP_MOUSE | Phosphate carrier protein, mitochondrial OS=Mus musculus GN=Slc25a3 PE=1 SV=1 | 145 | 1.088 | 0.859 | 0.764 |
| sp|Q9EPU0|RENT1_MOUSE | Regulator of nonsense transcripts 1 OS=Mus musculus GN=Upf1 PE=1 SV=2 | 145 | 1.000 | 0.945 | 0.789 |
| sp|P26043|RADI_MOUSE | Radixin OS=Mus musculus GN=Rdx PE=1 SV=3 | 144 | 0.959 | 1.094 | 1.073 |
| sp|O08709|PRDX6_MOUSE | Peroxiredoxin-6 OS=Mus musculus GN=Prdx6 PE=1 SV=3 | 142 | 0.933 | 0.827 | 0.674 |
| sp|P47739|AL3A1_MOUSE | Aldehyde dehydrogenase, dimeric NADP-preferring OS=Mus musculus GN=Aldh3a1 PE=2 SV=2 | 141 | 0.921 | 0.837 | 0.591 |
| sp|Q9DBJ1|PGAM1_MOUSE | Phosphoglycerate mutase 1 OS=Mus musculus GN=Pgam1 PE=1 SV=3 | 140 | 1.141 | 1.071 | 0.965 |
| sp|Q9CZD3|SYG_MOUSE | Glycine--tRNA ligase OS=Mus musculus GN=Gars PE=1 SV=1 | 137 | 1.111 | 1.000 | 0.921 |
| sp|Q9EQH3|VPS35_MOUSE | Vacuolar protein sorting-associated protein 35 OS=Mus musculus GN=Vps35 PE=1 SV=1 | 136 | 1.118 | 0.995 | 0.937 |
| sp|Q64523|H2A2C_MOUSE | Histone H2A type 2-C OS=Mus musculus GN=Hist2h2ac PE=1 SV=3 | 133 | 0.904 | 0.911 | 0.862 |
| sp|Q6GSS7|H2A2A_MOUSE | Histone H2A type 2-A OS=Mus musculus GN=Hist2h2aa1 PE=1 SV=3 | 133 | 0.875 | 0.898 | 0.896 |
| sp|O08788|DCTN1_MOUSE | Dynactin subunit 1 OS=Mus musculus GN=Dctn1 PE=1 SV=3 | 132 | 0.986 | 0.945 | 0.806 |
| sp|Q9Z0U1|ZO2_MOUSE | Tight junction protein ZO-2 OS=Mus musculus GN=Tjp2 PE=1 SV=2 | 132 | 1.170 | 1.254 | 0.909 |
| sp|Q9Z2X1|HNRPF_MOUSE | Heterogeneous nuclear ribonucleoprotein F OS=Mus musculus GN=Hnrnpf PE=1 SV=3 | 132 | 1.119 | 0.958 | 0.937 |
| sp|P10630|IF4A2_MOUSE | Eukaryotic initiation factor 4A-II OS=Mus musculus GN=Eif4a2 PE=2 SV=2 | 131 | 1.173 | 1.000 | 0.889 |
| sp|P14733|LMNB1_MOUSE | Lamin-B1 OS=Mus musculus GN=Lmnb1 PE=1 SV=3 | 129 | 0.946 | 0.945 | 0.790 |
| sp|P48036|ANXA5_MOUSE | Annexin A5 OS=Mus musculus GN=Anxa5 PE=1 SV=1 | 128 | 1.056 | 1.125 | 0.705 |
| sp|Q9D379|HYEP_MOUSE | Epoxide hydrolase 1 OS=Mus musculus GN=Ephx1 PE=1 SV=2 | 128 | 1.222 | 1.054 | 0.920 |
| sp|P62259|1433E_MOUSE | 14-3-3 protein epsilon OS=Mus musculus GN=Ywhae PE=1 SV=1 | 128 | 1.084 | 0.969 | 0.946 |
| sp|Q8R1F1|NIBL1_MOUSE | Niban-like protein 1 OS=Mus musculus GN=Fam129b PE=1 SV=2 | 127 | 1.044 | 0.907 | 0.850 |
| sp|Q61171|PRDX2_MOUSE | Peroxiredoxin-2 OS=Mus musculus GN=Prdx2 PE=1 SV=3 | 127 | 1.017 | 0.969 | 1.020 |
| sp|P08249|MDHM_MOUSE | Malate dehydrogenase, mitochondrial OS=Mus musculus GN=Mdh2 PE=1 SV=3 | 126 | 1.085 | 0.927 | 0.811 |
| sp|P50544|ACADV_MOUSE | Very long-chain specific acyl-CoA dehydrogenase, mitochondrial OS=Mus musculus GN=Acadvl PE=1 SV=3 | 126 | 1.010 | 0.897 | 0.914 |
| sp|P12970|RL7A_MOUSE | 60S ribosomal protein L7a OS=Mus musculus GN=Rpl7a PE=2 SV=2 | 124 | 1.077 | 0.945 | 0.907 |
| sp|Q922F4|TBB6_MOUSE | Tubulin beta-6 chain OS=Mus musculus GN=Tubb6 PE=1 SV=1 | 122 | 1.167 | InD | 0.000 |
| sp|Q60597|ODO1_MOUSE | 2-oxoglutarate dehydrogenase, mitochondrial OS=Mus musculus GN=Ogdh PE=1 SV=3 | 122 | 1.121 | 0.885 | 0.893 |
| sp|Q8C1A5|THOP1_MOUSE | Thimet oligopeptidase OS=Mus musculus GN=Thop1 PE=1 SV=1 | 122 | 0.973 | 0.927 | 0.909 |
| sp|P80317|TCPZ_MOUSE | T-complex protein 1 subunit zeta OS=Mus musculus GN=Cct6a PE=1 SV=3 | 121 | 1.122 | 1.020 | 0.960 |
| sp|P42932|TCPQ_MOUSE | T-complex protein 1 subunit theta OS=Mus musculus GN=Cct8 PE=1 SV=3 | 120 | 1.171 | 1.073 | 0.968 |
| sp|Q11011|PSA_MOUSE | Puromycin-sensitive aminopeptidase OS=Mus musculus GN=Npepps PE=1 SV=2 | 120 | 1.307 | 1.006 | 1.104 |
| sp|Q64514|TPP2_MOUSE | Tripeptidyl-peptidase 2 OS=Mus musculus GN=Tpp2 PE=2 SV=3 | 119 | 1.168 | 1.009 | 0.953 |
| sp|Q8VEK3|HNRPU_MOUSE | Heterogeneous nuclear ribonucleoprotein U OS=Mus musculus GN=Hnrnpu PE=1 SV=1 | 119 | 1.044 | 0.979 | 0.963 |
| sp|Q9QUR6|PPCE_MOUSE | Prolyl endopeptidase OS=Mus musculus GN=Prep PE=2 SV=1 | 119 | 1.119 | 1.037 | 0.982 |
| sp|Q6IFX2|K1C42_MOUSE | Keratin, type I cytoskeletal 42 OS=Mus musculus GN=Krt42 PE=1 SV=1 | 118 | 1.817 | 1.024 | InD |
| sp|P62908|RS3_MOUSE | 40S ribosomal protein S3 OS=Mus musculus GN=Rps3 PE=1 SV=1 | 117 | 1.059 | 0.957 | 0.871 |
| sp|P50247|SAHH_MOUSE | Adenosylhomocysteinase OS=Mus musculus GN=Ahcy PE=1 SV=3 | 117 | 1.015 | 0.882 | 0.910 |
| sp|P35980|RL18_MOUSE | 60S ribosomal protein L18 OS=Mus musculus GN=Rpl18 PE=2 SV=3 | 116 | 1.116 | 0.952 | 0.922 |
| sp|Q9D0R2|SYTC_MOUSE | Threonine--tRNA ligase, cytoplasmic OS=Mus musculus GN=Tars PE=1 SV=2 | 115 | 1.081 | 0.941 | 0.905 |
| sp|Q3UPL0|SC31A_MOUSE | Protein transport protein Sec31A OS=Mus musculus GN=Sec31a PE=1 SV=2 | 115 | 1.139 | 0.980 | 0.946 |
| sp|Q64521|GPDM_MOUSE | Glycerol-3-phosphate dehydrogenase, mitochondrial OS=Mus musculus GN=Gpd2 PE=1 SV=2 | 114 | 1.054 | 0.868 | 0.801 |
| sp|Q8VDN2|AT1A1_MOUSE | Sodium/potassium-transporting ATPase subunit alpha-1 OS=Mus musculus GN=Atp1a1 PE=1 SV=1 | 114 | 1.067 | 0.918 | 0.806 |
| sp|P51150|RAB7A_MOUSE | Ras-related protein Rab-7a OS=Mus musculus GN=Rab7a PE=1 SV=2 | 114 | 1.000 | 0.980 | 0.898 |
| sp|P27659|RL3_MOUSE | 60S ribosomal protein L3 OS=Mus musculus GN=Rpl3 PE=2 SV=3 | 114 | 1.101 | 0.953 | 0.900 |
| sp|Q8VDM4|PSMD2_MOUSE | 26S proteasome non-ATPase regulatory subunit 2 OS=Mus musculus GN=Psmd2 PE=1 SV=1 | 114 | 1.174 | 0.976 | 0.983 |
| sp|Q61598|GDIB_MOUSE | Rab GDP dissociation inhibitor beta OS=Mus musculus GN=Gdi2 PE=1 SV=1 | 113 | 1.045 | 1.007 | 0.834 |
| sp|Q8VIJ6|SFPQ_MOUSE | Splicing factor, proline- and glutamine-rich OS=Mus musculus GN=Sfpq PE=1 SV=1 | 113 | 1.000 | 0.856 | 0.930 |
| sp|Q8BH59|CMC1_MOUSE | Calcium-binding mitochondrial carrier protein Aralar1 OS=Mus musculus GN=Slc25a12 PE=1 SV=1 | 112 | 0.986 | 0.897 | 0.789 |
| sp|P05064|ALDOA_MOUSE | Fructose-bisphosphate aldolase A OS=Mus musculus GN=Aldoa PE=1 SV=2 | 112 | 1.139 | 0.968 | 0.923 |
| sp|Q9JIK5|DDX21_MOUSE | Nucleolar RNA helicase 2 OS=Mus musculus GN=Ddx21 PE=1 SV=3 | 112 | 1.042 | 1.256 | 0.973 |
| sp|O35129|PHB2_MOUSE | Prohibitin-2 OS=Mus musculus GN=Phb2 PE=1 SV=1 | 111 | 1.049 | 0.880 | 0.899 |
| sp|Q61029|LAP2B_MOUSE | Lamina-associated polypeptide 2, isoforms beta/delta/epsilon/gamma OS=Mus musculus GN=Tmpo PE=1 SV=4 | 110 | 0.972 | 0.915 | 0.745 |
| sp|P08003|PDIA4_MOUSE | Protein disulfide-isomerase A4 OS=Mus musculus GN=Pdia4 PE=1 SV=3 | 110 | 1.190 | 1.026 | 0.873 |
| sp|P08730|K1C13_MOUSE | Keratin, type I cytoskeletal 13 OS=Mus musculus GN=Krt13 PE=1 SV=2 | 110 | 1.560 | 1.370 | 0.875 |
| sp|P11983|TCPA_MOUSE | T-complex protein 1 subunit alpha OS=Mus musculus GN=Tcp1 PE=1 SV=3 | 110 | 1.207 | 1.031 | 0.947 |
| sp|Q9WVA4|TAGL2_MOUSE | Transgelin-2 OS=Mus musculus GN=Tagln2 PE=1 SV=4 | 109 | 1.333 | 1.238 | 1.385 |
| sp|P14152|MDHC_MOUSE | Malate dehydrogenase, cytoplasmic OS=Mus musculus GN=Mdh1 PE=1 SV=3 | 108 | 0.984 | 0.883 | 0.758 |
| sp|Q8BMJ2|SYLC_MOUSE | Leucine--tRNA ligase, cytoplasmic OS=Mus musculus GN=Lars PE=2 SV=2 | 108 | 1.194 | 1.000 | 0.986 |
| sp|Q6IRU2|TPM4_MOUSE | Tropomyosin alpha-4 chain OS=Mus musculus GN=Tpm4 PE=2 SV=3 | 107 | 1.175 | 1.217 | 1.193 |
| sp|Q9WU78|PDC6I_MOUSE | Programmed cell death 6-interacting protein OS=Mus musculus GN=Pdcd6ip PE=1 SV=3 | 106 | 0.951 | 1.173 | 0.724 |
| sp|Q8K310|MATR3_MOUSE | Matrin-3 OS=Mus musculus GN=Matr3 PE=1 SV=1 | 106 | 1.252 | 1.051 | 0.939 |
| sp|P08228|SODC_MOUSE | Superoxide dismutase [Cu-Zn] OS=Mus musculus GN=Sod1 PE=1 SV=2 | 106 | 1.005 | 0.920 | 0.972 |
| sp|Q9Z2K1|K1C16_MOUSE | Keratin, type I cytoskeletal 16 OS=Mus musculus GN=Krt16 PE=1 SV=3 | 106 | 2.413 | 1.250 | InD |
| sp|O35640|ANXA8_MOUSE | Annexin A8 OS=Mus musculus GN=Anxa8 PE=2 SV=2 | 105 | 0.850 | 0.593 | 0.615 |
| sp|O35737|HNRH1_MOUSE | Heterogeneous nuclear ribonucleoprotein H OS=Mus musculus GN=Hnrnph1 PE=1 SV=3 | 105 | 1.198 | 1.000 | 0.901 |
| sp|P24527|LKHA4_MOUSE | Leukotriene A-4 hydrolase OS=Mus musculus GN=Lta4h PE=1 SV=4 | 104 | 0.979 | 0.922 | 0.935 |
| sp|P26443|DHE3_MOUSE | Glutamate dehydrogenase 1, mitochondrial OS=Mus musculus GN=Glud1 PE=1 SV=1 | 103 | 1.119 | 0.968 | 0.819 |
| sp|Q8BFZ3|ACTBL_MOUSE | Beta-actin-like protein 2 OS=Mus musculus GN=Actbl2 PE=1 SV=1 | 102 | 1.041 | 0.927 | 0.801 |
| sp|Q9DCD0|6PGD_MOUSE | 6-phosphogluconate dehydrogenase, decarboxylating OS=Mus musculus GN=Pgd PE=2 SV=3 | 102 | 1.029 | 1.066 | 0.873 |
| sp|P62082|RS7_MOUSE | 40S ribosomal protein S7 OS=Mus musculus GN=Rps7 PE=2 SV=1 | 102 | 1.193 | 1.000 | 0.978 |
| sp|Q9CWJ9|PUR9_MOUSE | Bifunctional purine biosynthesis protein PURH OS=Mus musculus GN=Atic PE=1 SV=2 | 102 | 1.232 | 1.084 | 1.151 |
| sp|Q60864|STIP1_MOUSE | Stress-induced-phosphoprotein 1 OS=Mus musculus GN=Stip1 PE=1 SV=1 | 101 | 1.093 | 1.099 | 0.954 |
| sp|Q9QXZ0|MACF1_MOUSE | Microtubule-actin cross-linking factor 1 OS=Mus musculus GN=Macf1 PE=1 SV=2 | 101 | 1.338 | 1.246 | 1.015 |
| sp|Q9D0I9|SYRC_MOUSE | Arginine--tRNA ligase, cytoplasmic OS=Mus musculus GN=Rars PE=2 SV=2 | 100 | 1.151 | 0.864 | 0.905 |
| sp|P80313|TCPH_MOUSE | T-complex protein 1 subunit eta OS=Mus musculus GN=Cct7 PE=1 SV=1 | 100 | 1.108 | 1.020 | 0.990 |
| sp|P63242|IF5A1_MOUSE | Eukaryotic translation initiation factor 5A-1 OS=Mus musculus GN=Eif5a PE=1 SV=2 | 100 | 1.156 | 1.070 | 1.038 |
| sp|P24270|CATA_MOUSE | Catalase OS=Mus musculus GN=Cat PE=1 SV=4 | 100 | 1.194 | 1.107 | 1.731 |
| sp|P05202|AATM_MOUSE | Aspartate aminotransferase, mitochondrial OS=Mus musculus GN=Got2 PE=1 SV=1 | 99 | 1.007 | 0.937 | 0.923 |
| sp|Q9D952|EVPL_MOUSE | Envoplakin OS=Mus musculus GN=Evpl PE=2 SV=3 | 98 | 0.677 | 0.846 | 0.440 |
| sp|Q9CZU6|CISY_MOUSE | Citrate synthase, mitochondrial OS=Mus musculus GN=Cs PE=1 SV=1 | 98 | 1.115 | 0.888 | 0.827 |
| sp|P62814|VATB2_MOUSE | V-type proton ATPase subunit B, brain isoform OS=Mus musculus GN=Atp6v1b2 PE=1 SV=1 | 98 | 1.125 | 1.031 | 0.990 |
| sp|P18760|COF1_MOUSE | Cofilin-1 OS=Mus musculus GN=Cfl1 PE=1 SV=3 | 98 | 1.237 | 1.196 | 1.124 |
| sp|P22752|H2A1_MOUSE | Histone H2A type 1 OS=Mus musculus GN=Hist1h2ab PE=1 SV=3 | 97 | InD | 0.918 | 0.865 |
| sp|Q8CGP5|H2A1F_MOUSE | Histone H2A type 1-F OS=Mus musculus GN=Hist1h2af PE=1 SV=3 | 97 | InD | 0.918 | 0.865 |
| sp|Q8CGP6|H2A1H_MOUSE | Histone H2A type 1-H OS=Mus musculus GN=Hist1h2ah PE=1 SV=3 | 97 | InD | 0.918 | 0.865 |
| sp|Q8CGP7|H2A1K_MOUSE | Histone H2A type 1-K OS=Mus musculus GN=Hist1h2ak PE=1 SV=3 | 97 | InD | 0.918 | 0.865 |
| sp|Q8R1M2|H2AJ_MOUSE | Histone H2A.J OS=Mus musculus GN=H2afj PE=2 SV=1 | 97 | InD | 0.918 | 0.865 |
| sp|Q8VDJ3|VIGLN_MOUSE | Vigilin OS=Mus musculus GN=Hdlbp PE=1 SV=1 | 97 | 1.195 | 1.082 | 0.897 |
| sp|Q8BFU2|H2A3_MOUSE | Histone H2A type 3 OS=Mus musculus GN=Hist3h2a PE=1 SV=3 | 97 | InD | 0.924 | 0.897 |
| sp|P19157|GSTP1_MOUSE | Glutathione S-transferase P 1 OS=Mus musculus GN=Gstp1 PE=1 SV=2 | 97 | 1.182 | 1.062 | 0.935 |
| sp|P62702|RS4X_MOUSE | 40S ribosomal protein S4, X isoform OS=Mus musculus GN=Rps4x PE=2 SV=2 | 96 | 1.173 | 0.988 | 0.910 |
| sp|P80318|TCPG_MOUSE | T-complex protein 1 subunit gamma OS=Mus musculus GN=Cct3 PE=1 SV=1 | 96 | 1.211 | 1.084 | 0.969 |
| sp|Q8BG05|ROA3_MOUSE | Heterogeneous nuclear ribonucleoprotein A3 OS=Mus musculus GN=Hnrnpa3 PE=1 SV=1 | 95 | 0.849 | 1.159 | 1.017 |
| sp|Q8BU30|SYIC_MOUSE | Isoleucine--tRNA ligase, cytoplasmic OS=Mus musculus GN=Iars PE=2 SV=2 | 95 | 1.136 | 0.938 | 1.032 |
| sp|Q61033|LAP2A_MOUSE | Lamina-associated polypeptide 2, isoforms alpha/zeta OS=Mus musculus GN=Tmpo PE=1 SV=4 | 94 | 1.022 | 0.908 | 0.857 |
| sp|Q7TMK9|HNRPQ_MOUSE | Heterogeneous nuclear ribonucleoprotein Q OS=Mus musculus GN=Syncrip PE=1 SV=2 | 94 | 1.050 | 0.915 | 0.898 |
| sp|P15532|NDKA_MOUSE | Nucleoside diphosphate kinase A OS=Mus musculus GN=Nme1 PE=1 SV=1 | 94 | 1.107 | 1.065 | 0.965 |
| sp|P14824|ANXA6_MOUSE | Annexin A6 OS=Mus musculus GN=Anxa6 PE=1 SV=3 | 93 | 0.800 | 1.000 | 0.756 |
| sp|Q8BG32|PSD11_MOUSE | 26S proteasome non-ATPase regulatory subunit 11 OS=Mus musculus GN=Psmd11 PE=1 SV=3 | 93 | 1.095 | 0.939 | 0.957 |
| sp|Q9DBS1|TMM43_MOUSE | Transmembrane protein 43 OS=Mus musculus GN=Tmem43 PE=1 SV=1 | 92 | 0.995 | 0.863 | 0.818 |
| sp|P67778|PHB_MOUSE | Prohibitin OS=Mus musculus GN=Phb PE=1 SV=1 | 92 | 1.101 | 0.860 | 0.861 |
| sp|P62827|RAN_MOUSE | GTP-binding nuclear protein Ran OS=Mus musculus GN=Ran PE=1 SV=3 | 92 | 1.027 | 1.029 | 0.932 |
| sp|Q5SUR0|PUR4_MOUSE | Phosphoribosylformylglycinamidine synthase OS=Mus musculus GN=Pfas PE=2 SV=1 | 92 | 1.034 | 1.074 | 1.045 |
| sp|Q8R1B4|EIF3C_MOUSE | Eukaryotic translation initiation factor 3 subunit C OS=Mus musculus GN=Eif3c PE=1 SV=1 | 92 | 1.108 | 1.077 | 1.063 |
| sp|Q9ES97|RTN3_MOUSE | Reticulon-3 OS=Mus musculus GN=Rtn3 PE=1 SV=2 | 91 | 1.050 | 0.961 | 0.835 |
| sp|Q9D0E1|HNRPM_MOUSE | Heterogeneous nuclear ribonucleoprotein M OS=Mus musculus GN=Hnrnpm PE=1 SV=3 | 91 | 1.157 | 0.967 | 0.894 |
| sp|P68040|GBLP_MOUSE | Guanine nucleotide-binding protein subunit beta-2-like 1 OS=Mus musculus GN=Gnb2l1 PE=1 SV=3 | 91 | 1.147 | 0.926 | 0.921 |
| sp|Q9CPV4|GLOD4_MOUSE | Glyoxalase domain-containing protein 4 OS=Mus musculus GN=Glod4 PE=2 SV=1 | 91 | 1.051 | 0.933 | 0.926 |
| sp|Q91VC3|IF4A3_MOUSE | Eukaryotic initiation factor 4A-III OS=Mus musculus GN=Eif4a3 PE=2 SV=3 | 91 | 1.139 | 0.992 | 0.940 |
| sp|P70296|PEBP1_MOUSE | Phosphatidylethanolamine-binding protein 1 OS=Mus musculus GN=Pebp1 PE=1 SV=3 | 90 | 1.063 | 0.960 | 0.858 |
| sp|Q8BGQ7|SYAC_MOUSE | Alanine--tRNA ligase, cytoplasmic OS=Mus musculus GN=Aars PE=1 SV=1 | 90 | 1.174 | 0.915 | 0.864 |
| sp|P14211|CALR_MOUSE | Calreticulin OS=Mus musculus GN=Calr PE=1 SV=1 | 90 | 1.161 | 1.067 | 0.876 |
| sp|O35114|SCRB2_MOUSE | Lysosome membrane protein 2 OS=Mus musculus GN=Scarb2 PE=1 SV=3 | 90 | 0.938 | 0.881 | 0.910 |
| sp|P97429|ANXA4_MOUSE | Annexin A4 OS=Mus musculus GN=Anxa4 PE=2 SV=4 | 89 | 0.800 | 0.918 | 0.788 |
| sp|Q6P5E4|UGGG1_MOUSE | UDP-glucose:glycoprotein glucosyltransferase 1 OS=Mus musculus GN=Uggt1 PE=1 SV=4 | 89 | 0.971 | 1.007 | 0.789 |
| sp|Q6P1B1|XPP1_MOUSE | Xaa-Pro aminopeptidase 1 OS=Mus musculus GN=Xpnpep1 PE=2 SV=1 | 89 | 1.063 | 1.006 | 0.839 |
| sp|Q8K2B3|DHSA_MOUSE | Succinate dehydrogenase [ubiquinone] flavoprotein subunit, mitochondrial OS=Mus musculus GN=Sdha PE=1 SV=1 | 89 | 1.000 | 0.965 | 0.845 |
| sp|Q61656|DDX5_MOUSE | Probable ATP-dependent RNA helicase DDX5 OS=Mus musculus GN=Ddx5 PE=1 SV=2 | 89 | 1.088 | 0.966 | 1.012 |
| sp|Q91YQ5|RPN1_MOUSE | Dolichyl-diphosphooligosaccharide--protein glycosyltransferase subunit 1 OS=Mus musculus GN=Rpn1 PE=2 SV=1 | 88 | 1.062 | 1.012 | 0.901 |
| sp|P14869|RLA0_MOUSE | 60S acidic ribosomal protein P0 OS=Mus musculus GN=Rplp0 PE=1 SV=3 | 88 | 1.022 | 0.990 | 0.949 |
| sp|Q9QUM9|PSA6_MOUSE | Proteasome subunit alpha type-6 OS=Mus musculus GN=Psma6 PE=1 SV=1 | 88 | 1.053 | 1.044 | 0.984 |
| sp|Q3THK7|GUAA_MOUSE | GMP synthase [glutamine-hydrolyzing] OS=Mus musculus GN=Gmps PE=1 SV=2 | 88 | 1.220 | 1.190 | 1.179 |
| sp|O08749|DLDH_MOUSE | Dihydrolipoyl dehydrogenase, mitochondrial OS=Mus musculus GN=Dld PE=1 SV=2 | 87 | 1.083 | 0.916 | 0.831 |
| sp|Q61937|NPM_MOUSE | Nucleophosmin OS=Mus musculus GN=Npm1 PE=1 SV=1 | 87 | 1.130 | 0.961 | 1.000 |
| sp|P37040|NCPR_MOUSE | NADPH--cytochrome P450 reductase OS=Mus musculus GN=Por PE=1 SV=2 | 86 | 0.842 | 0.832 | 0.517 |
| sp|P99029|PRDX5_MOUSE | Peroxiredoxin-5, mitochondrial OS=Mus musculus GN=Prdx5 PE=1 SV=2 | 86 | 0.894 | 0.826 | 0.736 |
| sp|Q01768|NDKB_MOUSE | Nucleoside diphosphate kinase B OS=Mus musculus GN=Nme2 PE=1 SV=1 | 86 | 1.095 | 0.953 | 0.990 |
| sp|P19001|K1C19_MOUSE | Keratin, type I cytoskeletal 19 OS=Mus musculus GN=Krt19 PE=1 SV=1 | 85 | 1.906 | 1.043 | 0.000 |
| sp|P15864|H12_MOUSE | Histone H1.2 OS=Mus musculus GN=Hist1h1c PE=1 SV=2 | 85 | 0.882 | 0.286 | 0.364 |
| sp|P43277|H13_MOUSE | Histone H1.3 OS=Mus musculus GN=Hist1h1d PE=1 SV=2 | 85 | 0.882 | 0.502 | 0.364 |
| sp|Q61739|ITA6_MOUSE | Integrin alpha-6 OS=Mus musculus GN=Itga6 PE=1 SV=3 | 85 | 1.067 | 0.629 | 0.613 |
| sp|Q9D0K2|SCOT1_MOUSE | Succinyl-CoA:3-ketoacid coenzyme A transferase 1, mitochondrial OS=Mus musculus GN=Oxct1 PE=1 SV=1 | 85 | 0.978 | 0.891 | 0.742 |
| sp|Q8BFR5|EFTU_MOUSE | Elongation factor Tu, mitochondrial OS=Mus musculus GN=Tufm PE=1 SV=1 | 85 | 1.253 | 1.044 | 0.796 |
| sp|P46638|RB11B_MOUSE | Ras-related protein Rab-11B OS=Mus musculus GN=Rab11b PE=1 SV=3 | 85 | 1.080 | 0.995 | 0.884 |
| sp|P50580|PA2G4_MOUSE | Proliferation-associated protein 2G4 OS=Mus musculus GN=Pa2g4 PE=1 SV=3 | 85 | 1.053 | 0.978 | 0.902 |
| sp|O35286|DHX15_MOUSE | Putative pre-mRNA-splicing factor ATP-dependent RNA helicase DHX15 OS=Mus musculus GN=Dhx15 PE=2 SV=2 | 85 | 1.039 | 1.020 | 0.981 |
| sp|Q6PB66|LPPRC_MOUSE | Leucine-rich PPR motif-containing protein, mitochondrial OS=Mus musculus GN=Lrpprc PE=1 SV=2 | 84 | 0.992 | 0.867 | 0.822 |
| sp|Q6ZWN5|RS9_MOUSE | 40S ribosomal protein S9 OS=Mus musculus GN=Rps9 PE=2 SV=3 | 84 | 0.949 | 1.000 | 0.889 |
| sp|O55234|PSB5_MOUSE | Proteasome subunit beta type-5 OS=Mus musculus GN=Psmb5 PE=1 SV=3 | 84 | 1.121 | 0.992 | 0.920 |
| sp|Q9Z0X1|AIFM1_MOUSE | Apoptosis-inducing factor 1, mitochondrial OS=Mus musculus GN=Aifm1 PE=1 SV=1 | 84 | 1.174 | 0.972 | 1.010 |
| sp|P40124|CAP1_MOUSE | Adenylyl cyclase-associated protein 1 OS=Mus musculus GN=Cap1 PE=1 SV=4 | 83 | 1.035 | 1.115 | 0.858 |
| sp|Q9JMH9|MY18A_MOUSE | Unconventional myosin-XVIIIa OS=Mus musculus GN=Myo18a PE=1 SV=2 | 83 | 1.147 | 1.006 | 1.025 |
| sp|Q9QZE5|COPG1_MOUSE | Coatomer subunit gamma-1 OS=Mus musculus GN=Copg1 PE=2 SV=1 | 82 | 1.037 | 1.068 | 0.857 |
| sp|Q76MZ3|2AAA_MOUSE | Serine/threonine-protein phosphatase 2A 65 kDa regulatory subunit A alpha isoform OS=Mus musculus GN=Ppp2r1a PE=1 SV=3 | 82 | 1.035 | 0.895 | 0.895 |
| sp|Q9D8E6|RL4_MOUSE | 60S ribosomal protein L4 OS=Mus musculus GN=Rpl4 PE=1 SV=3 | 82 | 1.167 | 0.954 | 0.938 |
| sp|Q8R016|BLMH_MOUSE | Bleomycin hydrolase OS=Mus musculus GN=Blmh PE=2 SV=1 | 82 | 1.084 | 1.028 | 1.051 |
| sp|P61205|ARF3_MOUSE | ADP-ribosylation factor 3 OS=Mus musculus GN=Arf3 PE=2 SV=2 | 81 | 1.013 | 1.013 | 0.821 |
| sp|P84078|ARF1_MOUSE | ADP-ribosylation factor 1 OS=Mus musculus GN=Arf1 PE=1 SV=2 | 81 | 1.013 | 1.013 | 0.825 |
| sp|Q8K1M6|DNM1L_MOUSE | Dynamin-1-like protein OS=Mus musculus GN=Dnm1l PE=1 SV=2 | 81 | 0.978 | 0.900 | 0.939 |
| sp|P46471|PRS7_MOUSE | 26S protease regulatory subunit 7 OS=Mus musculus GN=Psmc2 PE=1 SV=5 | 81 | 1.083 | 1.079 | 0.970 |
| sp|Q8R2Q0|TRI29_MOUSE | Tripartite motif-containing protein 29 OS=Mus musculus GN=Trim29 PE=1 SV=1 | 80 | 0.883 | 0.777 | 0.714 |
| sp|Q99JY9|ARP3_MOUSE | Actin-related protein 3 OS=Mus musculus GN=Actr3 PE=1 SV=3 | 80 | 1.144 | 0.895 | 0.876 |
| sp|Q8BKC5|IPO5_MOUSE | Importin-5 OS=Mus musculus GN=Ipo5 PE=1 SV=3 | 80 | 1.250 | 1.213 | 0.957 |
| sp|Q9CVB6|ARPC2_MOUSE | Actin-related protein 2/3 complex subunit 2 OS=Mus musculus GN=Arpc2 PE=1 SV=3 | 80 | 1.037 | 0.948 | 0.959 |
| sp|P17225|PTBP1_MOUSE | Polypyrimidine tract-binding protein 1 OS=Mus musculus GN=Ptbp1 PE=1 SV=2 | 79 | 1.069 | 0.785 | 0.785 |
| sp|Q9JIF7|COPB_MOUSE | Coatomer subunit beta OS=Mus musculus GN=Copb1 PE=1 SV=1 | 79 | 1.268 | 1.184 | 0.879 |
| sp|Q6P5F9|XPO1_MOUSE | Exportin-1 OS=Mus musculus GN=Xpo1 PE=1 SV=1 | 79 | 1.008 | 0.938 | 0.986 |
| sp|Q99K30|ES8L2_MOUSE | Epidermal growth factor receptor kinase substrate 8-like protein 2 OS=Mus musculus GN=Eps8l2 PE=1 SV=1 | 78 | 0.964 | 0.790 | 0.645 |
| sp|Q9D6Z1|NOP56_MOUSE | Nucleolar protein 56 OS=Mus musculus GN=Nop56 PE=1 SV=2 | 78 | 0.905 | 1.098 | 0.881 |
| sp|P97351|RS3A_MOUSE | 40S ribosomal protein S3a OS=Mus musculus GN=Rps3a PE=1 SV=3 | 78 | 1.110 | 0.952 | 0.949 |
| sp|O35643|AP1B1_MOUSE | AP-1 complex subunit beta-1 OS=Mus musculus GN=Ap1b1 PE=1 SV=2 | 77 | 1.029 | 0.952 | 0.832 |
| sp|P62301|RS13_MOUSE | 40S ribosomal protein S13 OS=Mus musculus GN=Rps13 PE=1 SV=2 | 77 | 1.090 | 1.065 | 0.870 |
| sp|Q3U0V1|FUBP2_MOUSE | Far upstream element-binding protein 2 OS=Mus musculus GN=Khsrp PE=1 SV=2 | 77 | 0.940 | 0.915 | 0.925 |
| sp|Q05920|PYC_MOUSE | Pyruvate carboxylase, mitochondrial OS=Mus musculus GN=Pc PE=1 SV=1 | 76 | 0.900 | 0.865 | 0.557 |
| sp|P62821|RAB1A_MOUSE | Ras-related protein Rab-1A OS=Mus musculus GN=Rab1A PE=1 SV=3 | 76 | 0.994 | 0.940 | 0.800 |
| sp|Q9CZX8|RS19_MOUSE | 40S ribosomal protein S19 OS=Mus musculus GN=Rps19 PE=1 SV=3 | 76 | 1.136 | 1.000 | 0.883 |
| sp|P80316|TCPE_MOUSE | T-complex protein 1 subunit epsilon OS=Mus musculus GN=Cct5 PE=1 SV=1 | 76 | 1.143 | 1.054 | 0.978 |
| sp|P51174|ACADL_MOUSE | Long-chain specific acyl-CoA dehydrogenase, mitochondrial OS=Mus musculus GN=Acadl PE=2 SV=2 | 75 | 0.973 | 0.912 | 0.698 |
| sp|Q62426|CYTB_MOUSE | Cystatin-B OS=Mus musculus GN=Cstb PE=1 SV=1 | 75 | 1.064 | 0.964 | 0.804 |
| sp|P84084|ARF5_MOUSE | ADP-ribosylation factor 5 OS=Mus musculus GN=Arf5 PE=2 SV=2 | 75 | 1.125 | 1.067 | 0.831 |
| sp|P62267|RS23_MOUSE | 40S ribosomal protein S23 OS=Mus musculus GN=Rps23 PE=2 SV=3 | 75 | 1.130 | 0.889 | 0.833 |
| sp|A2AN08|UBR4_MOUSE | E3 ubiquitin-protein ligase UBR4 OS=Mus musculus GN=Ubr4 PE=1 SV=1 | 75 | 1.011 | 0.963 | 0.852 |
| sp|P63325|RS10_MOUSE | 40S ribosomal protein S10 OS=Mus musculus GN=Rps10 PE=1 SV=1 | 75 | 1.098 | 0.890 | 0.901 |
| sp|P00493|HPRT_MOUSE | Hypoxanthine-guanine phosphoribosyltransferase OS=Mus musculus GN=Hprt1 PE=1 SV=3 | 75 | 1.051 | 0.940 | 0.908 |
| sp|Q8JZQ9|EIF3B_MOUSE | Eukaryotic translation initiation factor 3 subunit B OS=Mus musculus GN=Eif3b PE=1 SV=1 | 75 | 1.011 | 0.956 | 0.921 |
| sp|P54775|PRS6B_MOUSE | 26S protease regulatory subunit 6B OS=Mus musculus GN=Psmc4 PE=1 SV=2 | 75 | 1.107 | 1.063 | 0.993 |
| sp|P16110|LEG3_MOUSE | Galectin-3 OS=Mus musculus GN=Lgals3 PE=1 SV=3 | 74 | 0.980 | 0.804 | 0.500 |
| sp|Q6A0A9|F120A_MOUSE | Constitutive coactivator of PPAR-gamma-like protein 1 OS=Mus musculus GN=FAM120A PE=1 SV=2 | 74 | 1.000 | 0.914 | 0.758 |
| sp|Q9WTI7|MYO1C_MOUSE | Unconventional myosin-Ic OS=Mus musculus GN=Myo1c PE=1 SV=2 | 74 | 1.017 | 1.222 | 0.923 |
| sp|Q05D44|IF2P_MOUSE | Eukaryotic translation initiation factor 5B OS=Mus musculus GN=Eif5b PE=1 SV=2 | 74 | 1.191 | 1.042 | 0.956 |
| sp|P62204|CALM_MOUSE | Calmodulin OS=Mus musculus GN=Calm1 PE=1 SV=2 | 74 | 1.225 | 1.129 | 1.054 |
| sp|P15626|GSTM2_MOUSE | Glutathione S-transferase Mu 2 OS=Mus musculus GN=Gstm2 PE=1 SV=2 | 73 | 0.868 | 0.664 | 0.407 |
| sp|O35143|ATIF1_MOUSE | ATPase inhibitor, mitochondrial OS=Mus musculus GN=Atpif1 PE=2 SV=2 | 73 | 0.973 | 0.910 | 0.826 |
| sp|P10639|THIO_MOUSE | Thioredoxin OS=Mus musculus GN=Txn PE=1 SV=3 | 73 | 1.033 | 0.961 | 0.887 |
| sp|P53986|MOT1_MOUSE | Monocarboxylate transporter 1 OS=Mus musculus GN=Slc16a1 PE=1 SV=1 | 73 | 0.869 | 0.911 | 0.894 |
| sp|Q9Z0N1|IF2G_MOUSE | Eukaryotic translation initiation factor 2 subunit 3, X-linked OS=Mus musculus GN=Eif2s3x PE=1 SV=2 | 73 | 1.093 | 0.898 | 0.953 |
| sp|P43274|H14_MOUSE | Histone H1.4 OS=Mus musculus GN=Hist1h1e PE=1 SV=2 | 72 | 0.853 | 0.421 | 0.358 |
| sp|P68254|1433T_MOUSE | 14-3-3 protein theta OS=Mus musculus GN=Ywhaq PE=1 SV=1 | 72 | 0.902 | 0.825 | 0.806 |
| sp|P21107|TPM3_MOUSE | Tropomyosin alpha-3 chain OS=Mus musculus GN=Tpm3 PE=1 SV=2 | 72 | 1.048 | 0.939 | 0.814 |
| sp|P62962|PROF1_MOUSE | Profilin-1 OS=Mus musculus GN=Pfn1 PE=1 SV=2 | 72 | 1.123 | 1.059 | 0.886 |
| sp|Q9DB20|ATPO_MOUSE | ATP synthase subunit O, mitochondrial OS=Mus musculus GN=Atp5o PE=1 SV=1 | 72 | 1.018 | 0.966 | 0.893 |
| sp|P30416|FKBP4_MOUSE | Peptidyl-prolyl cis-trans isomerase FKBP4 OS=Mus musculus GN=Fkbp4 PE=1 SV=5 | 72 | 1.361 | 1.152 | 0.967 |
| sp|P80315|TCPD_MOUSE | T-complex protein 1 subunit delta OS=Mus musculus GN=Cct4 PE=1 SV=3 | 72 | 1.168 | 1.041 | 0.980 |
| sp|P12388|PAI2_MOUSE | Plasminogen activator inhibitor 2, macrophage OS=Mus musculus GN=Serpinb2 PE=2 SV=1 | 71 | 0.924 | 0.614 | 0.635 |
| sp|P56399|UBP5_MOUSE | Ubiquitin carboxyl-terminal hydrolase 5 OS=Mus musculus GN=Usp5 PE=1 SV=1 | 71 | 1.243 | 0.835 | 0.810 |
| sp|O54724|PTRF_MOUSE | Polymerase I and transcript release factor OS=Mus musculus GN=Ptrf PE=1 SV=1 | 71 | 1.036 | 0.929 | 0.889 |
| sp|P25444|RS2_MOUSE | 40S ribosomal protein S2 OS=Mus musculus GN=Rps2 PE=1 SV=3 | 71 | 1.074 | 1.011 | 0.908 |
| sp|P07742|RIR1_MOUSE | Ribonucleoside-diphosphate reductase large subunit OS=Mus musculus GN=Rrm1 PE=1 SV=2 | 70 | 0.892 | 0.795 | 0.622 |
| sp|P11679|K2C8_MOUSE | Keratin, type II cytoskeletal 8 OS=Mus musculus GN=Krt8 PE=1 SV=4 | 70 | 1.117 | 0.974 | 0.843 |
| sp|Q9CQV8|1433B_MOUSE | 14-3-3 protein beta/alpha OS=Mus musculus GN=Ywhab PE=1 SV=3 | 70 | 1.054 | 0.912 | 0.879 |
| sp|P35700|PRDX1_MOUSE | Peroxiredoxin-1 OS=Mus musculus GN=Prdx1 PE=1 SV=1 | 70 | 1.057 | 1.007 | 0.911 |
| sp|P14148|RL7_MOUSE | 60S ribosomal protein L7 OS=Mus musculus GN=Rpl7 PE=2 SV=2 | 70 | 1.077 | 1.047 | 0.938 |
| sp|Q6DFW4|NOP58_MOUSE | Nucleolar protein 58 OS=Mus musculus GN=Nop58 PE=1 SV=1 | 70 | 1.218 | 0.947 | 1.070 |
| sp|P62960|YBOX1_MOUSE | Nuclease-sensitive element-binding protein 1 OS=Mus musculus GN=Ybx1 PE=1 SV=3 | 69 | 1.111 | 0.911 | 0.524 |
| sp|Q9DCX2|ATP5H_MOUSE | ATP synthase subunit d, mitochondrial OS=Mus musculus GN=Atp5h PE=1 SV=3 | 69 | 1.023 | 0.874 | 0.891 |
| sp|Q9DBG3|AP2B1_MOUSE | AP-2 complex subunit beta OS=Mus musculus GN=Ap2b1 PE=1 SV=1 | 69 | 0.975 | 0.891 | 0.918 |
| sp|P47962|RL5_MOUSE | 60S ribosomal protein L5 OS=Mus musculus GN=Rpl5 PE=1 SV=3 | 69 | 1.090 | 1.034 | 0.921 |
| sp|P14131|RS16_MOUSE | 40S ribosomal protein S16 OS=Mus musculus GN=Rps16 PE=2 SV=4 | 69 | 1.056 | 1.014 | 0.972 |
| sp|P48997|INVO_MOUSE | Involucrin OS=Mus musculus GN=Ivl PE=1 SV=1 | 68 | 0.585 | 0.453 | 0.288 |
| sp|P61027|RAB10_MOUSE | Ras-related protein Rab-10 OS=Mus musculus GN=Rab10 PE=1 SV=1 | 68 | 1.019 | 0.905 | 0.728 |
| sp|P24472|GSTA4_MOUSE | Glutathione S-transferase A4 OS=Mus musculus GN=Gsta4 PE=1 SV=3 | 68 | 1.046 | 0.934 | 0.839 |
| sp|Q64433|CH10_MOUSE | 10 kDa heat shock protein, mitochondrial OS=Mus musculus GN=Hspe1 PE=1 SV=2 | 68 | 1.164 | 0.961 | 0.901 |
| sp|P60122|RUVB1_MOUSE | RuvB-like 1 OS=Mus musculus GN=Ruvbl1 PE=1 SV=1 | 68 | 1.024 | 1.038 | 0.912 |
| sp|P26516|PSD7_MOUSE | 26S proteasome non-ATPase regulatory subunit 7 OS=Mus musculus GN=Psmd7 PE=1 SV=2 | 68 | 1.192 | 0.973 | 0.937 |
| sp|Q9WTM5|RUVB2_MOUSE | RuvB-like 2 OS=Mus musculus GN=Ruvbl2 PE=2 SV=3 | 68 | 1.065 | 0.884 | 0.969 |
| sp|O88342|WDR1_MOUSE | WD repeat-containing protein 1 OS=Mus musculus GN=Wdr1 PE=1 SV=3 | 67 | 1.079 | 0.969 | 0.754 |
| sp|P53994|RAB2A_MOUSE | Ras-related protein Rab-2A OS=Mus musculus GN=Rab2a PE=1 SV=1 | 67 | 1.155 | 0.897 | 0.793 |
| sp|P0C0S6|H2AZ_MOUSE | Histone H2A.Z OS=Mus musculus GN=H2afz PE=1 SV=2 | 67 | 1.384 | 0.892 | 0.885 |
| sp|Q3THW5|H2AV_MOUSE | Histone H2A.V OS=Mus musculus GN=H2afv PE=1 SV=3 | 67 | 1.321 | 0.907 | 0.891 |
| sp|P61982|1433G_MOUSE | 14-3-3 protein gamma OS=Mus musculus GN=Ywhag PE=1 SV=2 | 67 | 1.090 | 0.860 | 0.926 |
| sp|Q9JLV5|CUL3_MOUSE | Cullin-3 OS=Mus musculus GN=Cul3 PE=1 SV=1 | 67 | 0.932 | 1.418 | 0.935 |
| sp|Q569Z6|TR150_MOUSE | Thyroid hormone receptor-associated protein 3 OS=Mus musculus GN=Thrap3 PE=1 SV=1 | 67 | 1.075 | 1.000 | 0.944 |
| sp|P49722|PSA2_MOUSE | Proteasome subunit alpha type-2 OS=Mus musculus GN=Psma2 PE=1 SV=3 | 67 | 1.163 | 1.076 | 1.052 |
| sp|P26231|CTNA1_MOUSE | Catenin alpha-1 OS=Mus musculus GN=Ctnna1 PE=1 SV=1 | 66 | 0.613 | 0.706 | 0.729 |
| sp|Q7TQI3|OTUB1_MOUSE | Ubiquitin thioesterase OTUB1 OS=Mus musculus GN=Otub1 PE=1 SV=2 | 66 | 0.985 | 0.971 | 0.878 |
| sp|P17047|LAMP2_MOUSE | Lysosome-associated membrane glycoprotein 2 OS=Mus musculus GN=Lamp2 PE=2 SV=2 | 66 | 0.916 | 1.042 | 0.888 |
| sp|Q922D8|C1TC_MOUSE | C-1-tetrahydrofolate synthase, cytoplasmic OS=Mus musculus GN=Mthfd1 PE=1 SV=4 | 66 | 0.992 | 0.900 | 0.910 |
| sp|P20108|PRDX3_MOUSE | Thioredoxin-dependent peroxide reductase, mitochondrial OS=Mus musculus GN=Prdx3 PE=1 SV=1 | 66 | 1.026 | 0.944 | 0.946 |
| sp|Q922R8|PDIA6_MOUSE | Protein disulfide-isomerase A6 OS=Mus musculus GN=Pdia6 PE=1 SV=3 | 66 | 1.237 | 1.108 | 0.946 |
| sp|P62631|EF1A2_MOUSE | Elongation factor 1-alpha 2 OS=Mus musculus GN=Eef1a2 PE=1 SV=1 | 66 | 0.000 | InD | InD |
| sp|Q91V92|ACLY_MOUSE | ATP-citrate synthase OS=Mus musculus GN=Acly PE=1 SV=1 | 65 | 1.141 | 1.038 | 0.685 |
| sp|P35979|RL12_MOUSE | 60S ribosomal protein L12 OS=Mus musculus GN=Rpl12 PE=1 SV=2 | 65 | 1.084 | 0.989 | 0.912 |
| sp|Q99PT1|GDIR1_MOUSE | Rho GDP-dissociation inhibitor 1 OS=Mus musculus GN=Arhgdia PE=1 SV=3 | 65 | 1.081 | 1.000 | 0.914 |
| sp|P18242|CATD_MOUSE | Cathepsin D OS=Mus musculus GN=Ctsd PE=1 SV=1 | 65 | 0.947 | 0.725 | 0.922 |
| sp|Q9DB77|QCR2_MOUSE | Cytochrome b-c1 complex subunit 2, mitochondrial OS=Mus musculus GN=Uqcrc2 PE=1 SV=1 | 65 | 1.221 | 0.918 | 0.951 |
| sp|Q9R0X4|ACOT9_MOUSE | Acyl-coenzyme A thioesterase 9, mitochondrial OS=Mus musculus GN=Acot9 PE=1 SV=1 | 65 | 1.144 | 1.000 | 0.953 |
| sp|Q9DBR7|MYPT1_MOUSE | Protein phosphatase 1 regulatory subunit 12A OS=Mus musculus GN=Ppp1r12a PE=1 SV=2 | 65 | 1.185 | 0.961 | 1.113 |
| sp|Q60605|MYL6_MOUSE | Myosin light polypeptide 6 OS=Mus musculus GN=Myl6 PE=1 SV=3 | 64 | 1.070 | 0.984 | 0.844 |
| sp|Q9JJI8|RL38_MOUSE | 60S ribosomal protein L38 OS=Mus musculus GN=Rpl38 PE=2 SV=3 | 64 | 1.102 | 1.074 | 0.846 |
| sp|Q9Z1Z0|USO1_MOUSE | General vesicular transport factor p115 OS=Mus musculus GN=Uso1 PE=1 SV=2 | 64 | 1.112 | 1.043 | 0.938 |
| sp|O55131|SEPT7_MOUSE | Septin-7 OS=Mus musculus GN=Sept7 PE=1 SV=1 | 64 | 1.081 | 1.029 | 0.940 |
| sp|P62751|RL23A_MOUSE | 60S ribosomal protein L23a OS=Mus musculus GN=Rpl23a PE=1 SV=1 | 63 | 1.109 | 0.955 | 0.671 |
| sp|Q9D6U8|F162A_MOUSE | Protein FAM162A OS=Mus musculus GN=Fam162a PE=2 SV=1 | 63 | 0.986 | 0.804 | 0.768 |
| sp|Q9CQQ7|AT5F1_MOUSE | ATP synthase subunit b, mitochondrial OS=Mus musculus GN=Atp5f1 PE=1 SV=1 | 63 | 1.195 | 0.916 | 0.787 |
| sp|P35278|RAB5C_MOUSE | Ras-related protein Rab-5C OS=Mus musculus GN=Rab5c PE=1 SV=2 | 63 | 1.079 | 0.939 | 0.809 |
| sp|Q99LG2|TNPO2_MOUSE | Transportin-2 OS=Mus musculus GN=Tnpo2 PE=2 SV=1 | 63 | 0.920 | 0.951 | 0.853 |
| sp|Q9CQ60|6PGL_MOUSE | 6-phosphogluconolactonase OS=Mus musculus GN=Pgls PE=2 SV=1 | 63 | 1.085 | 1.086 | 0.976 |
| sp|Q91ZJ5|UGPA_MOUSE | UTP--glucose-1-phosphate uridylyltransferase OS=Mus musculus GN=Ugp2 PE=2 SV=3 | 63 | 1.079 | 0.935 | 1.015 |
| sp|P09528|FRIH_MOUSE | Ferritin heavy chain OS=Mus musculus GN=Fth1 PE=1 SV=2 | 63 | 1.268 | 1.399 | 1.350 |
| sp|Q922J3|CLIP1_MOUSE | CAP-Gly domain-containing linker protein 1 OS=Mus musculus GN=Clip1 PE=1 SV=1 | 62 | 0.990 | 0.981 | 0.729 |
| sp|Q9JHR7|IDE_MOUSE | Insulin-degrading enzyme OS=Mus musculus GN=Ide PE=1 SV=1 | 62 | 1.087 | 1.270 | 0.822 |
| sp|Q9Z1N5|DX39B_MOUSE | Spliceosome RNA helicase Ddx39b OS=Mus musculus GN=Ddx39b PE=1 SV=1 | 62 | 1.000 | 1.008 | 0.942 |
| sp|Q9Z2U0|PSA7_MOUSE | Proteasome subunit alpha type-7 OS=Mus musculus GN=Psma7 PE=1 SV=1 | 62 | 1.261 | 1.014 | 0.971 |
| sp|Q9ERU9|RBP2_MOUSE | E3 SUMO-protein ligase RanBP2 OS=Mus musculus GN=Ranbp2 PE=1 SV=2 | 62 | 1.063 | InD | 0.981 |
| sp|Q02257|PLAK_MOUSE | Junction plakoglobin OS=Mus musculus GN=Jup PE=1 SV=3 | 61 | 0.842 | 0.509 | 0.520 |
| sp|P70670|NACAM_MOUSE | Nascent polypeptide-associated complex subunit alpha, muscle-specific form OS=Mus musculus GN=Naca PE=1 SV=2 | 61 | 1.187 | 0.958 | 0.946 |
| sp|Q60817|NACA_MOUSE | Nascent polypeptide-associated complex subunit alpha OS=Mus musculus GN=Naca PE=1 SV=1 | 61 | 1.202 | 0.949 | 0.964 |
| sp|Q61753|SERA_MOUSE | D-3-phosphoglycerate dehydrogenase OS=Mus musculus GN=Phgdh PE=1 SV=3 | 61 | 1.033 | 1.063 | 1.016 |
| sp|Q99PV0|PRP8_MOUSE | Pre-mRNA-processing-splicing factor 8 OS=Mus musculus GN=Prpf8 PE=1 SV=2 | 61 | 1.183 | 0.824 | 1.058 |
| sp|Q91WJ8|FUBP1_MOUSE | Far upstream element-binding protein 1 OS=Mus musculus GN=Fubp1 PE=1 SV=1 | 61 | 1.143 | 1.082 | 1.065 |
| sp|Q6PDM2|SRSF1_MOUSE | Serine/arginine-rich splicing factor 1 OS=Mus musculus GN=Srsf1 PE=1 SV=3 | 60 | 0.908 | 0.581 | 0.871 |
| sp|P60867|RS20_MOUSE | 40S ribosomal protein S20 OS=Mus musculus GN=Rps20 PE=1 SV=1 | 60 | 1.065 | 0.977 | 0.881 |
| sp|P57776|EF1D_MOUSE | Elongation factor 1-delta OS=Mus musculus GN=Eef1d PE=1 SV=3 | 60 | 1.088 | 1.098 | 0.890 |
| sp|Q9EQU5|SET_MOUSE | Protein SET OS=Mus musculus GN=Set PE=1 SV=1 | 60 | 0.952 | 0.745 | 1.057 |
| sp|Q91VI7|RINI_MOUSE | Ribonuclease inhibitor OS=Mus musculus GN=Rnh1 PE=1 SV=1 | 59 | 0.948 | 0.897 | 0.736 |
| sp|Q9CZ30|OLA1_MOUSE | Obg-like ATPase 1 OS=Mus musculus GN=Ola1 PE=1 SV=1 | 59 | 1.000 | 1.062 | 0.792 |
| sp|Q99LC5|ETFA_MOUSE | Electron transfer flavoprotein subunit alpha, mitochondrial OS=Mus musculus GN=Etfa PE=1 SV=2 | 59 | 1.020 | 0.858 | 0.820 |
| sp|Q62470|ITA3_MOUSE | Integrin alpha-3 OS=Mus musculus GN=Itga3 PE=1 SV=1 | 59 | 0.990 | 1.011 | 0.858 |
| sp|P30999|CTND1_MOUSE | Catenin delta-1 OS=Mus musculus GN=Ctnnd1 PE=1 SV=2 | 59 | 0.970 | 0.872 | 0.868 |
| sp|Q8BFY9|TNPO1_MOUSE | Transportin-1 OS=Mus musculus GN=Tnpo1 PE=1 SV=2 | 59 | 1.058 | 0.890 | 0.922 |
| sp|Q9D8W5|PSD12_MOUSE | 26S proteasome non-ATPase regulatory subunit 12 OS=Mus musculus GN=Psmd12 PE=1 SV=4 | 59 | 1.095 | 0.968 | 0.927 |
| sp|Q8QZY1|EIF3L_MOUSE | Eukaryotic translation initiation factor 3 subunit L OS=Mus musculus GN=Eif3l PE=1 SV=1 | 59 | 1.171 | 0.909 | 0.958 |
| sp|Q62318|TIF1B_MOUSE | Transcription intermediary factor 1-beta OS=Mus musculus GN=Trim28 PE=1 SV=3 | 59 | 0.935 | 0.933 | 1.033 |
| sp|Q91VR2|ATPG_MOUSE | ATP synthase subunit gamma, mitochondrial OS=Mus musculus GN=Atp5c1 PE=1 SV=1 | 58 | 1.053 | 0.851 | 0.763 |
| sp|P24369|PPIB_MOUSE | Peptidyl-prolyl cis-trans isomerase B OS=Mus musculus GN=Ppib PE=2 SV=2 | 58 | 1.092 | 0.839 | 0.796 |
| sp|P97855|G3BP1_MOUSE | Ras GTPase-activating protein-binding protein 1 OS=Mus musculus GN=G3bp1 PE=1 SV=1 | 58 | 1.218 | 0.771 | 0.831 |
| sp|O35887|CALU_MOUSE | Calumenin OS=Mus musculus GN=Calu PE=1 SV=1 | 58 | 1.146 | 0.938 | 0.836 |
| sp|Q6PDI5|ECM29_MOUSE | Proteasome-associated protein ECM29 homolog OS=Mus musculus GN=Ecm29 PE=1 SV=3 | 58 | 1.099 | 0.916 | 0.862 |
| sp|P61255|RL26_MOUSE | 60S ribosomal protein L26 OS=Mus musculus GN=Rpl26 PE=2 SV=1 | 58 | 1.116 | 1.000 | 0.872 |
| sp|P12382|K6PL_MOUSE | 6-phosphofructokinase, liver type OS=Mus musculus GN=Pfkl PE=1 SV=4 | 58 | 1.288 | 0.962 | 0.906 |
| sp|P10854|H2B1M_MOUSE | Histone H2B type 1-M OS=Mus musculus GN=Hist1h2bm PE=1 SV=2 | 58 | 0.931 | 0.936 | 0.908 |
| sp|P10853|H2B1F_MOUSE | Histone H2B type 1-F/J/L OS=Mus musculus GN=Hist1h2bf PE=1 SV=2 | 58 | 0.927 | 0.943 | 0.914 |
| sp|Q64475|H2B1B_MOUSE | Histone H2B type 1-B OS=Mus musculus GN=Hist1h2bb PE=1 SV=3 | 58 | 0.927 | 0.943 | 0.914 |
| sp|Q64478|H2B1H_MOUSE | Histone H2B type 1-H OS=Mus musculus GN=Hist1h2bh PE=1 SV=3 | 58 | 0.927 | 0.943 | 0.914 |
| sp|Q64525|H2B2B_MOUSE | Histone H2B type 2-B OS=Mus musculus GN=Hist2h2bb PE=1 SV=3 | 58 | 0.927 | 0.943 | 0.914 |
| sp|Q6ZWY9|H2B1C_MOUSE | Histone H2B type 1-C/E/G OS=Mus musculus GN=Hist1h2bc PE=1 SV=3 | 58 | 0.927 | 0.943 | 0.914 |
| sp|Q8CGP1|H2B1K_MOUSE | Histone H2B type 1-K OS=Mus musculus GN=Hist1h2bk PE=1 SV=3 | 58 | 0.927 | 0.943 | 0.914 |
| sp|Q8CGP2|H2B1P_MOUSE | Histone H2B type 1-P OS=Mus musculus GN=Hist1h2bp PE=1 SV=3 | 58 | 0.927 | 0.943 | 0.914 |
| sp|P17710|HXK1_MOUSE | Hexokinase-1 OS=Mus musculus GN=Hk1 PE=1 SV=3 | 58 | 1.088 | 1.035 | 0.947 |
| sp|P68037|UB2L3_MOUSE | Ubiquitin-conjugating enzyme E2 L3 OS=Mus musculus GN=Ube2l3 PE=2 SV=1 | 58 | 1.043 | 1.013 | 0.956 |
| sp|Q8BHN3|GANAB_MOUSE | Neutral alpha-glucosidase AB OS=Mus musculus GN=Ganab PE=1 SV=1 | 58 | 1.202 | 1.047 | 1.014 |
| sp|Q5SW19|CLU_MOUSE | Clustered mitochondria protein homolog OS=Mus musculus GN=Kiaa0664 PE=2 SV=2 | 57 | 0.881 | 0.871 | 0.845 |
| sp|Q3TXS7|PSMD1_MOUSE | 26S proteasome non-ATPase regulatory subunit 1 OS=Mus musculus GN=Psmd1 PE=1 SV=1 | 57 | 1.092 | 1.094 | 0.888 |
| sp|P05201|AATC_MOUSE | Aspartate aminotransferase, cytoplasmic OS=Mus musculus GN=Got1 PE=1 SV=3 | 57 | 1.032 | 0.949 | 0.893 |
| sp|Q9D819|IPYR_MOUSE | Inorganic pyrophosphatase OS=Mus musculus GN=Ppa1 PE=1 SV=1 | 57 | 1.063 | 1.218 | 0.905 |
| sp|P84104|SRSF3_MOUSE | Serine/arginine-rich splicing factor 3 OS=Mus musculus GN=Srsf3 PE=1 SV=1 | 57 | 0.992 | 0.873 | 0.922 |
| sp|P60766|CDC42_MOUSE | Cell division control protein 42 homolog OS=Mus musculus GN=Cdc42 PE=1 SV=2 | 57 | 1.127 | 0.922 | 0.952 |
| sp|Q8BVE3|VATH_MOUSE | V-type proton ATPase subunit H OS=Mus musculus GN=Atp6v1h PE=1 SV=1 | 57 | 1.138 | 1.060 | 1.000 |
| sp|Q9CQN1|TRAP1_MOUSE | Heat shock protein 75 kDa, mitochondrial OS=Mus musculus GN=Trap1 PE=1 SV=1 | 56 | 0.979 | 0.848 | 0.876 |
| sp|P61021|RAB5B_MOUSE | Ras-related protein Rab-5B OS=Mus musculus GN=Rab5b PE=1 SV=1 | 56 | 1.051 | 0.973 | 0.894 |
| sp|P62852|RS25_MOUSE | 40S ribosomal protein S25 OS=Mus musculus GN=Rps25 PE=2 SV=1 | 56 | 1.129 | 1.000 | 0.947 |
| sp|P24452|CAPG_MOUSE | Macrophage-capping protein OS=Mus musculus GN=Capg PE=1 SV=2 | 56 | 1.065 | 1.018 | 0.991 |
| sp|Q8R081|HNRPL_MOUSE | Heterogeneous nuclear ribonucleoprotein L OS=Mus musculus GN=Hnrnpl PE=1 SV=2 | 56 | 0.900 | 0.856 | 0.993 |
| sp|Q9Z0F7|SYUG_MOUSE | Gamma-synuclein OS=Mus musculus GN=Sncg PE=1 SV=1 | 56 | 1.663 | 1.730 | 2.403 |
| sp|P46664|PURA2_MOUSE | Adenylosuccinate synthetase isozyme 2 OS=Mus musculus GN=Adss PE=1 SV=2 | 55 | 0.909 | 0.770 | 0.482 |
| sp|Q9JKB3|DBPA_MOUSE | DNA-binding protein A OS=Mus musculus GN=Csda PE=1 SV=2 | 55 | 0.936 | 0.871 | 0.523 |
| sp|Q9DBP5|KCY_MOUSE | UMP-CMP kinase OS=Mus musculus GN=Cmpk1 PE=1 SV=1 | 55 | 0.985 | 1.032 | 0.785 |
| sp|Q9CPR4|RL17_MOUSE | 60S ribosomal protein L17 OS=Mus musculus GN=Rpl17 PE=2 SV=3 | 55 | 1.200 | 0.828 | 0.804 |
| sp|O89023|TPP1_MOUSE | Tripeptidyl-peptidase 1 OS=Mus musculus GN=Tpp1 PE=1 SV=2 | 55 | 0.954 | 0.898 | 0.837 |
| sp|Q9ERK4|XPO2_MOUSE | Exportin-2 OS=Mus musculus GN=Cse1l PE=2 SV=1 | 55 | 0.950 | 0.810 | 0.867 |
| sp|P62192|PRS4_MOUSE | 26S protease regulatory subunit 4 OS=Mus musculus GN=Psmc1 PE=1 SV=1 | 55 | 1.235 | 1.023 | 0.873 |
| sp|P63158|HMGB1_MOUSE | High mobility group protein B1 OS=Mus musculus GN=Hmgb1 PE=1 SV=2 | 55 | 1.053 | 0.978 | 0.875 |
| sp|Q9CW03|SMC3_MOUSE | Structural maintenance of chromosomes protein 3 OS=Mus musculus GN=Smc3 PE=1 SV=2 | 55 | 0.944 | 1.084 | 0.918 |
| sp|P62897|CYC_MOUSE | Cytochrome c, somatic OS=Mus musculus GN=Cycs PE=1 SV=2 | 55 | 1.119 | 1.010 | 0.936 |
| sp|Q921F2|TADBP_MOUSE | TAR DNA-binding protein 43 OS=Mus musculus GN=Tardbp PE=1 SV=1 | 55 | 1.150 | 0.993 | 1.038 |
| sp|P70698|PYRG1_MOUSE | CTP synthase 1 OS=Mus musculus GN=Ctps1 PE=1 SV=2 | 55 | 1.283 | 1.375 | 1.309 |
| sp|P24549|AL1A1_MOUSE | Retinal dehydrogenase 1 OS=Mus musculus GN=Aldh1a1 PE=1 SV=5 | 54 | 0.598 | 0.510 | 0.248 |
| sp|Q8BWT1|THIM_MOUSE | 3-ketoacyl-CoA thiolase, mitochondrial OS=Mus musculus GN=Acaa2 PE=1 SV=3 | 54 | 0.924 | 0.738 | 0.620 |
| sp|Q62393|TPD52_MOUSE | Tumor protein D52 OS=Mus musculus GN=Tpd52 PE=1 SV=2 | 54 | 1.012 | 0.881 | 0.762 |
| sp|Q99020|ROAA_MOUSE | Heterogeneous nuclear ribonucleoprotein A/B OS=Mus musculus GN=Hnrnpab PE=1 SV=1 | 54 | 0.911 | 0.941 | 0.820 |
| sp|P54071|IDHP_MOUSE | Isocitrate dehydrogenase [NADP], mitochondrial OS=Mus musculus GN=Idh2 PE=1 SV=3 | 54 | 1.039 | 1.027 | 0.856 |
| sp|P39447|ZO1_MOUSE | Tight junction protein ZO-1 OS=Mus musculus GN=Tjp1 PE=1 SV=2 | 54 | 1.136 | 1.036 | 0.895 |
| sp|P47911|RL6_MOUSE | 60S ribosomal protein L6 OS=Mus musculus GN=Rpl6 PE=1 SV=3 | 53 | 1.100 | 0.861 | 0.719 |
| sp|P32067|LA_MOUSE | Lupus La protein homolog OS=Mus musculus GN=Ssb PE=2 SV=1 | 53 | 1.172 | 0.918 | 0.786 |
| sp|Q8BP47|SYNC_MOUSE | Asparagine--tRNA ligase, cytoplasmic OS=Mus musculus GN=Nars PE=1 SV=2 | 53 | 1.538 | 1.130 | 0.886 |
| sp|P14685|PSMD3_MOUSE | 26S proteasome non-ATPase regulatory subunit 3 OS=Mus musculus GN=Psmd3 PE=1 SV=3 | 53 | 0.938 | 1.122 | 0.977 |
| sp|P28656|NP1L1_MOUSE | Nucleosome assembly protein 1-like 1 OS=Mus musculus GN=Nap1l1 PE=1 SV=2 | 53 | 1.214 | 1.039 | 0.978 |
| sp|Q921M3|SF3B3_MOUSE | Splicing factor 3B subunit 3 OS=Mus musculus GN=Sf3b3 PE=2 SV=1 | 53 | 1.239 | 1.190 | 1.000 |
| sp|Q99MN1|SYK_MOUSE | Lysine--tRNA ligase OS=Mus musculus GN=Kars PE=1 SV=1 | 53 | 1.295 | 1.092 | 1.021 |
| sp|Q9Z110|P5CS_MOUSE | Delta-1-pyrroline-5-carboxylate synthase OS=Mus musculus GN=Aldh18a1 PE=2 SV=2 | 53 | 1.078 | 1.111 | 1.096 |
| sp|O08810|U5S1_MOUSE | 116 kDa U5 small nuclear ribonucleoprotein component OS=Mus musculus GN=Eftud2 PE=2 SV=1 | 53 | 0.915 | 0.952 | 1.185 |
| sp|P08207|S10AA_MOUSE | Protein S100-A10 OS=Mus musculus GN=S100a10 PE=2 SV=2 | 53 | 1.165 | 0.997 | 1.324 |
| sp|P48722|HS74L_MOUSE | Heat shock 70 kDa protein 4L OS=Mus musculus GN=Hspa4l PE=1 SV=2 | 53 | 1.537 | 1.290 | 1.591 |
| sp|P10649|GSTM1_MOUSE | Glutathione S-transferase Mu 1 OS=Mus musculus GN=Gstm1 PE=1 SV=2 | 52 | 0.672 | 0.588 | 0.285 |
| sp|Q99JY0|ECHB_MOUSE | Trifunctional enzyme subunit beta, mitochondrial OS=Mus musculus GN=Hadhb PE=1 SV=1 | 52 | 0.990 | 0.858 | 0.689 |
| sp|Q62418|DBNL_MOUSE | Drebrin-like protein OS=Mus musculus GN=Dbnl PE=1 SV=2 | 52 | 0.960 | 0.867 | 0.794 |
| sp|Q68FL6|SYMC_MOUSE | Methionine--tRNA ligase, cytoplasmic OS=Mus musculus GN=Mars PE=2 SV=1 | 52 | 1.057 | 1.010 | 0.820 |
| sp|Q9Z1Q5|CLIC1_MOUSE | Chloride intracellular channel protein 1 OS=Mus musculus GN=Clic1 PE=1 SV=3 | 52 | 1.048 | 0.922 | 0.824 |
| sp|Q64331|MYO6_MOUSE | Unconventional myosin-VI OS=Mus musculus GN=Myo6 PE=1 SV=1 | 52 | 1.096 | 0.933 | 0.841 |
| sp|P14206|RSSA_MOUSE | 40S ribosomal protein SA OS=Mus musculus GN=Rpsa PE=1 SV=4 | 52 | 0.989 | 1.088 | 0.944 |
| sp|Q921M7|FA49B_MOUSE | Protein FAM49B OS=Mus musculus GN=Fam49b PE=2 SV=1 | 52 | 1.174 | 1.065 | 1.000 |
| sp|P62196|PRS8_MOUSE | 26S protease regulatory subunit 8 OS=Mus musculus GN=Psmc5 PE=1 SV=1 | 52 | 1.138 | 1.074 | 1.000 |
| sp|Q9CQF3|CPSF5_MOUSE | Cleavage and polyadenylation specificity factor subunit 5 OS=Mus musculus GN=Nudt21 PE=2 SV=1 | 52 | 1.134 | 0.913 | 1.022 |
| sp|Q9R0P5|DEST_MOUSE | Destrin OS=Mus musculus GN=Dstn PE=1 SV=3 | 52 | 1.275 | 1.345 | 1.259 |
| sp|Q9EPL8|IPO7_MOUSE | Importin-7 OS=Mus musculus GN=Ipo7 PE=1 SV=2 | 51 | 1.105 | 1.119 | 0.828 |
| sp|O55029|COPB2_MOUSE | Coatomer subunit beta&#039; OS=Mus musculus GN=Copb2 PE=2 SV=2 | 51 | 0.976 | 0.968 | 0.893 |
| sp|P50396|GDIA_MOUSE | Rab GDP dissociation inhibitor alpha OS=Mus musculus GN=Gdi1 PE=1 SV=3 | 51 | 1.105 | InD | 0.905 |
| sp|Q9ER72|SYCC_MOUSE | Cysteine--tRNA ligase, cytoplasmic OS=Mus musculus GN=Cars PE=1 SV=2 | 51 | 1.171 | 0.915 | 1.011 |
| sp|Q99K85|SERC_MOUSE | Phosphoserine aminotransferase OS=Mus musculus GN=Psat1 PE=1 SV=1 | 51 | 1.211 | 1.048 | 1.132 |
| sp|Q93092|TALDO_MOUSE | Transaldolase OS=Mus musculus GN=Taldo1 PE=1 SV=2 | 50 | 1.088 | 0.986 | 0.759 |
| sp|P62918|RL8_MOUSE | 60S ribosomal protein L8 OS=Mus musculus GN=Rpl8 PE=2 SV=2 | 50 | 1.200 | 0.922 | 0.829 |
| sp|P62242|RS8_MOUSE | 40S ribosomal protein S8 OS=Mus musculus GN=Rps8 PE=1 SV=2 | 50 | 1.046 | 0.976 | 0.892 |
| sp|O55022|PGRC1_MOUSE | Membrane-associated progesterone receptor component 1 OS=Mus musculus GN=Pgrmc1 PE=1 SV=4 | 50 | 1.056 | 0.893 | 0.910 |
| sp|P61222|ABCE1_MOUSE | ATP-binding cassette sub-family E member 1 OS=Mus musculus GN=Abce1 PE=2 SV=1 | 50 | 1.132 | 0.989 | 0.915 |
| sp|Q9JJ28|FLII_MOUSE | Protein flightless-1 homolog OS=Mus musculus GN=Flii PE=1 SV=1 | 50 | 1.108 | 0.849 | 0.934 |
| sp|P62264|RS14_MOUSE | 40S ribosomal protein S14 OS=Mus musculus GN=Rps14 PE=2 SV=3 | 50 | 1.165 | 0.988 | 0.967 |
| sp|P29758|OAT_MOUSE | Ornithine aminotransferase, mitochondrial OS=Mus musculus GN=Oat PE=1 SV=1 | 50 | 1.161 | 1.067 | 0.978 |
| sp|Q9WUA2|SYFB_MOUSE | Phenylalanine--tRNA ligase beta subunit OS=Mus musculus GN=Farsb PE=2 SV=2 | 50 | 1.193 | 1.117 | 1.010 |
| sp|Q8BGD9|IF4B_MOUSE | Eukaryotic translation initiation factor 4B OS=Mus musculus GN=Eif4b PE=1 SV=1 | 49 | 1.051 | 1.108 | 0.714 |
| sp|Q91V41|RAB14_MOUSE | Ras-related protein Rab-14 OS=Mus musculus GN=Rab14 PE=1 SV=3 | 49 | 0.981 | 0.906 | 0.771 |
| sp|Q9CQX2|CYB5B_MOUSE | Cytochrome b5 type B OS=Mus musculus GN=Cyb5b PE=1 SV=1 | 49 | 1.075 | 1.029 | 0.821 |
| sp|P58771|TPM1_MOUSE | Tropomyosin alpha-1 chain OS=Mus musculus GN=Tpm1 PE=1 SV=1 | 49 | 0.992 | 0.919 | 0.829 |
| sp|Q9JM14|NT5C_MOUSE | 5&#039;(3&#039;)-deoxyribonucleotidase, cytosolic type OS=Mus musculus GN=Nt5c PE=1 SV=1 | 49 | 1.017 | 0.930 | 0.876 |
| sp|P47757|CAPZB_MOUSE | F-actin-capping protein subunit beta OS=Mus musculus GN=Capzb PE=1 SV=3 | 49 | 1.000 | 0.874 | 0.881 |
| sp|Q62348|TSN_MOUSE | Translin OS=Mus musculus GN=Tsn PE=1 SV=1 | 49 | 1.113 | 1.093 | 1.016 |
| sp|Q00612|G6PD1_MOUSE | Glucose-6-phosphate 1-dehydrogenase X OS=Mus musculus GN=G6pdx PE=1 SV=3 | 48 | 0.791 | 0.806 | 0.483 |
| sp|Q9D154|ILEUA_MOUSE | Leukocyte elastase inhibitor A OS=Mus musculus GN=Serpinb1a PE=1 SV=1 | 48 | 0.903 | 0.981 | 0.725 |
| sp|Q99KJ8|DCTN2_MOUSE | Dynactin subunit 2 OS=Mus musculus GN=Dctn2 PE=1 SV=3 | 48 | 1.045 | 0.959 | 0.747 |
| sp|Q9D6R2|IDH3A_MOUSE | Isocitrate dehydrogenase [NAD] subunit alpha, mitochondrial OS=Mus musculus GN=Idh3a PE=1 SV=1 | 48 | 1.057 | 0.909 | 0.783 |
| sp|Q9Z2I0|LETM1_MOUSE | LETM1 and EF-hand domain-containing protein 1, mitochondrial OS=Mus musculus GN=Letm1 PE=2 SV=1 | 48 | 1.140 | 1.131 | 0.790 |
| sp|Q9R0P3|ESTD_MOUSE | S-formylglutathione hydrolase OS=Mus musculus GN=Esd PE=2 SV=1 | 48 | 0.945 | 0.901 | 0.821 |
| sp|Q9CPY7|AMPL_MOUSE | Cytosol aminopeptidase OS=Mus musculus GN=Lap3 PE=1 SV=3 | 48 | 0.967 | 0.889 | 0.826 |
| sp|Q91YH5|ATLA3_MOUSE | Atlastin-3 OS=Mus musculus GN=Atl3 PE=2 SV=1 | 48 | 1.201 | 0.966 | 0.854 |
| sp|Q8BL97|SRSF7_MOUSE | Serine/arginine-rich splicing factor 7 OS=Mus musculus GN=Srsf7 PE=1 SV=1 | 48 | 1.331 | 0.875 | 0.868 |
| sp|P62245|RS15A_MOUSE | 40S ribosomal protein S15a OS=Mus musculus GN=Rps15a PE=2 SV=2 | 48 | 1.167 | 0.931 | 0.882 |
| sp|Q3THE2|ML12B_MOUSE | Myosin regulatory light chain 12B OS=Mus musculus GN=Myl12b PE=1 SV=2 | 48 | 1.087 | 1.031 | 0.895 |
| sp|Q9JM76|ARPC3_MOUSE | Actin-related protein 2/3 complex subunit 3 OS=Mus musculus GN=Arpc3 PE=1 SV=3 | 48 | 0.969 | 1.050 | 0.922 |
| sp|Q7M6Y3|PICA_MOUSE | Phosphatidylinositol-binding clathrin assembly protein OS=Mus musculus GN=Picalm PE=1 SV=1 | 48 | 1.189 | 0.918 | 0.953 |
| sp|Q1HFZ0|NSUN2_MOUSE | tRNA (cytosine(34)-C(5))-methyltransferase OS=Mus musculus GN=Nsun2 PE=1 SV=2 | 48 | 1.143 | 1.172 | 0.973 |
| sp|P54823|DDX6_MOUSE | Probable ATP-dependent RNA helicase DDX6 OS=Mus musculus GN=Ddx6 PE=2 SV=1 | 48 | 1.048 | 0.919 | 1.081 |
| sp|P56395|CYB5_MOUSE | Cytochrome b5 OS=Mus musculus GN=Cyb5a PE=1 SV=2 | 47 | 1.083 | 0.888 | 0.750 |
| sp|Q9CQM9|GLRX3_MOUSE | Glutaredoxin-3 OS=Mus musculus GN=Glrx3 PE=1 SV=1 | 47 | 1.149 | 1.020 | 0.824 |
| sp|Q91VD9|NDUS1_MOUSE | NADH-ubiquinone oxidoreductase 75 kDa subunit, mitochondrial OS=Mus musculus GN=Ndufs1 PE=1 SV=2 | 47 | 1.041 | 0.869 | 0.867 |
| sp|P51410|RL9_MOUSE | 60S ribosomal protein L9 OS=Mus musculus GN=Rpl9 PE=2 SV=2 | 47 | 1.139 | 0.946 | 0.906 |
| sp|Q9Z2W0|DNPEP_MOUSE | Aspartyl aminopeptidase OS=Mus musculus GN=Dnpep PE=2 SV=2 | 47 | 1.173 | 0.948 | 0.933 |
| sp|Q8VDW0|DX39A_MOUSE | ATP-dependent RNA helicase DDX39A OS=Mus musculus GN=Ddx39a PE=2 SV=1 | 47 | InD | 1.157 | 0.955 |
| sp|O08528|HXK2_MOUSE | Hexokinase-2 OS=Mus musculus GN=Hk2 PE=1 SV=1 | 47 | 1.176 | 1.162 | 1.154 |
| sp|Q99PL5|RRBP1_MOUSE | Ribosome-binding protein 1 OS=Mus musculus GN=Rrbp1 PE=2 SV=2 | 46 | 1.240 | 1.051 | 0.531 |
| sp|Q99KN9|EPN4_MOUSE | Clathrin interactor 1 OS=Mus musculus GN=Clint1 PE=1 SV=2 | 46 | 1.606 | 0.972 | 0.674 |
| sp|Q78ZA7|NP1L4_MOUSE | Nucleosome assembly protein 1-like 4 OS=Mus musculus GN=Nap1l4 PE=1 SV=1 | 46 | 0.868 | 1.161 | 0.721 |
| sp|P62900|RL31_MOUSE | 60S ribosomal protein L31 OS=Mus musculus GN=Rpl31 PE=2 SV=1 | 46 | 0.972 | 0.917 | 0.756 |
| sp|P39054|DYN2_MOUSE | Dynamin-2 OS=Mus musculus GN=Dnm2 PE=1 SV=2 | 46 | 1.028 | 0.855 | 0.844 |
| sp|Q8BMA6|SRP68_MOUSE | Signal recognition particle 68 kDa protein OS=Mus musculus GN=Srp68 PE=2 SV=2 | 46 | 1.053 | 1.202 | 0.951 |
| sp|P17427|AP2A2_MOUSE | AP-2 complex subunit alpha-2 OS=Mus musculus GN=Ap2a2 PE=1 SV=2 | 46 | 0.936 | 0.830 | 0.968 |
| sp|P61358|RL27_MOUSE | 60S ribosomal protein L27 OS=Mus musculus GN=Rpl27 PE=2 SV=2 | 46 | 1.087 | 1.055 | 0.985 |
| sp|Q9DBG5|PLIN3_MOUSE | Perilipin-3 OS=Mus musculus GN=Plin3 PE=1 SV=1 | 45 | 0.921 | 0.810 | 0.777 |
| sp|Q9CZM2|RL15_MOUSE | 60S ribosomal protein L15 OS=Mus musculus GN=Rpl15 PE=2 SV=4 | 45 | 1.114 | 0.958 | 0.817 |
| sp|Q7TMB8|CYFP1_MOUSE | Cytoplasmic FMR1-interacting protein 1 OS=Mus musculus GN=Cyfip1 PE=1 SV=1 | 45 | 1.058 | 0.980 | 0.852 |
| sp|P63028|TCTP_MOUSE | Translationally-controlled tumor protein OS=Mus musculus GN=Tpt1 PE=1 SV=1 | 45 | 1.142 | 1.094 | 0.882 |
| sp|O70251|EF1B_MOUSE | Elongation factor 1-beta OS=Mus musculus GN=Eef1b PE=1 SV=5 | 45 | 1.069 | 1.033 | 0.910 |
| sp|Q8CGF7|TCRG1_MOUSE | Transcription elongation regulator 1 OS=Mus musculus GN=Tcerg1 PE=1 SV=2 | 45 | 1.171 | 0.949 | 1.000 |
| sp|P62761|VISL1_MOUSE | Visinin-like protein 1 OS=Mus musculus GN=Vsnl1 PE=1 SV=2 | 45 | 1.120 | 1.073 | 1.055 |
| sp|P97807|FUMH_MOUSE | Fumarate hydratase, mitochondrial OS=Mus musculus GN=Fh PE=1 SV=3 | 44 | 1.000 | 0.958 | 0.767 |
| sp|Q9CZ44|NSF1C_MOUSE | NSFL1 cofactor p47 OS=Mus musculus GN=Nsfl1c PE=1 SV=1 | 44 | 1.134 | 0.783 | 0.989 |
| sp|O35381|AN32A_MOUSE | Acidic leucine-rich nuclear phosphoprotein 32 family member A OS=Mus musculus GN=Anp32a PE=1 SV=1 | 44 | 1.440 | 1.008 | 0.997 |
| sp|Q91WK2|EIF3H_MOUSE | Eukaryotic translation initiation factor 3 subunit H OS=Mus musculus GN=Eif3h PE=1 SV=1 | 44 | 1.156 | 1.100 | 1.010 |
| sp|Q9EQK5|MVP_MOUSE | Major vault protein OS=Mus musculus GN=Mvp PE=1 SV=4 | 44 | 1.453 | 0.860 | 1.017 |
| sp|Q8BYA0|TBCD_MOUSE | Tubulin-specific chaperone D OS=Mus musculus GN=Tbcd PE=2 SV=1 | 44 | 1.139 | 1.270 | 1.018 |
| sp|Q922B2|SYDC_MOUSE | Aspartate--tRNA ligase, cytoplasmic OS=Mus musculus GN=Dars PE=2 SV=2 | 44 | 1.108 | 0.924 | 1.092 |
| sp|P61750|ARF4_MOUSE | ADP-ribosylation factor 4 OS=Mus musculus GN=Arf4 PE=2 SV=2 | 44 | 1.160 | 1.047 | InD |
| sp|P62774|MTPN_MOUSE | Myotrophin OS=Mus musculus GN=Mtpn PE=1 SV=2 | 43 | 1.101 | 0.976 | 0.824 |
| sp|Q9R1P4|PSA1_MOUSE | Proteasome subunit alpha type-1 OS=Mus musculus GN=Psma1 PE=1 SV=1 | 43 | 1.421 | 0.874 | 0.831 |
| sp|Q9DCN2|NB5R3_MOUSE | NADH-cytochrome b5 reductase 3 OS=Mus musculus GN=Cyb5r3 PE=1 SV=3 | 43 | 0.935 | 1.042 | 0.847 |
| sp|P09671|SODM_MOUSE | Superoxide dismutase [Mn], mitochondrial OS=Mus musculus GN=Sod2 PE=1 SV=3 | 43 | 1.120 | 0.975 | 0.887 |
| sp|Q9QXK3|COPG2_MOUSE | Coatomer subunit gamma-2 OS=Mus musculus GN=Copg2 PE=2 SV=1 | 43 | 0.993 | InD | 0.898 |
| sp|Q99LP6|GRPE1_MOUSE | GrpE protein homolog 1, mitochondrial OS=Mus musculus GN=Grpel1 PE=1 SV=1 | 43 | 1.000 | 0.897 | 0.902 |
| sp|Q9Z1F9|SAE2_MOUSE | SUMO-activating enzyme subunit 2 OS=Mus musculus GN=Uba2 PE=2 SV=1 | 43 | 1.071 | 0.753 | 0.934 |
| sp|O09167|RL21_MOUSE | 60S ribosomal protein L21 OS=Mus musculus GN=Rpl21 PE=2 SV=3 | 43 | 1.197 | 0.976 | 0.949 |
| sp|Q9DCH4|EIF3F_MOUSE | Eukaryotic translation initiation factor 3 subunit F OS=Mus musculus GN=Eif3f PE=1 SV=2 | 43 | 1.128 | 1.022 | 1.074 |
| sp|O08529|CAN2_MOUSE | Calpain-2 catalytic subunit OS=Mus musculus GN=Capn2 PE=2 SV=4 | 42 | 1.013 | 0.815 | 0.888 |
| sp|P10605|CATB_MOUSE | Cathepsin B OS=Mus musculus GN=Ctsb PE=1 SV=2 | 42 | 0.935 | 0.886 | 0.912 |
| sp|Q80X50|UBP2L_MOUSE | Ubiquitin-associated protein 2-like OS=Mus musculus GN=Ubap2l PE=1 SV=1 | 42 | 1.154 | 0.796 | 0.956 |
| sp|P23927|CRYAB_MOUSE | Alpha-crystallin B chain OS=Mus musculus GN=Cryab PE=1 SV=2 | 42 | 1.259 | 0.878 | 0.975 |
| sp|O88544|CSN4_MOUSE | COP9 signalosome complex subunit 4 OS=Mus musculus GN=Cops4 PE=1 SV=1 | 42 | 1.087 | 0.924 | 0.986 |
| sp|Q9CQD1|RAB5A_MOUSE | Ras-related protein Rab-5A OS=Mus musculus GN=Rab5a PE=1 SV=1 | 41 | 1.061 | 0.987 | 0.805 |
| sp|Q9CWK8|SNX2_MOUSE | Sorting nexin-2 OS=Mus musculus GN=Snx2 PE=1 SV=2 | 41 | 1.161 | 0.968 | 0.851 |
| sp|P35550|FBRL_MOUSE | rRNA 2&#039;-O-methyltransferase fibrillarin OS=Mus musculus GN=Fbl PE=1 SV=2 | 41 | 1.034 | 1.073 | 0.965 |
| sp|Q99KQ4|NAMPT_MOUSE | Nicotinamide phosphoribosyltransferase OS=Mus musculus GN=Nampt PE=1 SV=1 | 41 | 1.155 | 0.961 | 1.186 |
| sp|P10852|4F2_MOUSE | 4F2 cell-surface antigen heavy chain OS=Mus musculus GN=Slc3a2 PE=1 SV=1 | 41 | 1.233 | 1.362 | 1.481 |
| sp|Q148V8|FA83H_MOUSE | Protein FAM83H OS=Mus musculus GN=Fam83h PE=1 SV=1 | 40 | 0.989 | 0.738 | 0.629 |
| sp|Q8CIT9|SBSN_MOUSE | Suprabasin OS=Mus musculus GN=Sbsn PE=2 SV=1 | 40 | 0.702 | 0.769 | 0.653 |
| sp|Q62433|NDRG1_MOUSE | Protein NDRG1 OS=Mus musculus GN=Ndrg1 PE=1 SV=1 | 40 | 1.079 | 0.827 | 0.740 |
| sp|P67984|RL22_MOUSE | 60S ribosomal protein L22 OS=Mus musculus GN=Rpl22 PE=2 SV=2 | 40 | 1.125 | 1.000 | 0.923 |
| sp|P26638|SYSC_MOUSE | Serine--tRNA ligase, cytoplasmic OS=Mus musculus GN=Sars PE=2 SV=3 | 40 | 0.878 | 1.408 | 1.034 |
| sp|O08756|HCD2_MOUSE | 3-hydroxyacyl-CoA dehydrogenase type-2 OS=Mus musculus GN=Hsd17b10 PE=1 SV=4 | 40 | 1.240 | 0.859 | 1.184 |
| sp|Q02819|NUCB1_MOUSE | Nucleobindin-1 OS=Mus musculus GN=Nucb1 PE=1 SV=2 | 39 | 0.852 | 0.888 | 0.573 |
| sp|Q8VCT3|AMPB_MOUSE | Aminopeptidase B OS=Mus musculus GN=Rnpep PE=2 SV=2 | 39 | 1.030 | 0.687 | 0.710 |
| sp|P12787|COX5A_MOUSE | Cytochrome c oxidase subunit 5A, mitochondrial OS=Mus musculus GN=Cox5a PE=1 SV=2 | 39 | 1.137 | 1.023 | 0.737 |
| sp|Q61207|SAP_MOUSE | Sulfated glycoprotein 1 OS=Mus musculus GN=Psap PE=1 SV=2 | 39 | 1.246 | 0.948 | 0.784 |
| sp|O54734|OST48_MOUSE | Dolichyl-diphosphooligosaccharide--protein glycosyltransferase 48 kDa subunit OS=Mus musculus GN=Ddost PE=1 SV=2 | 39 | 1.244 | 1.163 | 0.856 |
| sp|P49817|CAV1_MOUSE | Caveolin-1 OS=Mus musculus GN=Cav1 PE=1 SV=1 | 39 | 1.157 | 0.837 | 0.867 |
| sp|P59999|ARPC4_MOUSE | Actin-related protein 2/3 complex subunit 4 OS=Mus musculus GN=Arpc4 PE=1 SV=3 | 39 | 1.089 | 0.962 | 0.886 |
| sp|Q9CU62|SMC1A_MOUSE | Structural maintenance of chromosomes protein 1A OS=Mus musculus GN=Smc1a PE=1 SV=4 | 39 | 0.981 | 0.898 | 0.891 |
| sp|E9Q634|MYO1E_MOUSE | Unconventional myosin-Ie OS=Mus musculus GN=Myo1e PE=1 SV=1 | 39 | 1.139 | 0.925 | 0.904 |
| sp|Q9QYJ0|DNJA2_MOUSE | DnaJ homolog subfamily A member 2 OS=Mus musculus GN=Dnaja2 PE=1 SV=1 | 39 | 1.052 | 1.074 | 0.913 |
| sp|Q91VR5|DDX1_MOUSE | ATP-dependent RNA helicase DDX1 OS=Mus musculus GN=Ddx1 PE=1 SV=1 | 39 | 1.062 | 0.765 | 0.916 |
| sp|Q99JI6|RAP1B_MOUSE | Ras-related protein Rap-1b OS=Mus musculus GN=Rap1b PE=2 SV=2 | 39 | 1.033 | 1.065 | 0.918 |
| sp|P62835|RAP1A_MOUSE | Ras-related protein Rap-1A OS=Mus musculus GN=Rap1a PE=2 SV=1 | 39 | 1.047 | 1.065 | 0.918 |
| sp|Q3THS6|METK2_MOUSE | S-adenosylmethionine synthase isoform type-2 OS=Mus musculus GN=Mat2a PE=2 SV=2 | 39 | 1.055 | 1.011 | 0.926 |
| sp|Q00519|XDH_MOUSE | Xanthine dehydrogenase/oxidase OS=Mus musculus GN=Xdh PE=1 SV=5 | 39 | 1.589 | 1.489 | 1.472 |
| sp|P50543|S10AB_MOUSE | Protein S100-A11 OS=Mus musculus GN=S100a11 PE=2 SV=1 | 39 | 1.331 | 1.065 | 1.550 |
| sp|Q9JI91|ACTN2_MOUSE | Alpha-actinin-2 OS=Mus musculus GN=Actn2 PE=1 SV=2 | 39 | 0.938 | InD | InD |
| sp|Q6A026|PDS5A_MOUSE | Sister chromatid cohesion protein PDS5 homolog A OS=Mus musculus GN=Pds5a PE=2 SV=3 | 38 | 1.123 | 1.061 | 0.755 |
| sp|O88844|IDHC_MOUSE | Isocitrate dehydrogenase [NADP] cytoplasmic OS=Mus musculus GN=Idh1 PE=1 SV=2 | 38 | 1.009 | 0.932 | 0.784 |
| sp|Q9CQE8|CN166_MOUSE | UPF0568 protein C14orf166 homolog OS=Mus musculus PE=2 SV=1 | 38 | 1.147 | 1.024 | 0.809 |
| sp|Q60854|SPB6_MOUSE | Serpin B6 OS=Mus musculus GN=Serpinb6 PE=2 SV=1 | 38 | 1.000 | 0.858 | 0.852 |
| sp|P11438|LAMP1_MOUSE | Lysosome-associated membrane glycoprotein 1 OS=Mus musculus GN=Lamp1 PE=1 SV=2 | 38 | 0.892 | 1.146 | 0.900 |
| sp|Q9CR62|M2OM_MOUSE | Mitochondrial 2-oxoglutarate/malate carrier protein OS=Mus musculus GN=Slc25a11 PE=1 SV=3 | 38 | 1.309 | 0.807 | 0.900 |
| sp|Q9WVJ2|PSD13_MOUSE | 26S proteasome non-ATPase regulatory subunit 13 OS=Mus musculus GN=Psmd13 PE=1 SV=1 | 38 | 1.039 | 1.010 | 0.904 |
| sp|Q99JI4|PSMD6_MOUSE | 26S proteasome non-ATPase regulatory subunit 6 OS=Mus musculus GN=Psmd6 PE=1 SV=1 | 38 | 1.072 | 0.979 | 0.919 |
| sp|O08547|SC22B_MOUSE | Vesicle-trafficking protein SEC22b OS=Mus musculus GN=Sec22b PE=1 SV=3 | 38 | 1.369 | 1.033 | 0.937 |
| sp|P52760|UK114_MOUSE | Ribonuclease UK114 OS=Mus musculus GN=Hrsp12 PE=1 SV=3 | 38 | 0.914 | 0.983 | 0.939 |
| sp|O08808|DIAP1_MOUSE | Protein diaphanous homolog 1 OS=Mus musculus GN=Diaph1 PE=1 SV=1 | 38 | 1.034 | 0.904 | 0.959 |
| sp|P57746|VATD_MOUSE | V-type proton ATPase subunit D OS=Mus musculus GN=Atp6v1d PE=1 SV=1 | 38 | 1.109 | 1.121 | 0.967 |
| sp|P51859|HDGF_MOUSE | Hepatoma-derived growth factor OS=Mus musculus GN=Hdgf PE=1 SV=2 | 38 | 1.241 | 0.912 | 0.995 |
| sp|P50518|VATE1_MOUSE | V-type proton ATPase subunit E 1 OS=Mus musculus GN=Atp6v1e1 PE=1 SV=2 | 38 | 1.193 | 1.110 | 1.032 |
| sp|Q60931|VDAC3_MOUSE | Voltage-dependent anion-selective channel protein 3 OS=Mus musculus GN=Vdac3 PE=1 SV=1 | 38 | 1.039 | 1.059 | 1.045 |
| sp|P17426|AP2A1_MOUSE | AP-2 complex subunit alpha-1 OS=Mus musculus GN=Ap2a1 PE=1 SV=1 | 38 | 1.022 | 0.794 | 1.063 |
| sp|O88685|PRS6A_MOUSE | 26S protease regulatory subunit 6A OS=Mus musculus GN=Psmc3 PE=1 SV=2 | 38 | 1.015 | 1.289 | 1.885 |
| sp|Q91XV3|BASP1_MOUSE | Brain acid soluble protein 1 OS=Mus musculus GN=Basp1 PE=1 SV=3 | 38 | 1.533 | 1.400 | 1.917 |
| sp|P49312|ROA1_MOUSE | Heterogeneous nuclear ribonucleoprotein A1 OS=Mus musculus GN=Hnrnpa1 PE=1 SV=2 | 37 | 1.057 | 0.832 | 0.512 |
| sp|Q60598|SRC8_MOUSE | Src substrate cortactin OS=Mus musculus GN=Cttn PE=1 SV=2 | 37 | 1.027 | 0.934 | 0.717 |
| sp|Q9D1G1|RAB1B_MOUSE | Ras-related protein Rab-1B OS=Mus musculus GN=Rab1b PE=1 SV=1 | 37 | 1.368 | 0.880 | 0.780 |
| sp|Q62425|NDUA4_MOUSE | NADH dehydrogenase [ubiquinone] 1 alpha subcomplex subunit 4 OS=Mus musculus GN=Ndufa4 PE=1 SV=2 | 37 | 1.147 | 0.911 | 0.861 |
| sp|Q99L13|3HIDH_MOUSE | 3-hydroxyisobutyrate dehydrogenase, mitochondrial OS=Mus musculus GN=Hibadh PE=2 SV=1 | 37 | 1.028 | 0.982 | 0.938 |
| sp|Q8R2Y8|PTH2_MOUSE | Peptidyl-tRNA hydrolase 2, mitochondrial OS=Mus musculus GN=Ptrh2 PE=2 SV=1 | 37 | 1.092 | 0.891 | 0.963 |
| sp|Q8C1B7|SEP11_MOUSE | Septin-11 OS=Mus musculus GN=Sept11 PE=1 SV=4 | 37 | 1.015 | 1.107 | 0.964 |
| sp|Q6PGB6|NAA50_MOUSE | N-alpha-acetyltransferase 50 OS=Mus musculus GN=Naa50 PE=1 SV=1 | 37 | 1.021 | 1.216 | 0.970 |
| sp|Q9D1A2|CNDP2_MOUSE | Cytosolic non-specific dipeptidase OS=Mus musculus GN=Cndp2 PE=1 SV=1 | 37 | 0.931 | 0.903 | 0.972 |
| sp|Q8VED5|K2C79_MOUSE | Keratin, type II cytoskeletal 79 OS=Mus musculus GN=Krt79 PE=1 SV=2 | 36 | 2.194 | 1.038 | 0.674 |
| sp|Q8CI51|PDLI5_MOUSE | PDZ and LIM domain protein 5 OS=Mus musculus GN=Pdlim5 PE=1 SV=4 | 36 | 1.049 | 0.918 | 0.686 |
| sp|Q9JHJ0|TMOD3_MOUSE | Tropomodulin-3 OS=Mus musculus GN=Tmod3 PE=1 SV=1 | 36 | 0.996 | 0.818 | 0.717 |
| sp|P30275|KCRU_MOUSE | Creatine kinase U-type, mitochondrial OS=Mus musculus GN=Ckmt1 PE=1 SV=1 | 36 | 1.014 | 0.821 | 0.781 |
| sp|Q60668|HNRPD_MOUSE | Heterogeneous nuclear ribonucleoprotein D0 OS=Mus musculus GN=Hnrnpd PE=1 SV=2 | 36 | 1.082 | 0.813 | 0.801 |
| sp|Q921M4|GOGA2_MOUSE | Golgin subfamily A member 2 OS=Mus musculus GN=Golga2 PE=1 SV=3 | 36 | 1.064 | 0.868 | 0.848 |
| sp|Q60930|VDAC2_MOUSE | Voltage-dependent anion-selective channel protein 2 OS=Mus musculus GN=Vdac2 PE=1 SV=2 | 36 | 0.898 | 0.817 | 0.880 |
| sp|Q8BMF4|ODP2_MOUSE | Dihydrolipoyllysine-residue acetyltransferase component of pyruvate dehydrogenase complex, mitochondrial OS=Mus musculus GN=Dlat PE=1 SV=2 | 36 | 1.132 | 0.952 | 0.938 |
| sp|P68510|1433F_MOUSE | 14-3-3 protein eta OS=Mus musculus GN=Ywhah PE=1 SV=2 | 36 | 0.926 | 0.899 | 0.947 |
| sp|P61924|COPZ1_MOUSE | Coatomer subunit zeta-1 OS=Mus musculus GN=Copz1 PE=2 SV=1 | 36 | 1.072 | 1.110 | 0.980 |
| sp|Q61024|ASNS_MOUSE | Asparagine synthetase [glutamine-hydrolyzing] OS=Mus musculus GN=Asns PE=2 SV=3 | 36 | 1.056 | 0.775 | 1.083 |
| sp|Q9JMA1|UBP14_MOUSE | Ubiquitin carboxyl-terminal hydrolase 14 OS=Mus musculus GN=Usp14 PE=1 SV=3 | 36 | 1.000 | 1.068 | 1.103 |
| sp|Q80UG5|SEPT9_MOUSE | Septin-9 OS=Mus musculus GN=Sept9 PE=1 SV=1 | 36 | 1.365 | 1.137 | 1.205 |
| sp|O70404|VAMP8_MOUSE | Vesicle-associated membrane protein 8 OS=Mus musculus GN=Vamp8 PE=1 SV=1 | 35 | 0.849 | 0.811 | 0.735 |
| sp|P58871|TB182_MOUSE | 182 kDa tankyrase-1-binding protein OS=Mus musculus GN=Tnks1bp1 PE=1 SV=2 | 35 | 0.975 | 0.733 | 0.748 |
| sp|Q64152|BTF3_MOUSE | Transcription factor BTF3 OS=Mus musculus GN=Btf3 PE=2 SV=3 | 35 | 1.094 | 1.017 | 0.782 |
| sp|Q9CXW4|RL11_MOUSE | 60S ribosomal protein L11 OS=Mus musculus GN=Rpl11 PE=1 SV=4 | 35 | 1.084 | 0.969 | 0.935 |
| sp|Q810B6|ANFY1_MOUSE | Ankyrin repeat and FYVE domain-containing protein 1 OS=Mus musculus GN=Ankfy1 PE=2 SV=2 | 35 | 1.314 | 0.984 | 0.994 |
| sp|P63276|RS17_MOUSE | 40S ribosomal protein S17 OS=Mus musculus GN=Rps17 PE=1 SV=2 | 35 | 0.989 | 1.012 | 1.011 |
| sp|P61087|UBE2K_MOUSE | Ubiquitin-conjugating enzyme E2 K OS=Mus musculus GN=Ube2k PE=1 SV=3 | 35 | 1.101 | 0.885 | 1.014 |
| sp|Q64737|PUR2_MOUSE | Trifunctional purine biosynthetic protein adenosine-3 OS=Mus musculus GN=Gart PE=2 SV=3 | 35 | 1.240 | 1.294 | 1.151 |
| sp|P97350|PKP1_MOUSE | Plakophilin-1 OS=Mus musculus GN=Pkp1 PE=1 SV=1 | 34 | 0.913 | 0.552 | 0.525 |
| sp|Q9DC37|MFSD1_MOUSE | Major facilitator superfamily domain-containing protein 1 OS=Mus musculus GN=Mfsd1 PE=1 SV=1 | 34 | InD | 0.853 | 0.788 |
| sp|P08074|CBR2_MOUSE | Carbonyl reductase [NADPH] 2 OS=Mus musculus GN=Cbr2 PE=1 SV=1 | 34 | 1.092 | 1.000 | 0.794 |
| sp|Q9R1P3|PSB2_MOUSE | Proteasome subunit beta type-2 OS=Mus musculus GN=Psmb2 PE=1 SV=1 | 34 | 1.144 | 1.041 | 0.795 |
| sp|P28352|APEX1_MOUSE | DNA-(apurinic or apyrimidinic site) lyase OS=Mus musculus GN=Apex1 PE=1 SV=2 | 34 | 1.041 | 0.737 | 0.795 |
| sp|Q9R1P1|PSB3_MOUSE | Proteasome subunit beta type-3 OS=Mus musculus GN=Psmb3 PE=1 SV=1 | 34 | 1.268 | 1.121 | 0.827 |
| sp|P26645|MARCS_MOUSE | Myristoylated alanine-rich C-kinase substrate OS=Mus musculus GN=Marcks PE=1 SV=2 | 34 | 0.881 | 0.803 | 0.829 |
| sp|Q9CR60|GOT1B_MOUSE | Vesicle transport protein GOT1B OS=Mus musculus GN=Golt1b PE=2 SV=1 | 34 | 1.160 | 1.167 | 0.846 |
| sp|Q8VEH3|ARL8A_MOUSE | ADP-ribosylation factor-like protein 8A OS=Mus musculus GN=Arl8a PE=2 SV=1 | 34 | 0.951 | 0.826 | 0.858 |
| sp|Q9CX86|ROA0_MOUSE | Heterogeneous nuclear ribonucleoprotein A0 OS=Mus musculus GN=Hnrnpa0 PE=1 SV=1 | 34 | 1.500 | 0.629 | 0.862 |
| sp|P47955|RLA1_MOUSE | 60S acidic ribosomal protein P1 OS=Mus musculus GN=Rplp1 PE=1 SV=1 | 34 | 1.081 | 1.048 | 0.907 |
| sp|Q07563|COHA1_MOUSE | Collagen alpha-1(XVII) chain OS=Mus musculus GN=Col17a1 PE=1 SV=3 | 34 | 1.286 | 0.963 | 0.949 |
| sp|P11352|GPX1_MOUSE | Glutathione peroxidase 1 OS=Mus musculus GN=Gpx1 PE=1 SV=2 | 34 | 1.063 | 1.058 | 1.076 |
| sp|P45591|COF2_MOUSE | Cofilin-2 OS=Mus musculus GN=Cfl2 PE=1 SV=1 | 34 | 1.157 | 1.101 | 1.179 |
| sp|P08030|APT_MOUSE | Adenine phosphoribosyltransferase OS=Mus musculus GN=Aprt PE=2 SV=1 | 34 | 1.271 | 1.429 | 1.241 |
| sp|O70400|PDLI1_MOUSE | PDZ and LIM domain protein 1 OS=Mus musculus GN=Pdlim1 PE=2 SV=4 | 34 | 1.329 | 0.628 | 1.247 |
| sp|P29391|FRIL1_MOUSE | Ferritin light chain 1 OS=Mus musculus GN=Ftl1 PE=1 SV=2 | 34 | 1.148 | 2.274 | 1.893 |
| sp|O54782|MA2B2_MOUSE | Epididymis-specific alpha-mannosidase OS=Mus musculus GN=Man2b2 PE=2 SV=2 | 33 | 0.947 | 0.701 | 0.614 |
| sp|Q99J99|THTM_MOUSE | 3-mercaptopyruvate sulfurtransferase OS=Mus musculus GN=Mpst PE=1 SV=3 | 33 | 0.899 | 0.861 | 0.713 |
| sp|Q9D172|ES1_MOUSE | ES1 protein homolog, mitochondrial OS=Mus musculus GN=D10Jhu81e PE=1 SV=1 | 33 | 0.955 | 0.745 | 0.722 |
| sp|Q3TKT4|SMCA4_MOUSE | Transcription activator BRG1 OS=Mus musculus GN=Smarca4 PE=1 SV=1 | 33 | 1.085 | 0.000 | 0.730 |
| sp|Q9QXX4|CMC2_MOUSE | Calcium-binding mitochondrial carrier protein Aralar2 OS=Mus musculus GN=Slc25a13 PE=1 SV=1 | 33 | InD | 0.886 | 0.840 |
| sp|P55258|RAB8A_MOUSE | Ras-related protein Rab-8A OS=Mus musculus GN=Rab8a PE=1 SV=2 | 33 | 1.040 | 0.941 | 0.850 |
| sp|O88487|DC1I2_MOUSE | Cytoplasmic dynein 1 intermediate chain 2 OS=Mus musculus GN=Dync1i2 PE=2 SV=1 | 33 | 0.976 | 0.975 | 0.903 |
| sp|P53026|RL10A_MOUSE | 60S ribosomal protein L10a OS=Mus musculus GN=Rpl10a PE=1 SV=3 | 33 | 1.056 | 1.022 | 0.937 |
| sp|Q3V3R1|C1TM_MOUSE | Monofunctional C1-tetrahydrofolate synthase, mitochondrial OS=Mus musculus GN=Mthfd1l PE=1 SV=2 | 33 | 1.136 | 0.883 | 0.986 |
| sp|P42208|SEPT2_MOUSE | Septin-2 OS=Mus musculus GN=Sept2 PE=1 SV=2 | 33 | 1.157 | 1.137 | 1.076 |
| sp|P23198|CBX3_MOUSE | Chromobox protein homolog 3 OS=Mus musculus GN=Cbx3 PE=1 SV=2 | 33 | 0.956 | 1.120 | 1.114 |
| sp|Q9CQW2|ARL8B_MOUSE | ADP-ribosylation factor-like protein 8B OS=Mus musculus GN=Arl8b PE=2 SV=1 | 33 | 0.938 | 0.978 | InD |
| sp|Q8VCT4|CES1D_MOUSE | Carboxylesterase 1D OS=Mus musculus GN=Ces1d PE=1 SV=1 | 32 | 0.527 | 0.670 | 0.348 |
| sp|O89001|CBPD_MOUSE | Carboxypeptidase D OS=Mus musculus GN=Cpd PE=1 SV=2 | 32 | 0.927 | 0.709 | 0.600 |
| sp|P21619|LMNB2_MOUSE | Lamin-B2 OS=Mus musculus GN=Lmnb2 PE=1 SV=2 | 32 | 1.059 | 0.674 | 0.672 |
| sp|Q8JZQ2|AFG32_MOUSE | AFG3-like protein 2 OS=Mus musculus GN=Afg3l2 PE=1 SV=1 | 32 | 0.782 | 1.124 | 0.700 |
| sp|Q8K019|BCLF1_MOUSE | Bcl-2-associated transcription factor 1 OS=Mus musculus GN=Bclaf1 PE=1 SV=2 | 32 | 1.000 | 1.111 | 0.705 |
| sp|Q61166|MARE1_MOUSE | Microtubule-associated protein RP/EB family member 1 OS=Mus musculus GN=Mapre1 PE=1 SV=3 | 32 | 1.184 | 1.438 | 0.750 |
| sp|Q9WUA3|K6PP_MOUSE | 6-phosphofructokinase type C OS=Mus musculus GN=Pfkp PE=1 SV=1 | 32 | 1.189 | 1.000 | 0.835 |
| sp|Q9D7S7|RL22L_MOUSE | 60S ribosomal protein L22-like 1 OS=Mus musculus GN=Rpl22l1 PE=1 SV=1 | 32 | 1.109 | 1.022 | 0.836 |
| sp|Q923D2|BLVRB_MOUSE | Flavin reductase (NADPH) OS=Mus musculus GN=Blvrb PE=2 SV=3 | 32 | 1.098 | 1.053 | 0.851 |
| sp|Q8C129|LCAP_MOUSE | Leucyl-cystinyl aminopeptidase OS=Mus musculus GN=Lnpep PE=1 SV=1 | 32 | 1.000 | 0.915 | 0.865 |
| sp|P54728|RD23B_MOUSE | UV excision repair protein RAD23 homolog B OS=Mus musculus GN=Rad23b PE=1 SV=2 | 32 | 0.978 | 1.188 | 0.910 |
| sp|Q7TMY8|HUWE1_MOUSE | E3 ubiquitin-protein ligase HUWE1 OS=Mus musculus GN=Huwe1 PE=1 SV=5 | 32 | 1.250 | 1.323 | 0.930 |
| sp|P14115|RL27A_MOUSE | 60S ribosomal protein L27a OS=Mus musculus GN=Rpl27a PE=2 SV=5 | 32 | 1.173 | 0.973 | 0.932 |
| sp|O35295|PURB_MOUSE | Transcriptional activator protein Pur-beta OS=Mus musculus GN=Purb PE=1 SV=3 | 32 | 0.966 | 3.022 | 0.937 |
| sp|O55135|IF6_MOUSE | Eukaryotic translation initiation factor 6 OS=Mus musculus GN=Eif6 PE=1 SV=2 | 32 | 1.219 | 1.103 | 0.949 |
| sp|Q9CZ13|QCR1_MOUSE | Cytochrome b-c1 complex subunit 1, mitochondrial OS=Mus musculus GN=Uqcrc1 PE=1 SV=2 | 32 | 0.961 | 1.053 | 0.950 |
| sp|Q91VT4|CBR4_MOUSE | Carbonyl reductase family member 4 OS=Mus musculus GN=Cbr4 PE=2 SV=2 | 32 | 1.051 | 1.050 | 0.955 |
| sp|P60335|PCBP1_MOUSE | Poly(rC)-binding protein 1 OS=Mus musculus GN=Pcbp1 PE=1 SV=1 | 32 | 0.952 | 1.000 | 0.978 |
| sp|Q99L45|IF2B_MOUSE | Eukaryotic translation initiation factor 2 subunit 2 OS=Mus musculus GN=Eif2s2 PE=1 SV=1 | 32 | 1.086 | 0.963 | 0.989 |
| sp|P60670|NPL4_MOUSE | Nuclear protein localization protein 4 homolog OS=Mus musculus GN=Nploc4 PE=1 SV=3 | 32 | 1.229 | 1.034 | 1.034 |
| sp|Q5SYD0|MYO1D_MOUSE | Unconventional myosin-Id OS=Mus musculus GN=Myo1d PE=1 SV=1 | 32 | 1.209 | 1.209 | 1.097 |
| sp|P27612|PLAP_MOUSE | Phospholipase A-2-activating protein OS=Mus musculus GN=Plaa PE=2 SV=4 | 32 | 1.239 | 1.375 | 1.213 |
| sp|Q9D3D9|ATPD_MOUSE | ATP synthase subunit delta, mitochondrial OS=Mus musculus GN=Atp5d PE=1 SV=1 | 31 | 1.333 | 0.783 | 0.649 |
| sp|Q58A65|JIP4_MOUSE | C-Jun-amino-terminal kinase-interacting protein 4 OS=Mus musculus GN=Spag9 PE=1 SV=2 | 31 | 1.071 | 1.023 | 0.780 |
| sp|P62855|RS26_MOUSE | 40S ribosomal protein S26 OS=Mus musculus GN=Rps26 PE=2 SV=3 | 31 | 1.000 | 0.800 | 0.806 |
| sp|P31786|ACBP_MOUSE | Acyl-CoA-binding protein OS=Mus musculus GN=Dbi PE=1 SV=2 | 31 | 1.077 | 1.048 | 0.902 |
| sp|Q9R1P0|PSA4_MOUSE | Proteasome subunit alpha type-4 OS=Mus musculus GN=Psma4 PE=1 SV=1 | 31 | 1.186 | 1.048 | 0.922 |
| sp|Q569Z5|DDX46_MOUSE | Probable ATP-dependent RNA helicase DDX46 OS=Mus musculus GN=Ddx46 PE=1 SV=2 | 31 | 0.912 | 0.907 | 1.025 |
| sp|Q9CQ80|VPS25_MOUSE | Vacuolar protein-sorting-associated protein 25 OS=Mus musculus GN=Vps25 PE=2 SV=1 | 31 | 0.969 | 1.052 | 1.031 |
| sp|Q9Z2I8|SUCB2_MOUSE | Succinyl-CoA ligase [GDP-forming] subunit beta, mitochondrial OS=Mus musculus GN=Suclg2 PE=2 SV=3 | 30 | 1.038 | 0.947 | 0.756 |
| sp|P36552|HEM6_MOUSE | Coproporphyrinogen-III oxidase, mitochondrial OS=Mus musculus GN=Cpox PE=1 SV=2 | 30 | 1.131 | 1.131 | 0.760 |
| sp|Q9WV55|VAPA_MOUSE | Vesicle-associated membrane protein-associated protein A OS=Mus musculus GN=Vapa PE=1 SV=2 | 30 | 0.682 | 0.789 | 0.762 |
| sp|Q8VDP6|CDIPT_MOUSE | CDP-diacylglycerol--inositol 3-phosphatidyltransferase OS=Mus musculus GN=Cdipt PE=1 SV=1 | 30 | 1.285 | 0.983 | 0.776 |
| sp|P43024|CX6A1_MOUSE | Cytochrome c oxidase subunit 6A1, mitochondrial OS=Mus musculus GN=Cox6a1 PE=1 SV=2 | 30 | 0.929 | 0.954 | 0.828 |
| sp|P47791|GSHR_MOUSE | Glutathione reductase, mitochondrial OS=Mus musculus GN=Gsr PE=2 SV=3 | 30 | 1.062 | 0.857 | 0.843 |
| sp|Q8BJY1|PSMD5_MOUSE | 26S proteasome non-ATPase regulatory subunit 5 OS=Mus musculus GN=Psmd5 PE=1 SV=4 | 30 | 1.050 | 0.787 | 0.854 |
| sp|Q3B7Z2|OSBP1_MOUSE | Oxysterol-binding protein 1 OS=Mus musculus GN=Osbp PE=1 SV=2 | 30 | 1.032 | 0.883 | 0.854 |
| sp|Q9Z2U1|PSA5_MOUSE | Proteasome subunit alpha type-5 OS=Mus musculus GN=Psma5 PE=1 SV=1 | 30 | 1.128 | 1.133 | 0.904 |
| sp|P62334|PRS10_MOUSE | 26S protease regulatory subunit 10B OS=Mus musculus GN=Psmc6 PE=1 SV=1 | 30 | 1.298 | 0.916 | 0.918 |
| sp|Q9D2Q8|S10AE_MOUSE | Protein S100-A14 OS=Mus musculus GN=S100a14 PE=2 SV=1 | 30 | 0.885 | 0.883 | 0.926 |
| sp|Q9CQM5|TXD17_MOUSE | Thioredoxin domain-containing protein 17 OS=Mus musculus GN=Txndc17 PE=1 SV=1 | 30 | 1.074 | 1.037 | 0.954 |
| sp|P12815|PDCD6_MOUSE | Programmed cell death protein 6 OS=Mus musculus GN=Pdcd6 PE=1 SV=2 | 30 | 1.115 | 0.984 | 0.969 |
| sp|O88456|CPNS1_MOUSE | Calpain small subunit 1 OS=Mus musculus GN=Capns1 PE=2 SV=1 | 30 | 1.044 | 1.009 | 0.971 |
| sp|Q8K1J6|TRNT1_MOUSE | CCA tRNA nucleotidyltransferase 1, mitochondrial OS=Mus musculus GN=Trnt1 PE=2 SV=1 | 30 | 1.113 | 1.035 | 0.975 |
| sp|Q9WUM3|COR1B_MOUSE | Coronin-1B OS=Mus musculus GN=Coro1b PE=1 SV=1 | 30 | 1.109 | 1.251 | 0.993 |
| sp|Q9D0J8|PTMS_MOUSE | Parathymosin OS=Mus musculus GN=Ptms PE=2 SV=3 | 30 | 1.003 | 1.014 | 1.017 |
| sp|P61957|SUMO2_MOUSE | Small ubiquitin-related modifier 2 OS=Mus musculus GN=Sumo2 PE=2 SV=1 | 30 | 0.979 | 0.986 | 1.063 |
| sp|Q6P5B0|RRP12_MOUSE | RRP12-like protein OS=Mus musculus GN=Rrp12 PE=1 SV=1 | 30 | 0.877 | 0.943 | 1.169 |
| sp|Q8R0W0|EPIPL_MOUSE | Epiplakin OS=Mus musculus GN=Eppk1 PE=1 SV=2 | 29 | 1.818 | 0.493 | 0.500 |
| sp|Q8R5C5|ACTY_MOUSE | Beta-centractin OS=Mus musculus GN=Actr1b PE=1 SV=1 | 29 | InD | 1.006 | 0.741 |
| sp|Q06185|ATP5I_MOUSE | ATP synthase subunit e, mitochondrial OS=Mus musculus GN=Atp5i PE=1 SV=2 | 29 | 1.048 | 0.868 | 0.766 |
| sp|P61164|ACTZ_MOUSE | Alpha-centractin OS=Mus musculus GN=Actr1a PE=2 SV=1 | 29 | 1.096 | InD | 0.778 |
| sp|P22892|AP1G1_MOUSE | AP-1 complex subunit gamma-1 OS=Mus musculus GN=Ap1g1 PE=1 SV=3 | 29 | 1.007 | 0.907 | 0.807 |
| sp|P61089|UBE2N_MOUSE | Ubiquitin-conjugating enzyme E2 N OS=Mus musculus GN=Ube2n PE=1 SV=1 | 29 | 0.858 | 0.946 | 0.850 |
| sp|Q9D662|SC23B_MOUSE | Protein transport protein Sec23B OS=Mus musculus GN=Sec23b PE=2 SV=1 | 29 | 0.902 | 1.365 | 0.880 |
| sp|Q9CZ04|CSN7A_MOUSE | COP9 signalosome complex subunit 7a OS=Mus musculus GN=Cops7a PE=1 SV=2 | 29 | 1.125 | 0.939 | 0.930 |
| sp|P97822|AN32E_MOUSE | Acidic leucine-rich nuclear phosphoprotein 32 family member E OS=Mus musculus GN=Anp32e PE=1 SV=2 | 29 | 1.045 | 0.900 | 0.933 |
| sp|Q99KP6|PRP19_MOUSE | Pre-mRNA-processing factor 19 OS=Mus musculus GN=Prpf19 PE=2 SV=1 | 29 | 1.438 | 0.935 | 0.961 |
| sp|Q62186|SSRD_MOUSE | Translocon-associated protein subunit delta OS=Mus musculus GN=Ssr4 PE=2 SV=1 | 29 | 1.060 | 0.978 | 0.968 |
| sp|Q5XJY5|COPD_MOUSE | Coatomer subunit delta OS=Mus musculus GN=Arcn1 PE=2 SV=2 | 29 | 1.036 | 1.278 | 1.238 |
| sp|Q9QZM0|UBQL2_MOUSE | Ubiquilin-2 OS=Mus musculus GN=Ubqln2 PE=1 SV=2 | 29 | 1.057 | 1.026 | 1.583 |
| sp|Q8VHX6|FLNC_MOUSE | Filamin-C OS=Mus musculus GN=Flnc PE=1 SV=3 | 29 | InD | InD | InD |
| sp|P09055|ITB1_MOUSE | Integrin beta-1 OS=Mus musculus GN=Itgb1 PE=1 SV=1 | 28 | 1.016 | 0.829 | 0.748 |
| sp|P62141|PP1B_MOUSE | Serine/threonine-protein phosphatase PP1-beta catalytic subunit OS=Mus musculus GN=Ppp1cb PE=1 SV=3 | 28 | 0.000 | InD | 0.800 |
| sp|Q60872|IF1A_MOUSE | Eukaryotic translation initiation factor 1A OS=Mus musculus GN=Eif1a PE=2 SV=3 | 28 | 0.895 | 0.805 | 0.823 |
| sp|Q8BMJ3|IF1AX_MOUSE | Eukaryotic translation initiation factor 1A, X-chromosomal OS=Mus musculus GN=Eif1ax PE=2 SV=3 | 28 | 0.895 | 0.831 | 0.829 |
| sp|Q3UM45|PP1R7_MOUSE | Protein phosphatase 1 regulatory subunit 7 OS=Mus musculus GN=Ppp1r7 PE=1 SV=2 | 28 | 1.074 | 1.000 | 0.830 |
| sp|Q8BY87|UBP47_MOUSE | Ubiquitin carboxyl-terminal hydrolase 47 OS=Mus musculus GN=Usp47 PE=1 SV=2 | 28 | 0.985 | 0.820 | 0.899 |
| sp|Q91YL7|PG2IP_MOUSE | PGAP2-interacting protein OS=Mus musculus GN=Cwh43 PE=1 SV=1 | 28 | 0.940 | 0.763 | 0.907 |
| sp|P62717|RL18A_MOUSE | 60S ribosomal protein L18a OS=Mus musculus GN=Rpl18a PE=1 SV=1 | 28 | 1.257 | 0.900 | 0.938 |
| sp|Q9CQ65|MTAP_MOUSE | S-methyl-5&#039;-thioadenosine phosphorylase OS=Mus musculus GN=Mtap PE=2 SV=1 | 28 | 1.175 | 1.125 | 0.955 |
| sp|Q9D1D4|TMEDA_MOUSE | Transmembrane emp24 domain-containing protein 10 OS=Mus musculus GN=Tmed10 PE=2 SV=1 | 28 | 1.224 | 0.936 | 0.956 |
| sp|P45376|ALDR_MOUSE | Aldose reductase OS=Mus musculus GN=Akr1b1 PE=1 SV=3 | 28 | 1.357 | 1.126 | 1.000 |
| sp|Q9CR16|PPID_MOUSE | Peptidyl-prolyl cis-trans isomerase D OS=Mus musculus GN=Ppid PE=1 SV=3 | 28 | 1.343 | 1.017 | 1.044 |
| sp|Q3TEA8|HP1B3_MOUSE | Heterochromatin protein 1-binding protein 3 OS=Mus musculus GN=Hp1bp3 PE=1 SV=1 | 27 | 1.087 | 1.110 | 0.648 |
| sp|P59242|CING_MOUSE | Cingulin OS=Mus musculus GN=Cgn PE=1 SV=1 | 27 | 0.810 | 0.769 | 0.694 |
| sp|Q62465|VAT1_MOUSE | Synaptic vesicle membrane protein VAT-1 homolog OS=Mus musculus GN=Vat1 PE=1 SV=3 | 27 | 1.007 | 0.976 | 0.704 |
| sp|P20060|HEXB_MOUSE | Beta-hexosaminidase subunit beta OS=Mus musculus GN=Hexb PE=2 SV=2 | 27 | 0.873 | 0.882 | 0.743 |
| sp|Q64133|AOFA_MOUSE | Amine oxidase [flavin-containing] A OS=Mus musculus GN=Maoa PE=1 SV=3 | 27 | 1.033 | 0.970 | 0.754 |
| sp|Q6PHZ2|KCC2D_MOUSE | Calcium/calmodulin-dependent protein kinase type II subunit delta OS=Mus musculus GN=Camk2d PE=1 SV=1 | 27 | 1.284 | 0.855 | 0.845 |
| sp|P42669|PURA_MOUSE | Transcriptional activator protein Pur-alpha OS=Mus musculus GN=Pura PE=1 SV=1 | 27 | 1.025 | 0.822 | 0.890 |
| sp|Q4VA53|PDS5B_MOUSE | Sister chromatid cohesion protein PDS5 homolog B OS=Mus musculus GN=Pds5b PE=1 SV=1 | 27 | 0.959 | 0.967 | 0.926 |
| sp|P56812|PDCD5_MOUSE | Programmed cell death protein 5 OS=Mus musculus GN=Pdcd5 PE=1 SV=3 | 27 | 0.985 | 1.091 | 0.927 |
| sp|O35685|NUDC_MOUSE | Nuclear migration protein nudC OS=Mus musculus GN=Nudc PE=1 SV=1 | 27 | 1.100 | 0.936 | 0.940 |
| sp|Q9QY23|PKP3_MOUSE | Plakophilin-3 OS=Mus musculus GN=Pkp3 PE=1 SV=2 | 27 | 0.957 | 0.772 | 0.953 |
| sp|Q7TSV4|PGM2_MOUSE | Phosphoglucomutase-2 OS=Mus musculus GN=Pgm2 PE=1 SV=1 | 27 | 0.896 | 1.045 | 0.955 |
| sp|Q791V5|MTCH2_MOUSE | Mitochondrial carrier homolog 2 OS=Mus musculus GN=Mtch2 PE=1 SV=1 | 27 | 0.890 | 1.000 | 0.962 |
| sp|P61202|CSN2_MOUSE | COP9 signalosome complex subunit 2 OS=Mus musculus GN=Cops2 PE=1 SV=1 | 27 | 1.418 | 1.025 | 1.016 |
| sp|P13745|GSTA1_MOUSE | Glutathione S-transferase A1 OS=Mus musculus GN=Gsta1 PE=1 SV=2 | 26 | 1.048 | 0.656 | 0.000 |
| sp|P35486|ODPA_MOUSE | Pyruvate dehydrogenase E1 component subunit alpha, somatic form, mitochondrial OS=Mus musculus GN=Pdha1 PE=1 SV=1 | 26 | 0.985 | 0.813 | 0.769 |
| sp|Q9QZ88|VPS29_MOUSE | Vacuolar protein sorting-associated protein 29 OS=Mus musculus GN=Vps29 PE=1 SV=1 | 26 | 0.946 | 0.843 | 0.873 |
| sp|Q8K4Z5|SF3A1_MOUSE | Splicing factor 3A subunit 1 OS=Mus musculus GN=Sf3a1 PE=1 SV=1 | 26 | 0.926 | 1.098 | 0.882 |
| sp|O35658|C1QBP_MOUSE | Complement component 1 Q subcomponent-binding protein, mitochondrial OS=Mus musculus GN=C1qbp PE=1 SV=1 | 26 | 1.128 | 0.917 | 0.886 |
| sp|Q6ZWV7|RL35_MOUSE | 60S ribosomal protein L35 OS=Mus musculus GN=Rpl35 PE=2 SV=1 | 26 | 1.141 | 1.022 | 0.890 |
| sp|Q9WTX5|SKP1_MOUSE | S-phase kinase-associated protein 1 OS=Mus musculus GN=Skp1 PE=1 SV=3 | 26 | 0.952 | 0.970 | 0.899 |
| sp|Q9R1T2|SAE1_MOUSE | SUMO-activating enzyme subunit 1 OS=Mus musculus GN=Sae1 PE=2 SV=1 | 26 | 1.201 | 0.888 | 0.947 |
| sp|O09061|PSB1_MOUSE | Proteasome subunit beta type-1 OS=Mus musculus GN=Psmb1 PE=1 SV=1 | 26 | 1.157 | 1.044 | 0.983 |
| sp|Q9DB05|SNAA_MOUSE | Alpha-soluble NSF attachment protein OS=Mus musculus GN=Napa PE=1 SV=1 | 26 | 1.053 | 1.000 | 0.989 |
| sp|Q8C0C7|SYFA_MOUSE | Phenylalanine--tRNA ligase alpha subunit OS=Mus musculus GN=Farsa PE=2 SV=1 | 26 | 1.061 | 1.112 | 1.010 |
| sp|P27661|H2AX_MOUSE | Histone H2A.x OS=Mus musculus GN=H2afx PE=1 SV=2 | 26 | InD | InD | 1.050 |
| sp|P23506|PIMT_MOUSE | Protein-L-isoaspartate(D-aspartate) O-methyltransferase OS=Mus musculus GN=Pcmt1 PE=1 SV=3 | 26 | 0.964 | 0.800 | 1.057 |
| sp|Q8C0I1|ADAS_MOUSE | Alkyldihydroxyacetonephosphate synthase, peroxisomal OS=Mus musculus GN=Agps PE=1 SV=1 | 26 | 0.822 | InD | 1.059 |
| sp|P14069|S10A6_MOUSE | Protein S100-A6 OS=Mus musculus GN=S100a6 PE=1 SV=3 | 25 | 1.292 | 0.858 | 0.000 |
| sp|Q91VM5|RMXL1_MOUSE | RNA binding motif protein, X-linked-like-1 OS=Mus musculus GN=Rbmxl1 PE=1 SV=1 | 25 | 1.115 | 1.160 | 0.406 |
| sp|P47754|CAZA2_MOUSE | F-actin-capping protein subunit alpha-2 OS=Mus musculus GN=Capza2 PE=1 SV=3 | 25 | 1.076 | 0.908 | 0.776 |
| sp|Q9D2G2|ODO2_MOUSE | Dihydrolipoyllysine-residue succinyltransferase component of 2-oxoglutarate dehydrogenase complex, mitochondrial OS=Mus musculus GN=Dlst PE=1 SV=1 | 25 | 1.197 | 0.819 | 0.814 |
| sp|Q6ZQ08|CNOT1_MOUSE | CCR4-NOT transcription complex subunit 1 OS=Mus musculus GN=Cnot1 PE=1 SV=2 | 25 | 1.069 | 0.759 | 0.817 |
| sp|Q8CHH9|SEPT8_MOUSE | Septin-8 OS=Mus musculus GN=Sept8 PE=1 SV=4 | 25 | 1.159 | 1.075 | 0.827 |
| sp|Q64010|CRK_MOUSE | Adapter molecule crk OS=Mus musculus GN=Crk PE=1 SV=1 | 25 | 0.941 | 0.828 | 0.856 |
| sp|Q8K4Z3|NNRE_MOUSE | NAD(P)H-hydrate epimerase OS=Mus musculus GN=Apoa1bp PE=1 SV=1 | 25 | 1.219 | 0.934 | 0.860 |
| sp|Q5SSW2|PSME4_MOUSE | Proteasome activator complex subunit 4 OS=Mus musculus GN=Psme4 PE=1 SV=1 | 25 | 0.968 | 1.208 | 0.897 |
| sp|P41105|RL28_MOUSE | 60S ribosomal protein L28 OS=Mus musculus GN=Rpl28 PE=1 SV=2 | 25 | 1.234 | 1.023 | 0.940 |
| sp|P61290|PSME3_MOUSE | Proteasome activator complex subunit 3 OS=Mus musculus GN=Psme3 PE=1 SV=1 | 25 | 1.034 | 0.905 | 0.970 |
| sp|Q9JJU8|SH3L1_MOUSE | SH3 domain-binding glutamic acid-rich-like protein OS=Mus musculus GN=Sh3bgrl PE=2 SV=1 | 25 | 1.008 | 1.036 | 0.992 |
| sp|Q6PDQ2|CHD4_MOUSE | Chromodomain-helicase-DNA-binding protein 4 OS=Mus musculus GN=Chd4 PE=1 SV=1 | 25 | 0.981 | 1.364 | 1.029 |
| sp|O08688|CAN5_MOUSE | Calpain-5 OS=Mus musculus GN=Capn5 PE=2 SV=1 | 25 | 0.867 | 1.509 | 1.101 |
| sp|P47856|GFPT1_MOUSE | Glutamine--fructose-6-phosphate aminotransferase [isomerizing] 1 OS=Mus musculus GN=Gfpt1 PE=1 SV=3 | 25 | 1.486 | 1.630 | 1.252 |
| sp|P51660|DHB4_MOUSE | Peroxisomal multifunctional enzyme type 2 OS=Mus musculus GN=Hsd17b4 PE=1 SV=3 | 25 | 1.346 | 0.531 | 1.277 |
| sp|P70333|HNRH2_MOUSE | Heterogeneous nuclear ribonucleoprotein H2 OS=Mus musculus GN=Hnrnph2 PE=1 SV=1 | 25 | 1.213 | InD | InD |
| sp|O35945|AL1A7_MOUSE | Aldehyde dehydrogenase, cytosolic 1 OS=Mus musculus GN=Aldh1a7 PE=2 SV=1 | 24 | 0.718 | 0.740 | 0.000 |
| sp|P16381|DDX3L_MOUSE | Putative ATP-dependent RNA helicase Pl10 OS=Mus musculus GN=D1Pas1 PE=1 SV=1 | 24 | InD | InD | 0.000 |
| sp|P10922|H10_MOUSE | Histone H1.0 OS=Mus musculus GN=H1f0 PE=2 SV=4 | 24 | 0.581 | 1.073 | 0.424 |
| sp|P18572|BASI_MOUSE | Basigin OS=Mus musculus GN=Bsg PE=1 SV=2 | 24 | 1.083 | 0.795 | 0.623 |
| sp|P62315|SMD1_MOUSE | Small nuclear ribonucleoprotein Sm D1 OS=Mus musculus GN=Snrpd1 PE=2 SV=1 | 24 | 1.095 | 0.960 | 0.708 |
| sp|P61161|ARP2_MOUSE | Actin-related protein 2 OS=Mus musculus GN=Actr2 PE=1 SV=1 | 24 | 1.077 | 0.933 | 0.825 |
| sp|Q9CY27|TECR_MOUSE | Very-long-chain enoyl-CoA reductase OS=Mus musculus GN=Tecr PE=1 SV=1 | 24 | 0.933 | InD | 0.863 |
| sp|P19783|COX41_MOUSE | Cytochrome c oxidase subunit 4 isoform 1, mitochondrial OS=Mus musculus GN=Cox4i1 PE=1 SV=2 | 24 | 0.940 | 1.730 | 0.867 |
| sp|Q9WUM4|COR1C_MOUSE | Coronin-1C OS=Mus musculus GN=Coro1c PE=1 SV=2 | 24 | 0.975 | 1.058 | 0.895 |
| sp|Q6ZWV3|RL10_MOUSE | 60S ribosomal protein L10 OS=Mus musculus GN=Rpl10 PE=2 SV=3 | 24 | 1.271 | 0.873 | 0.899 |
| sp|Q9CPW4|ARPC5_MOUSE | Actin-related protein 2/3 complex subunit 5 OS=Mus musculus GN=Arpc5 PE=2 SV=3 | 24 | 1.202 | 1.021 | 0.939 |
| sp|Q9DBD5|PELP1_MOUSE | Proline-, glutamic acid- and leucine-rich protein 1 OS=Mus musculus GN=Pelp1 PE=1 SV=2 | 24 | 0.960 | 1.078 | 1.000 |
| sp|Q61543|GSLG1_MOUSE | Golgi apparatus protein 1 OS=Mus musculus GN=Glg1 PE=1 SV=1 | 24 | 1.266 | 1.181 | 1.045 |
| sp|Q8VH51|RBM39_MOUSE | RNA-binding protein 39 OS=Mus musculus GN=Rbm39 PE=1 SV=2 | 24 | 1.208 | 1.124 | 1.094 |
| sp|P15379|CD44_MOUSE | CD44 antigen OS=Mus musculus GN=Cd44 PE=1 SV=3 | 24 | 2.085 | 1.571 | 1.151 |
| sp|Q00PI9|HNRL2_MOUSE | Heterogeneous nuclear ribonucleoprotein U-like protein 2 OS=Mus musculus GN=Hnrnpul2 PE=1 SV=2 | 24 | 1.381 | InD | 1.226 |
| sp|P70372|ELAV1_MOUSE | ELAV-like protein 1 OS=Mus musculus GN=Elavl1 PE=1 SV=2 | 24 | 1.313 | 1.067 | 1.320 |
| sp|Q9WUL7|ARL3_MOUSE | ADP-ribosylation factor-like protein 3 OS=Mus musculus GN=Arl3 PE=1 SV=1 | 24 | 1.110 | 1.292 | 1.640 |
| sp|P02535|K1C10_MOUSE | Keratin, type I cytoskeletal 10 OS=Mus musculus GN=Krt10 PE=1 SV=3 | 24 | 1.546 | 1.000 | 16.515 |
| sp|P53657|KPYR_MOUSE | Pyruvate kinase isozymes R/L OS=Mus musculus GN=Pklr PE=2 SV=1 | 23 | 1.079 | 0.899 | 0.000 |
| sp|Q61176|ARGI1_MOUSE | Arginase-1 OS=Mus musculus GN=Arg1 PE=1 SV=1 | 23 | 0.732 | InD | 0.651 |
| sp|Q62422|OSTF1_MOUSE | Osteoclast-stimulating factor 1 OS=Mus musculus GN=Ostf1 PE=1 SV=2 | 23 | 0.893 | 0.914 | 0.752 |
| sp|Q9JIW9|RALB_MOUSE | Ras-related protein Ral-B OS=Mus musculus GN=Ralb PE=2 SV=1 | 23 | 1.104 | 0.946 | 0.766 |
| sp|P35282|RAB21_MOUSE | Ras-related protein Rab-21 OS=Mus musculus GN=Rab21 PE=1 SV=4 | 23 | 1.153 | 0.882 | 0.796 |
| sp|Q64324|STXB2_MOUSE | Syntaxin-binding protein 2 OS=Mus musculus GN=Stxbp2 PE=2 SV=1 | 23 | 0.941 | 0.757 | 0.871 |
| sp|Q8BWZ3|NAA25_MOUSE | N-alpha-acetyltransferase 25, NatB auxiliary subunit OS=Mus musculus GN=Naa25 PE=1 SV=1 | 23 | 1.381 | 1.146 | 0.877 |
| sp|P57716|NICA_MOUSE | Nicastrin OS=Mus musculus GN=Ncstn PE=1 SV=3 | 23 | 0.973 | 0.777 | 0.903 |
| sp|P61514|RL37A_MOUSE | 60S ribosomal protein L37a OS=Mus musculus GN=Rpl37a PE=2 SV=2 | 23 | 1.183 | 1.111 | 0.907 |
| sp|Q9WV91|FPRP_MOUSE | Prostaglandin F2 receptor negative regulator OS=Mus musculus GN=Ptgfrn PE=1 SV=2 | 23 | 1.082 | 0.913 | 0.930 |
| sp|P34022|RANG_MOUSE | Ran-specific GTPase-activating protein OS=Mus musculus GN=Ranbp1 PE=1 SV=2 | 23 | 1.024 | 0.930 | 0.992 |
| sp|Q9DAR7|DCPS_MOUSE | m7GpppX diphosphatase OS=Mus musculus GN=Dcps PE=1 SV=1 | 23 | 1.000 | 0.000 | 1.020 |
| sp|Q9JII6|AK1A1_MOUSE | Alcohol dehydrogenase [NADP(+)] OS=Mus musculus GN=Akr1a1 PE=1 SV=3 | 23 | 0.919 | 0.731 | 1.067 |
| sp|P46061|RAGP1_MOUSE | Ran GTPase-activating protein 1 OS=Mus musculus GN=Rangap1 PE=1 SV=2 | 23 | 0.747 | 0.000 | 1.073 |
| sp|P47740|AL3A2_MOUSE | Fatty aldehyde dehydrogenase OS=Mus musculus GN=Aldh3a2 PE=2 SV=2 | 22 | 0.441 | 1.031 | 0.000 |
| sp|P24547|IMDH2_MOUSE | Inosine-5&#039;-monophosphate dehydrogenase 2 OS=Mus musculus GN=Impdh2 PE=1 SV=2 | 22 | 1.074 | 1.364 | 0.000 |
| sp|Q8BH04|PCKGM_MOUSE | Phosphoenolpyruvate carboxykinase [GTP], mitochondrial OS=Mus musculus GN=Pck2 PE=2 SV=1 | 22 | 1.095 | 1.054 | 0.671 |
| sp|Q60870|REEP5_MOUSE | Receptor expression-enhancing protein 5 OS=Mus musculus GN=Reep5 PE=1 SV=1 | 22 | 0.992 | 0.791 | 0.675 |
| sp|P51855|GSHB_MOUSE | Glutathione synthetase OS=Mus musculus GN=Gss PE=2 SV=1 | 22 | 0.724 | 0.946 | 0.685 |
| sp|Q01405|SC23A_MOUSE | Protein transport protein Sec23A OS=Mus musculus GN=Sec23a PE=1 SV=2 | 22 | 1.011 | 1.074 | 0.804 |
| sp|P54822|PUR8_MOUSE | Adenylosuccinate lyase OS=Mus musculus GN=Adsl PE=2 SV=2 | 22 | 0.892 | 0.873 | 0.813 |
| sp|Q8BH95|ECHM_MOUSE | Enoyl-CoA hydratase, mitochondrial OS=Mus musculus GN=Echs1 PE=1 SV=1 | 22 | 0.901 | 1.204 | 0.865 |
| sp|Q920E5|FPPS_MOUSE | Farnesyl pyrophosphate synthase OS=Mus musculus GN=Fdps PE=2 SV=1 | 22 | 1.154 | 1.308 | 0.875 |
| sp|O08997|ATOX1_MOUSE | Copper transport protein ATOX1 OS=Mus musculus GN=Atox1 PE=2 SV=1 | 22 | 0.624 | 0.968 | 0.901 |
| sp|P62858|RS28_MOUSE | 40S ribosomal protein S28 OS=Mus musculus GN=Rps28 PE=2 SV=1 | 22 | 1.049 | 0.964 | 0.905 |
| sp|O08795|GLU2B_MOUSE | Glucosidase 2 subunit beta OS=Mus musculus GN=Prkcsh PE=1 SV=1 | 22 | 1.031 | 1.127 | 0.913 |
| sp|Q9D7G0|PRPS1_MOUSE | Ribose-phosphate pyrophosphokinase 1 OS=Mus musculus GN=Prps1 PE=1 SV=4 | 22 | 1.027 | 1.270 | 0.924 |
| sp|Q99P88|NU155_MOUSE | Nuclear pore complex protein Nup155 OS=Mus musculus GN=Nup155 PE=2 SV=1 | 22 | 1.210 | 0.695 | 0.950 |
| sp|P48771|CX7A2_MOUSE | Cytochrome c oxidase subunit 7A2, mitochondrial OS=Mus musculus GN=Cox7a2 PE=1 SV=2 | 22 | 1.202 | 0.872 | 0.957 |
| sp|Q6P1F6|2ABA_MOUSE | Serine/threonine-protein phosphatase 2A 55 kDa regulatory subunit B alpha isoform OS=Mus musculus GN=Ppp2r2a PE=1 SV=1 | 22 | 1.242 | 0.904 | 1.006 |
| sp|P84099|RL19_MOUSE | 60S ribosomal protein L19 OS=Mus musculus GN=Rpl19 PE=1 SV=1 | 22 | 1.154 | 0.913 | 1.042 |
| sp|P17918|PCNA_MOUSE | Proliferating cell nuclear antigen OS=Mus musculus GN=Pcna PE=1 SV=2 | 22 | 1.078 | 0.990 | 1.113 |
| sp|O70503|DHB12_MOUSE | Estradiol 17-beta-dehydrogenase 12 OS=Mus musculus GN=Hsd17b12 PE=2 SV=1 | 22 | 1.205 | 0.992 | 1.257 |
| sp|P62748|HPCL1_MOUSE | Hippocalcin-like protein 1 OS=Mus musculus GN=Hpcal1 PE=2 SV=2 | 22 | 1.000 | 0.845 | 1.371 |
| sp|Q91ZW3|SMCA5_MOUSE | SWI/SNF-related matrix-associated actin-dependent regulator of chromatin subfamily A member 5 OS=Mus musculus GN=Smarca5 PE=1 SV=1 | 21 | 1.069 | 0.805 | 0.000 |
| sp|Q7TQH0|ATX2L_MOUSE | Ataxin-2-like protein OS=Mus musculus GN=Atxn2l PE=1 SV=1 | 21 | 1.151 | 0.989 | 0.000 |
| sp|P43276|H15_MOUSE | Histone H1.5 OS=Mus musculus GN=Hist1h1b PE=1 SV=2 | 21 | 1.000 | 1.872 | 0.310 |
| sp|P43275|H11_MOUSE | Histone H1.1 OS=Mus musculus GN=Hist1h1a PE=1 SV=2 | 21 | 0.000 | 0.185 | 0.438 |
| sp|Q61599|GDIR2_MOUSE | Rho GDP-dissociation inhibitor 2 OS=Mus musculus GN=Arhgdib PE=1 SV=3 | 21 | 0.718 | 0.786 | 0.539 |
| sp|D3Z7P3|GLSK_MOUSE | Glutaminase kidney isoform, mitochondrial OS=Mus musculus GN=Gls PE=1 SV=1 | 21 | 1.133 | 1.301 | 0.676 |
| sp|Q9CXY6|ILF2_MOUSE | Interleukin enhancer-binding factor 2 OS=Mus musculus GN=Ilf2 PE=1 SV=1 | 21 | 0.876 | 0.908 | 0.791 |
| sp|P09803|CADH1_MOUSE | Cadherin-1 OS=Mus musculus GN=Cdh1 PE=1 SV=1 | 21 | 0.868 | 3.640 | 0.793 |
| sp|O70492|SNX3_MOUSE | Sorting nexin-3 OS=Mus musculus GN=Snx3 PE=1 SV=3 | 21 | 0.957 | 0.937 | 0.820 |
| sp|Q8CIN4|PAK2_MOUSE | Serine/threonine-protein kinase PAK 2 OS=Mus musculus GN=Pak2 PE=1 SV=1 | 21 | 1.130 | 0.798 | 0.903 |
| sp|Q9CPU0|LGUL_MOUSE | Lactoylglutathione lyase OS=Mus musculus GN=Glo1 PE=1 SV=3 | 21 | 0.931 | 0.953 | 0.905 |
| sp|Q91WQ3|SYYC_MOUSE | Tyrosine--tRNA ligase, cytoplasmic OS=Mus musculus GN=Yars PE=2 SV=3 | 21 | 0.853 | 0.988 | 0.910 |
| sp|P30681|HMGB2_MOUSE | High mobility group protein B2 OS=Mus musculus GN=Hmgb2 PE=1 SV=3 | 21 | 0.880 | 0.936 | 0.930 |
| sp|Q3UDE2|TTL12_MOUSE | Tubulin--tyrosine ligase-like protein 12 OS=Mus musculus GN=Ttll12 PE=1 SV=1 | 21 | 1.066 | 1.047 | 0.955 |
| sp|P63005|LIS1_MOUSE | Platelet-activating factor acetylhydrolase IB subunit alpha OS=Mus musculus GN=Pafah1b1 PE=1 SV=2 | 21 | 1.069 | 0.810 | 0.964 |
| sp|Q9WTL2|RAB25_MOUSE | Ras-related protein Rab-25 OS=Mus musculus GN=Rab25 PE=1 SV=2 | 21 | 1.011 | 1.148 | 1.031 |
| sp|Q8VBW6|ULA1_MOUSE | NEDD8-activating enzyme E1 regulatory subunit OS=Mus musculus GN=Nae1 PE=2 SV=1 | 21 | 1.170 | 1.113 | 1.048 |
| sp|Q80UW8|RPAB1_MOUSE | DNA-directed RNA polymerases I, II, and III subunit RPABC1 OS=Mus musculus GN=Polr2e PE=2 SV=1 | 21 | 1.097 | 0.832 | 1.062 |
| sp|Q922Q4|P5CR2_MOUSE | Pyrroline-5-carboxylate reductase 2 OS=Mus musculus GN=Pycr2 PE=2 SV=1 | 21 | 1.299 | 0.911 | 1.076 |
| sp|Q91W50|CSDE1_MOUSE | Cold shock domain-containing protein E1 OS=Mus musculus GN=Csde1 PE=2 SV=1 | 21 | 1.205 | InD | 1.115 |
| sp|Q9WVK4|EHD1_MOUSE | EH domain-containing protein 1 OS=Mus musculus GN=Ehd1 PE=1 SV=1 | 21 | 1.467 | InD | 1.119 |
| sp|Q921H8|THIKA_MOUSE | 3-ketoacyl-CoA thiolase A, peroxisomal OS=Mus musculus GN=Acaa1a PE=2 SV=1 | 21 | 1.152 | 1.290 | 1.155 |
| sp|Q6Y7W8|PERQ2_MOUSE | PERQ amino acid-rich with GYF domain-containing protein 2 OS=Mus musculus GN=Gigyf2 PE=1 SV=2 | 21 | 1.037 | 1.032 | 1.172 |
| sp|P97494|GSH1_MOUSE | Glutamate--cysteine ligase catalytic subunit OS=Mus musculus GN=Gclc PE=2 SV=4 | 20 | 0.816 | 0.672 | 0.520 |
| sp|Q3UBX0|TM109_MOUSE | Transmembrane protein 109 OS=Mus musculus GN=Tmem109 PE=1 SV=2 | 20 | 0.896 | 0.833 | 0.629 |
| sp|Q9CPQ8|ATP5L_MOUSE | ATP synthase subunit g, mitochondrial OS=Mus musculus GN=Atp5l PE=1 SV=1 | 20 | 0.907 | 0.874 | 0.658 |
| sp|Q9QZ06|TOLIP_MOUSE | Toll-interacting protein OS=Mus musculus GN=Tollip PE=1 SV=1 | 20 | 1.023 | 0.902 | 0.675 |
| sp|Q61508|ECM1_MOUSE | Extracellular matrix protein 1 OS=Mus musculus GN=Ecm1 PE=1 SV=2 | 20 | 1.279 | 2.929 | 0.719 |
| sp|P63328|PP2BA_MOUSE | Serine/threonine-protein phosphatase 2B catalytic subunit alpha isoform OS=Mus musculus GN=Ppp3ca PE=1 SV=1 | 20 | 0.453 | 0.854 | 0.724 |
| sp|Q9CR57|RL14_MOUSE | 60S ribosomal protein L14 OS=Mus musculus GN=Rpl14 PE=2 SV=3 | 20 | 1.145 | 0.962 | 0.739 |
| sp|B2RXS4|PLXB2_MOUSE | Plexin-B2 OS=Mus musculus GN=Plxnb2 PE=1 SV=1 | 20 | 1.205 | InD | 0.770 |
| sp|Q99K01|PDXD1_MOUSE | Pyridoxal-dependent decarboxylase domain-containing protein 1 OS=Mus musculus GN=Pdxdc1 PE=1 SV=2 | 20 | 1.178 | 0.923 | 0.783 |
| sp|Q921Y0|MOB1A_MOUSE | MOB kinase activator 1A OS=Mus musculus GN=Mob1a PE=1 SV=3 | 20 | 1.438 | 0.940 | 0.835 |
| sp|P63323|RS12_MOUSE | 40S ribosomal protein S12 OS=Mus musculus GN=Rps12 PE=1 SV=2 | 20 | 1.176 | 1.035 | 0.848 |
| sp|Q07813|BAX_MOUSE | Apoptosis regulator BAX OS=Mus musculus GN=Bax PE=1 SV=1 | 20 | 0.801 | 0.919 | 0.857 |
| sp|Q9DAS9|GBG12_MOUSE | Guanine nucleotide-binding protein G(I)/G(S)/G(O) subunit gamma-12 OS=Mus musculus GN=Gng12 PE=1 SV=3 | 20 | 1.051 | 0.993 | 0.859 |
| sp|Q8C7R4|UBA6_MOUSE | Ubiquitin-like modifier-activating enzyme 6 OS=Mus musculus GN=Uba6 PE=1 SV=1 | 20 | 1.762 | InD | 0.918 |
| sp|O08583|THOC4_MOUSE | THO complex subunit 4 OS=Mus musculus GN=Alyref PE=1 SV=3 | 20 | 0.944 | 0.906 | 0.924 |
| sp|Q8BRF7|SCFD1_MOUSE | Sec1 family domain-containing protein 1 OS=Mus musculus GN=Scfd1 PE=2 SV=1 | 20 | 1.163 | 1.137 | 1.019 |
| sp|Q9JMH6|TRXR1_MOUSE | Thioredoxin reductase 1, cytoplasmic OS=Mus musculus GN=Txnrd1 PE=1 SV=3 | 20 | 1.292 | 1.202 | 1.022 |
| sp|P58044|IDI1_MOUSE | Isopentenyl-diphosphate Delta-isomerase 1 OS=Mus musculus GN=Idi1 PE=2 SV=1 | 20 | 1.455 | 1.237 | 1.029 |
| sp|Q9QXB9|DRG2_MOUSE | Developmentally-regulated GTP-binding protein 2 OS=Mus musculus GN=Drg2 PE=1 SV=1 | 20 | 1.147 | 0.981 | 1.040 |
| sp|Q810A7|DDX42_MOUSE | ATP-dependent RNA helicase DDX42 OS=Mus musculus GN=Ddx42 PE=1 SV=3 | 20 | 1.466 | InD | 1.076 |
| sp|Q9CQI6|COTL1_MOUSE | Coactosin-like protein OS=Mus musculus GN=Cotl1 PE=1 SV=3 | 20 | 1.092 | 0.888 | 1.126 |
| sp|P81117|NUCB2_MOUSE | Nucleobindin-2 OS=Mus musculus GN=Nucb2 PE=1 SV=2 | 20 | 1.693 | 1.287 | 1.189 |
| sp|P60229|EIF3E_MOUSE | Eukaryotic translation initiation factor 3 subunit E OS=Mus musculus GN=Eif3e PE=1 SV=1 | 20 | 0.971 | 0.939 | 1.569 |
| sp|Q501J6|DDX17_MOUSE | Probable ATP-dependent RNA helicase DDX17 OS=Mus musculus GN=Ddx17 PE=2 SV=1 | 19 | InD | 0.000 | 0.000 |
| sp|Q9CQW1|YKT6_MOUSE | Synaptobrevin homolog YKT6 OS=Mus musculus GN=Ykt6 PE=2 SV=1 | 19 | 1.054 | 0.890 | 0.000 |
| sp|P40240|CD9_MOUSE | CD9 antigen OS=Mus musculus GN=Cd9 PE=1 SV=2 | 19 | 0.786 | 0.942 | 0.000 |
| sp|Q61035|SYHC_MOUSE | Histidine--tRNA ligase, cytoplasmic OS=Mus musculus GN=Hars PE=2 SV=2 | 19 | 1.146 | 1.020 | 0.000 |
| sp|P62317|SMD2_MOUSE | Small nuclear ribonucleoprotein Sm D2 OS=Mus musculus GN=Snrpd2 PE=2 SV=1 | 19 | 1.111 | 0.970 | 0.579 |
| sp|Q6PDG5|SMRC2_MOUSE | SWI/SNF complex subunit SMARCC2 OS=Mus musculus GN=Smarcc2 PE=1 SV=2 | 19 | 0.918 | 1.075 | 0.754 |
| sp|P70335|ROCK1_MOUSE | Rho-associated protein kinase 1 OS=Mus musculus GN=Rock1 PE=1 SV=1 | 19 | 0.911 | 0.921 | 0.770 |
| sp|Q9DCW4|ETFB_MOUSE | Electron transfer flavoprotein subunit beta OS=Mus musculus GN=Etfb PE=1 SV=3 | 19 | 1.092 | 0.877 | 0.804 |
| sp|Q80TL7|MON2_MOUSE | Protein MON2 homolog OS=Mus musculus GN=Mon2 PE=2 SV=2 | 19 | 1.096 | 0.841 | 0.811 |
| sp|Q61074|PPM1G_MOUSE | Protein phosphatase 1G OS=Mus musculus GN=Ppm1g PE=2 SV=3 | 19 | 1.304 | 1.216 | 0.816 |
| sp|Q9DC16|ERGI1_MOUSE | Endoplasmic reticulum-Golgi intermediate compartment protein 1 OS=Mus musculus GN=Ergic1 PE=1 SV=1 | 19 | 1.183 | 0.942 | 0.833 |
| sp|P97823|LYPA1_MOUSE | Acyl-protein thioesterase 1 OS=Mus musculus GN=Lypla1 PE=1 SV=1 | 19 | 0.891 | 0.839 | 0.865 |
| sp|P35279|RAB6A_MOUSE | Ras-related protein Rab-6A OS=Mus musculus GN=Rab6a PE=1 SV=4 | 19 | 1.297 | 1.192 | 0.867 |
| sp|P70349|HINT1_MOUSE | Histidine triad nucleotide-binding protein 1 OS=Mus musculus GN=Hint1 PE=1 SV=3 | 19 | 1.022 | 0.937 | 0.868 |
| sp|P70441|NHRF1_MOUSE | Na(+)/H(+) exchange regulatory cofactor NHE-RF1 OS=Mus musculus GN=Slc9a3r1 PE=1 SV=3 | 19 | 2.118 | 0.800 | 0.897 |
| sp|Q9D8B3|CHM4B_MOUSE | Charged multivesicular body protein 4b OS=Mus musculus GN=Chmp4b PE=2 SV=2 | 19 | 1.052 | 1.403 | 0.899 |
| sp|P47963|RL13_MOUSE | 60S ribosomal protein L13 OS=Mus musculus GN=Rpl13 PE=2 SV=3 | 19 | 1.322 | 1.027 | 0.927 |
| sp|Q9QXT0|CNPY2_MOUSE | Protein canopy homolog 2 OS=Mus musculus GN=Cnpy2 PE=2 SV=1 | 19 | 1.117 | 0.902 | 0.927 |
| sp|Q60737|CSK21_MOUSE | Casein kinase II subunit alpha OS=Mus musculus GN=Csnk2a1 PE=1 SV=2 | 19 | 0.784 | 0.952 | 0.936 |
| sp|Q99JH8|ERD21_MOUSE | ER lumen protein retaining receptor 1 OS=Mus musculus GN=Kdelr1 PE=2 SV=1 | 19 | 0.936 | InD | 0.955 |
| sp|Q9CQM2|ERD22_MOUSE | ER lumen protein retaining receptor 2 OS=Mus musculus GN=Kdelr2 PE=2 SV=1 | 19 | 0.942 | InD | 0.955 |
| sp|P57784|RU2A_MOUSE | U2 small nuclear ribonucleoprotein A&#039; OS=Mus musculus GN=Snrpa1 PE=1 SV=2 | 19 | 1.106 | 1.030 | 0.957 |
| sp|P70388|RAD50_MOUSE | DNA repair protein RAD50 OS=Mus musculus GN=Rad50 PE=1 SV=1 | 19 | 0.873 | 0.905 | 0.975 |
| sp|Q99MR6|SRRT_MOUSE | Serrate RNA effector molecule homolog OS=Mus musculus GN=Srrt PE=1 SV=1 | 19 | 0.935 | 0.974 | 0.992 |
| sp|Q9EST5|AN32B_MOUSE | Acidic leucine-rich nuclear phosphoprotein 32 family member B OS=Mus musculus GN=Anp32b PE=1 SV=1 | 19 | 0.893 | 0.942 | 1.023 |
| sp|P32020|NLTP_MOUSE | Non-specific lipid-transfer protein OS=Mus musculus GN=Scp2 PE=1 SV=3 | 19 | 1.052 | 1.032 | 1.032 |
| sp|Q9QWR8|NAGAB_MOUSE | Alpha-N-acetylgalactosaminidase OS=Mus musculus GN=Naga PE=2 SV=2 | 19 | InD | 0.909 | 1.041 |
| sp|Q8K0C4|CP51A_MOUSE | Lanosterol 14-alpha demethylase OS=Mus musculus GN=Cyp51a1 PE=2 SV=1 | 19 | 1.306 | 1.029 | 1.042 |
| sp|Q91X52|DCXR_MOUSE | L-xylulose reductase OS=Mus musculus GN=Dcxr PE=2 SV=2 | 19 | 0.000 | 1.228 | 1.059 |
| sp|Q6NV83|SR140_MOUSE | U2 snRNP-associated SURP motif-containing protein OS=Mus musculus GN=U2surp PE=1 SV=3 | 19 | 1.577 | 0.944 | 1.192 |
| sp|Q9D5V5|CUL5_MOUSE | Cullin-5 OS=Mus musculus GN=Cul5 PE=1 SV=3 | 19 | 0.832 | 0.592 | 1.458 |
| sp|O08579|EMD_MOUSE | Emerin OS=Mus musculus GN=Emd PE=1 SV=1 | 19 | 1.290 | 1.021 | InD |
| sp|Q6PB44|PTN23_MOUSE | Tyrosine-protein phosphatase non-receptor type 23 OS=Mus musculus GN=Ptpn23 PE=1 SV=2 | 19 | 1.358 | 1.195 | InD |
| sp|Q5FWK3|RHG01_MOUSE | Rho GTPase-activating protein 1 OS=Mus musculus GN=Arhgap1 PE=1 SV=1 | 18 | 1.035 | 0.904 | 0.000 |
| sp|Q61503|5NTD_MOUSE | 5&#039;-nucleotidase OS=Mus musculus GN=Nt5e PE=1 SV=2 | 18 | 0.837 | 0.419 | 0.286 |
| sp|Q9WTP7|KAD3_MOUSE | GTP:AMP phosphotransferase, mitochondrial OS=Mus musculus GN=Ak3 PE=1 SV=3 | 18 | 0.747 | 1.014 | 0.592 |
| sp|Q9JLV1|BAG3_MOUSE | BAG family molecular chaperone regulator 3 OS=Mus musculus GN=Bag3 PE=1 SV=2 | 18 | 1.078 | 0.582 | 0.691 |
| sp|Q61545|EWS_MOUSE | RNA-binding protein EWS OS=Mus musculus GN=Ewsr1 PE=1 SV=2 | 18 | 0.779 | 0.672 | 0.759 |
| sp|Q9DCL9|PUR6_MOUSE | Multifunctional protein ADE2 OS=Mus musculus GN=Paics PE=1 SV=4 | 18 | 0.937 | 1.050 | 0.770 |
| sp|Q9Z1D1|EIF3G_MOUSE | Eukaryotic translation initiation factor 3 subunit G OS=Mus musculus GN=Eif3g PE=1 SV=2 | 18 | 1.527 | 0.979 | 0.800 |
| sp|Q9Z1R2|BAG6_MOUSE | Large proline-rich protein BAG6 OS=Mus musculus GN=Bag6 PE=1 SV=1 | 18 | 1.195 | 0.000 | 0.810 |
| sp|P63085|MK01_MOUSE | Mitogen-activated protein kinase 1 OS=Mus musculus GN=Mapk1 PE=1 SV=3 | 18 | 1.374 | 0.891 | 0.824 |
| sp|O70194|EIF3D_MOUSE | Eukaryotic translation initiation factor 3 subunit D OS=Mus musculus GN=Eif3d PE=1 SV=2 | 18 | 1.032 | 1.061 | 0.825 |
| sp|Q9D1J3|SARNP_MOUSE | SAP domain-containing ribonucleoprotein OS=Mus musculus GN=Sarnp PE=1 SV=3 | 18 | 1.000 | 0.991 | 0.870 |
| sp|P62754|RS6_MOUSE | 40S ribosomal protein S6 OS=Mus musculus GN=Rps6 PE=1 SV=1 | 18 | 1.162 | 0.987 | 0.872 |
| sp|Q63932|MP2K2_MOUSE | Dual specificity mitogen-activated protein kinase kinase 2 OS=Mus musculus GN=Map2k2 PE=1 SV=2 | 18 | 1.075 | 0.000 | 0.878 |
| sp|Q9DCT2|NDUS3_MOUSE | NADH dehydrogenase [ubiquinone] iron-sulfur protein 3, mitochondrial OS=Mus musculus GN=Ndufs3 PE=1 SV=2 | 18 | 0.000 | 0.862 | 0.892 |
| sp|Q8BK64|AHSA1_MOUSE | Activator of 90 kDa heat shock protein ATPase homolog 1 OS=Mus musculus GN=Ahsa1 PE=2 SV=2 | 18 | 1.113 | 1.000 | 0.897 |
| sp|Q64191|ASPG_MOUSE | N(4)-(beta-N-acetylglucosaminyl)-L-asparaginase OS=Mus musculus GN=Aga PE=2 SV=1 | 18 | 1.058 | 0.973 | 0.909 |
| sp|Q9CZY3|UB2V1_MOUSE | Ubiquitin-conjugating enzyme E2 variant 1 OS=Mus musculus GN=Ube2v1 PE=1 SV=1 | 18 | 1.159 | 0.988 | 0.915 |
| sp|Q9DBL7|COASY_MOUSE | Bifunctional coenzyme A synthase OS=Mus musculus GN=Coasy PE=1 SV=2 | 18 | 0.000 | 0.844 | 0.932 |
| sp|P62331|ARF6_MOUSE | ADP-ribosylation factor 6 OS=Mus musculus GN=Arf6 PE=1 SV=2 | 18 | 0.941 | 1.162 | 0.981 |
| sp|Q8CFI7|RPB2_MOUSE | DNA-directed RNA polymerase II subunit RPB2 OS=Mus musculus GN=Polr2b PE=2 SV=2 | 18 | 0.783 | 0.915 | 1.037 |
| sp|Q8VED9|LEGLA_MOUSE | Galectin-related protein A OS=Mus musculus GN=Lgalsla PE=1 SV=1 | 18 | 1.679 | 0.796 | 1.052 |
| sp|Q60973|RBBP7_MOUSE | Histone-binding protein RBBP7 OS=Mus musculus GN=Rbbp7 PE=1 SV=1 | 18 | 1.200 | InD | 1.088 |
| sp|Q8K411|PREP_MOUSE | Presequence protease, mitochondrial OS=Mus musculus GN=Pitrm1 PE=2 SV=1 | 18 | 1.008 | InD | 1.152 |
| sp|P49935|CATH_MOUSE | Pro-cathepsin H OS=Mus musculus GN=Ctsh PE=2 SV=2 | 18 | 1.015 | 1.071 | 1.164 |
| sp|Q9CXI5|MANF_MOUSE | Mesencephalic astrocyte-derived neurotrophic factor OS=Mus musculus GN=Manf PE=1 SV=1 | 18 | 1.294 | 1.268 | 1.195 |
| sp|Q9JKY5|HIP1R_MOUSE | Huntingtin-interacting protein 1-related protein OS=Mus musculus GN=Hip1r PE=1 SV=2 | 18 | 1.162 | 1.356 | 1.547 |
| sp|Q8BKG3|PTK7_MOUSE | Inactive tyrosine-protein kinase 7 OS=Mus musculus GN=Ptk7 PE=1 SV=1 | 18 | 0.857 | 0.761 | InD |
| sp|Q9CQ48|NUDC2_MOUSE | NudC domain-containing protein 2 OS=Mus musculus GN=Nudcd2 PE=1 SV=1 | 18 | 1.152 | 0.973 | InD |
| sp|Q8VBV7|CSN8_MOUSE | COP9 signalosome complex subunit 8 OS=Mus musculus GN=Cops8 PE=1 SV=1 | 17 | 1.190 | 1.230 | 0.518 |
| sp|Q61792|LASP1_MOUSE | LIM and SH3 domain protein 1 OS=Mus musculus GN=Lasp1 PE=1 SV=1 | 17 | 0.888 | 0.970 | 0.667 |
| sp|Q61425|HCDH_MOUSE | Hydroxyacyl-coenzyme A dehydrogenase, mitochondrial OS=Mus musculus GN=Hadh PE=1 SV=2 | 17 | 0.992 | 0.912 | 0.693 |
| sp|P59017|B2L13_MOUSE | Bcl-2-like protein 13 OS=Mus musculus GN=Bcl2l13 PE=1 SV=2 | 17 | 0.000 | 0.900 | 0.714 |
| sp|P62281|RS11_MOUSE | 40S ribosomal protein S11 OS=Mus musculus GN=Rps11 PE=2 SV=3 | 17 | 1.034 | 0.866 | 0.744 |
| sp|Q8BH64|EHD2_MOUSE | EH domain-containing protein 2 OS=Mus musculus GN=Ehd2 PE=1 SV=1 | 17 | 0.000 | 0.644 | 0.763 |
| sp|P53810|PIPNA_MOUSE | Phosphatidylinositol transfer protein alpha isoform OS=Mus musculus GN=Pitpna PE=1 SV=2 | 17 | 1.102 | 0.843 | 0.796 |
| sp|P51432|PLCB3_MOUSE | 1-phosphatidylinositol 4,5-bisphosphate phosphodiesterase beta-3 OS=Mus musculus GN=Plcb3 PE=2 SV=2 | 17 | 1.196 | 0.961 | 0.813 |
| sp|Q8BK67|RCC2_MOUSE | Protein RCC2 OS=Mus musculus GN=Rcc2 PE=2 SV=1 | 17 | 0.000 | 0.836 | 0.886 |
| sp|Q9JIF0|ANM1_MOUSE | Protein arginine N-methyltransferase 1 OS=Mus musculus GN=Prmt1 PE=1 SV=1 | 17 | 0.981 | 1.022 | 0.894 |
| sp|Q9CPQ1|COX6C_MOUSE | Cytochrome c oxidase subunit 6C OS=Mus musculus GN=Cox6c PE=1 SV=3 | 17 | 1.119 | 0.921 | 0.902 |
| sp|Q9DCJ5|NDUA8_MOUSE | NADH dehydrogenase [ubiquinone] 1 alpha subcomplex subunit 8 OS=Mus musculus GN=Ndufa8 PE=1 SV=3 | 17 | 0.881 | 0.814 | 0.926 |
| sp|Q9JHW2|NIT2_MOUSE | Omega-amidase NIT2 OS=Mus musculus GN=Nit2 PE=1 SV=1 | 17 | 1.059 | InD | 0.953 |
| sp|P62075|TIM13_MOUSE | Mitochondrial import inner membrane translocase subunit Tim13 OS=Mus musculus GN=Timm13 PE=1 SV=1 | 17 | 1.124 | 1.019 | 0.963 |
| sp|Q9DC69|NDUA9_MOUSE | NADH dehydrogenase [ubiquinone] 1 alpha subcomplex subunit 9, mitochondrial OS=Mus musculus GN=Ndufa9 PE=1 SV=2 | 17 | 1.064 | 0.837 | 0.966 |
| sp|P22907|HEM3_MOUSE | Porphobilinogen deaminase OS=Mus musculus GN=Hmbs PE=2 SV=2 | 17 | 1.127 | 1.016 | 1.042 |
| sp|Q9D0T1|NH2L1_MOUSE | NHP2-like protein 1 OS=Mus musculus GN=Nhp2l1 PE=2 SV=4 | 17 | 1.017 | 1.178 | 1.065 |
| sp|Q80UM3|NAA15_MOUSE | N-alpha-acetyltransferase 15, NatA auxiliary subunit OS=Mus musculus GN=Naa15 PE=1 SV=1 | 17 | 1.141 | 0.951 | 1.080 |
| sp|Q80X82|SYMPK_MOUSE | Symplekin OS=Mus musculus GN=Sympk PE=1 SV=1 | 17 | 0.855 | 0.930 | 1.170 |
| sp|Q60972|RBBP4_MOUSE | Histone-binding protein RBBP4 OS=Mus musculus GN=Rbbp4 PE=1 SV=5 | 17 | 1.102 | 0.853 | InD |
| sp|Q9Z1T1|AP3B1_MOUSE | AP-3 complex subunit beta-1 OS=Mus musculus GN=Ap3b1 PE=1 SV=2 | 17 | 0.949 | 0.949 | InD |
| sp|P63168|DYL1_MOUSE | Dynein light chain 1, cytoplasmic OS=Mus musculus GN=Dynll1 PE=1 SV=1 | 16 | 0.891 | 0.870 | 0.000 |
| sp|P29595|NEDD8_MOUSE | NEDD8 OS=Mus musculus GN=Nedd8 PE=1 SV=2 | 16 | 1.096 | 1.018 | 0.000 |
| sp|B2RY56|RBM25_MOUSE | RNA-binding protein 25 OS=Mus musculus GN=Rbm25 PE=1 SV=1 | 16 | 1.241 | InD | 0.000 |
| sp|Q6A4J8|UBP7_MOUSE | Ubiquitin carboxyl-terminal hydrolase 7 OS=Mus musculus GN=Usp7 PE=1 SV=1 | 16 | 1.016 | 0.941 | 0.292 |
| sp|Q9Z204|HNRPC_MOUSE | Heterogeneous nuclear ribonucleoproteins C1/C2 OS=Mus musculus GN=Hnrnpc PE=1 SV=1 | 16 | InD | 0.819 | 0.378 |
| sp|O89086|RBM3_MOUSE | Putative RNA-binding protein 3 OS=Mus musculus GN=Rbm3 PE=1 SV=1 | 16 | 0.916 | 0.733 | 0.559 |
| sp|Q6IFZ6|K2C1B_MOUSE | Keratin, type II cytoskeletal 1b OS=Mus musculus GN=Krt77 PE=1 SV=1 | 16 | 1.379 | 1.020 | 0.595 |
| sp|Q8BP67|RL24_MOUSE | 60S ribosomal protein L24 OS=Mus musculus GN=Rpl24 PE=2 SV=2 | 16 | 1.056 | 0.844 | 0.634 |
| sp|O35902|DSG3_MOUSE | Desmoglein-3 OS=Mus musculus GN=Dsg3 PE=2 SV=1 | 16 | 0.571 | 0.000 | 0.663 |
| sp|P42227|STAT3_MOUSE | Signal transducer and activator of transcription 3 OS=Mus musculus GN=Stat3 PE=1 SV=2 | 16 | 0.811 | 0.000 | 0.670 |
| sp|Q9R190|MTA2_MOUSE | Metastasis-associated protein MTA2 OS=Mus musculus GN=Mta2 PE=1 SV=1 | 16 | 0.971 | 0.729 | 0.724 |
| sp|P27048|RSMB_MOUSE | Small nuclear ribonucleoprotein-associated protein B OS=Mus musculus GN=Snrpb PE=1 SV=1 | 16 | 1.043 | 0.979 | 0.778 |
| sp|P63163|RSMN_MOUSE | Small nuclear ribonucleoprotein-associated protein N OS=Mus musculus GN=Snrpn PE=2 SV=1 | 16 | 1.043 | 0.979 | 0.778 |
| sp|Q9DB15|RM12_MOUSE | 39S ribosomal protein L12, mitochondrial OS=Mus musculus GN=Mrpl12 PE=1 SV=2 | 16 | 0.968 | 0.964 | 0.788 |
| sp|P63087|PP1G_MOUSE | Serine/threonine-protein phosphatase PP1-gamma catalytic subunit OS=Mus musculus GN=Ppp1cc PE=1 SV=1 | 16 | InD | InD | 0.792 |
| sp|Q99LF4|RTCB_MOUSE | tRNA-splicing ligase RtcB homolog OS=Mus musculus GN=D10Wsu52e PE=2 SV=1 | 16 | 0.000 | 1.198 | 0.819 |
| sp|Q9CT10|RANB3_MOUSE | Ran-binding protein 3 OS=Mus musculus GN=Ranbp3 PE=1 SV=2 | 16 | 1.228 | 1.138 | 0.828 |
| sp|O35900|LSM2_MOUSE | U6 snRNA-associated Sm-like protein LSm2 OS=Mus musculus GN=Lsm2 PE=2 SV=1 | 16 | 1.109 | 1.086 | 0.833 |
| sp|Q8BPB0|MOB1B_MOUSE | MOB kinase activator 1B OS=Mus musculus GN=Mob1b PE=1 SV=3 | 16 | 0.000 | 0.947 | 0.835 |
| sp|Q99KK2|NEUA_MOUSE | N-acylneuraminate cytidylyltransferase OS=Mus musculus GN=Cmas PE=1 SV=2 | 16 | 1.088 | 1.046 | 0.836 |
| sp|P62880|GBB2_MOUSE | Guanine nucleotide-binding protein G(I)/G(S)/G(T) subunit beta-2 OS=Mus musculus GN=Gnb2 PE=1 SV=3 | 16 | 0.949 | 0.900 | 0.843 |
| sp|P62874|GBB1_MOUSE | Guanine nucleotide-binding protein G(I)/G(S)/G(T) subunit beta-1 OS=Mus musculus GN=Gnb1 PE=1 SV=3 | 16 | 0.949 | 0.906 | 0.843 |
| sp|O55142|RL35A_MOUSE | 60S ribosomal protein L35a OS=Mus musculus GN=Rpl35a PE=2 SV=2 | 16 | 1.033 | 0.634 | 0.859 |
| sp|Q6PHN9|RAB35_MOUSE | Ras-related protein Rab-35 OS=Mus musculus GN=Rab35 PE=1 SV=1 | 16 | InD | 0.936 | 0.883 |
| sp|Q6P2B1|TNPO3_MOUSE | Transportin-3 OS=Mus musculus GN=Tnpo3 PE=1 SV=1 | 16 | 0.902 | 0.663 | 0.883 |
| sp|P86048|RL10L_MOUSE | 60S ribosomal protein L10-like OS=Mus musculus GN=Rpl10l PE=2 SV=1 | 16 | InD | 0.873 | 0.899 |
| sp|Q9D6J6|NDUV2_MOUSE | NADH dehydrogenase [ubiquinone] flavoprotein 2, mitochondrial OS=Mus musculus GN=Ndufv2 PE=1 SV=2 | 16 | 1.088 | 0.970 | 0.927 |
| sp|Q9ERG0|LIMA1_MOUSE | LIM domain and actin-binding protein 1 OS=Mus musculus GN=Lima1 PE=1 SV=3 | 16 | 1.360 | 1.422 | 0.929 |
| sp|Q91VH6|MEMO1_MOUSE | Protein MEMO1 OS=Mus musculus GN=Memo1 PE=1 SV=1 | 16 | 1.063 | 0.863 | 0.948 |
| sp|Q8C854|MYEF2_MOUSE | Myelin expression factor 2 OS=Mus musculus GN=Myef2 PE=1 SV=1 | 16 | 1.040 | 1.000 | 0.951 |
| sp|Q9DBG6|RPN2_MOUSE | Dolichyl-diphosphooligosaccharide--protein glycosyltransferase subunit 2 OS=Mus musculus GN=Rpn2 PE=2 SV=1 | 16 | 1.425 | 0.664 | 0.978 |
| sp|Q8VI75|IPO4_MOUSE | Importin-4 OS=Mus musculus GN=Ipo4 PE=1 SV=1 | 16 | 1.156 | 1.131 | 0.990 |
| sp|Q9R0Q7|TEBP_MOUSE | Prostaglandin E synthase 3 OS=Mus musculus GN=Ptges3 PE=1 SV=1 | 16 | 1.165 | 0.977 | 0.992 |
| sp|Q07113|MPRI_MOUSE | Cation-independent mannose-6-phosphate receptor OS=Mus musculus GN=Igf2r PE=1 SV=1 | 16 | 0.663 | 0.866 | 1.038 |
| sp|Q99L47|F10A1_MOUSE | Hsc70-interacting protein OS=Mus musculus GN=St13 PE=2 SV=1 | 16 | 1.165 | 1.067 | 1.072 |
| sp|Q9QUH0|GLRX1_MOUSE | Glutaredoxin-1 OS=Mus musculus GN=Glrx PE=1 SV=3 | 16 | 1.301 | 1.198 | 1.083 |
| sp|Q9JK38|GNA1_MOUSE | Glucosamine 6-phosphate N-acetyltransferase OS=Mus musculus GN=Gnpnat1 PE=2 SV=1 | 16 | 0.900 | 1.107 | 1.170 |
| sp|Q9CQL1|MGN2_MOUSE | Protein mago nashi homolog 2 OS=Mus musculus GN=Magohb PE=2 SV=1 | 16 | 0.987 | InD | 1.210 |
| sp|P61327|MGN_MOUSE | Protein mago nashi homolog OS=Mus musculus GN=Magoh PE=2 SV=1 | 16 | 1.000 | InD | 1.210 |
| sp|Q9R118|HTRA1_MOUSE | Serine protease HTRA1 OS=Mus musculus GN=Htra1 PE=1 SV=2 | 16 | 0.851 | 1.290 | 1.375 |
| sp|Q91WK1|SPRY4_MOUSE | SPRY domain-containing protein 4 OS=Mus musculus GN=Spryd4 PE=2 SV=1 | 16 | 0.691 | 1.362 | 1.505 |
| sp|O35215|DOPD_MOUSE | D-dopachrome decarboxylase OS=Mus musculus GN=Ddt PE=1 SV=3 | 16 | 0.894 | 0.555 | InD |
| sp|P61082|UBC12_MOUSE | NEDD8-conjugating enzyme Ubc12 OS=Mus musculus GN=Ube2m PE=2 SV=1 | 16 | InD | 1.153 | InD |
| sp|P59325|IF5_MOUSE | Eukaryotic translation initiation factor 5 OS=Mus musculus GN=Eif5 PE=1 SV=1 | 15 | 1.477 | 0.897 | 0.000 |
| sp|Q6ZQ58|LARP1_MOUSE | La-related protein 1 OS=Mus musculus GN=Larp1 PE=1 SV=2 | 15 | 1.113 | 1.075 | 0.000 |
| sp|P70398|USP9X_MOUSE | Probable ubiquitin carboxyl-terminal hydrolase FAF-X OS=Mus musculus GN=Usp9x PE=1 SV=2 | 15 | 1.648 | 1.111 | 0.000 |
| sp|Q07076|ANXA7_MOUSE | Annexin A7 OS=Mus musculus GN=Anxa7 PE=2 SV=2 | 15 | 1.133 | 0.843 | 0.449 |
| sp|Q8BJ71|NUP93_MOUSE | Nuclear pore complex protein Nup93 OS=Mus musculus GN=Nup93 PE=2 SV=1 | 15 | 1.200 | 0.783 | 0.549 |
| sp|Q63810|CANB1_MOUSE | Calcineurin subunit B type 1 OS=Mus musculus GN=Ppp3r1 PE=1 SV=3 | 15 | 1.124 | 1.234 | 0.580 |
| sp|P35293|RAB18_MOUSE | Ras-related protein Rab-18 OS=Mus musculus GN=Rab18 PE=2 SV=2 | 15 | 1.736 | 1.078 | 0.671 |
| sp|O88746|TOM1_MOUSE | Target of Myb protein 1 OS=Mus musculus GN=Tom1 PE=1 SV=1 | 15 | 0.886 | 0.798 | 0.689 |
| sp|Q8VE70|PDC10_MOUSE | Programmed cell death protein 10 OS=Mus musculus GN=Pdcd10 PE=1 SV=1 | 15 | 0.980 | 0.896 | 0.766 |
| sp|Q99KR3|LACB2_MOUSE | Beta-lactamase-like protein 2 OS=Mus musculus GN=Lactb2 PE=1 SV=1 | 15 | 1.306 | 0.947 | 0.780 |
| sp|Q3UZ39|LRRF1_MOUSE | Leucine-rich repeat flightless-interacting protein 1 OS=Mus musculus GN=Lrrfip1 PE=1 SV=2 | 15 | 1.089 | 0.801 | 0.785 |
| sp|Q8BKZ9|ODPX_MOUSE | Pyruvate dehydrogenase protein X component, mitochondrial OS=Mus musculus GN=Pdhx PE=2 SV=1 | 15 | 0.940 | 0.770 | 0.805 |
| sp|P63330|PP2AA_MOUSE | Serine/threonine-protein phosphatase 2A catalytic subunit alpha isoform OS=Mus musculus GN=Ppp2ca PE=1 SV=1 | 15 | 0.886 | 0.890 | 0.807 |
| sp|P50429|ARSB_MOUSE | Arylsulfatase B OS=Mus musculus GN=Arsb PE=2 SV=3 | 15 | 0.823 | 0.788 | 0.812 |
| sp|P62715|PP2AB_MOUSE | Serine/threonine-protein phosphatase 2A catalytic subunit beta isoform OS=Mus musculus GN=Ppp2cb PE=1 SV=1 | 15 | 0.886 | 0.890 | 0.813 |
| sp|P67871|CSK2B_MOUSE | Casein kinase II subunit beta OS=Mus musculus GN=Csnk2b PE=1 SV=1 | 15 | 0.679 | 0.679 | 0.814 |
| sp|Q61206|PA1B2_MOUSE | Platelet-activating factor acetylhydrolase IB subunit beta OS=Mus musculus GN=Pafah1b2 PE=1 SV=2 | 15 | 1.000 | 1.040 | 0.819 |
| sp|Q8BWY3|ERF1_MOUSE | Eukaryotic peptide chain release factor subunit 1 OS=Mus musculus GN=Etf1 PE=1 SV=4 | 15 | 0.737 | 0.848 | 0.839 |
| sp|Q6ZWU9|RS27_MOUSE | 40S ribosomal protein S27 OS=Mus musculus GN=Rps27 PE=1 SV=3 | 15 | 1.148 | 0.931 | 0.846 |
| sp|Q9CQR4|ACO13_MOUSE | Acyl-coenzyme A thioesterase 13 OS=Mus musculus GN=Acot13 PE=1 SV=1 | 15 | 1.515 | 1.030 | 0.885 |
| sp|Q9CYZ2|TPD54_MOUSE | Tumor protein D54 OS=Mus musculus GN=Tpd52l2 PE=1 SV=1 | 15 | 1.277 | 0.978 | 0.887 |
| sp|O35226|PSMD4_MOUSE | 26S proteasome non-ATPase regulatory subunit 4 OS=Mus musculus GN=Psmd4 PE=1 SV=1 | 15 | 1.053 | 0.871 | 0.917 |
| sp|Q91VW3|SH3L3_MOUSE | SH3 domain-binding glutamic acid-rich-like protein 3 OS=Mus musculus GN=Sh3bgrl3 PE=1 SV=1 | 15 | 1.542 | 0.983 | 0.926 |
| sp|Q9CZU3|SK2L2_MOUSE | Superkiller viralicidic activity 2-like 2 OS=Mus musculus GN=Skiv2l2 PE=2 SV=1 | 15 | 0.000 | 0.906 | 0.975 |
| sp|Q922Q8|LRC59_MOUSE | Leucine-rich repeat-containing protein 59 OS=Mus musculus GN=Lrrc59 PE=2 SV=1 | 15 | 1.040 | 0.000 | 0.985 |
| sp|Q8JZN5|ACAD9_MOUSE | Acyl-CoA dehydrogenase family member 9, mitochondrial OS=Mus musculus GN=Acad9 PE=2 SV=2 | 15 | 0.000 | 1.795 | 0.995 |
| sp|Q9JKX6|NUDT5_MOUSE | ADP-sugar pyrophosphatase OS=Mus musculus GN=Nudt5 PE=1 SV=1 | 15 | 1.108 | 0.959 | 1.062 |
| sp|Q3UVG3|F91A1_MOUSE | Protein FAM91A1 OS=Mus musculus GN=Fam91a1 PE=1 SV=1 | 15 | 1.218 | 0.992 | 1.076 |
| sp|P13439|UMPS_MOUSE | Uridine 5&#039;-monophosphate synthase OS=Mus musculus GN=Umps PE=2 SV=3 | 15 | 1.122 | 1.090 | 1.078 |
| sp|P54276|MSH6_MOUSE | DNA mismatch repair protein Msh6 OS=Mus musculus GN=Msh6 PE=1 SV=3 | 15 | 0.756 | 1.373 | 1.106 |
| sp|Q8C0L6|PAOX_MOUSE | Peroxisomal N(1)-acetyl-spermine/spermidine oxidase OS=Mus musculus GN=Paox PE=1 SV=3 | 15 | InD | 1.045 | 1.178 |
| sp|Q62448|IF4G2_MOUSE | Eukaryotic translation initiation factor 4 gamma 2 OS=Mus musculus GN=Eif4g2 PE=1 SV=2 | 15 | 1.184 | 1.007 | 1.190 |
| sp|Q8R0G9|NU133_MOUSE | Nuclear pore complex protein Nup133 OS=Mus musculus GN=Nup133 PE=1 SV=2 | 15 | 1.191 | InD | 6.529 |
| sp|Q9QUI0|RHOA_MOUSE | Transforming protein RhoA OS=Mus musculus GN=Rhoa PE=1 SV=1 | 15 | 0.724 | 0.791 | InD |
| sp|Q9DCG9|TR112_MOUSE | tRNA methyltransferase 112 homolog OS=Mus musculus GN=Trmt112 PE=2 SV=1 | 15 | 1.195 | 1.009 | InD |
| sp|Q9CYN2|SPCS2_MOUSE | Signal peptidase complex subunit 2 OS=Mus musculus GN=Spcs2 PE=2 SV=1 | 15 | 1.361 | 1.071 | InD |
| sp|P61226|RAP2B_MOUSE | Ras-related protein Rap-2b OS=Mus musculus GN=Rap2b PE=1 SV=1 | 15 | 1.263 | 1.285 | InD |
| sp|P30115|GSTA3_MOUSE | Glutathione S-transferase A3 OS=Mus musculus GN=Gsta3 PE=1 SV=2 | 14 | 0.000 | InD | 0.385 |
| sp|Q9ESE1|LRBA_MOUSE | Lipopolysaccharide-responsive and beige-like anchor protein OS=Mus musculus GN=Lrba PE=1 SV=1 | 14 | 1.143 | 0.877 | 0.545 |
| sp|O55023|IMPA1_MOUSE | Inositol monophosphatase 1 OS=Mus musculus GN=Impa1 PE=1 SV=1 | 14 | 0.946 | 0.879 | 0.632 |
| sp|Q9D8W7|OCAD2_MOUSE | OCIA domain-containing protein 2 OS=Mus musculus GN=Ociad2 PE=2 SV=1 | 14 | InD | 0.775 | 0.639 |
| sp|Q3U186|SYRM_MOUSE | Probable arginine--tRNA ligase, mitochondrial OS=Mus musculus GN=Rars2 PE=2 SV=1 | 14 | 0.000 | 0.620 | 0.659 |
| sp|Q9Z0W3|NU160_MOUSE | Nuclear pore complex protein Nup160 OS=Mus musculus GN=Nup160 PE=1 SV=2 | 14 | 0.973 | 0.615 | 0.682 |
| sp|P21279|GNAQ_MOUSE | Guanine nucleotide-binding protein G(q) subunit alpha OS=Mus musculus GN=Gnaq PE=1 SV=4 | 14 | 1.066 | 1.164 | 0.696 |
| sp|Q01279|EGFR_MOUSE | Epidermal growth factor receptor OS=Mus musculus GN=Egfr PE=1 SV=1 | 14 | 1.354 | 0.593 | 0.700 |
| sp|Q9CQU0|TXD12_MOUSE | Thioredoxin domain-containing protein 12 OS=Mus musculus GN=Txndc12 PE=2 SV=1 | 14 | 1.125 | 0.961 | 0.711 |
| sp|Q8BMD8|SCMC1_MOUSE | Calcium-binding mitochondrial carrier protein SCaMC-1 OS=Mus musculus GN=Slc25a24 PE=2 SV=1 | 14 | 1.297 | 1.453 | 0.740 |
| sp|Q64435|UD16_MOUSE | UDP-glucuronosyltransferase 1-6 OS=Mus musculus GN=Ugt1a6 PE=1 SV=1 | 14 | 1.103 | 0.773 | 0.742 |
| sp|P70302|STIM1_MOUSE | Stromal interaction molecule 1 OS=Mus musculus GN=Stim1 PE=1 SV=2 | 14 | 1.095 | 4.235 | 0.748 |
| sp|Q60865|CAPR1_MOUSE | Caprin-1 OS=Mus musculus GN=Caprin1 PE=1 SV=2 | 14 | 0.000 | 0.986 | 0.762 |
| sp|P31938|MP2K1_MOUSE | Dual specificity mitogen-activated protein kinase kinase 1 OS=Mus musculus GN=Map2k1 PE=1 SV=2 | 14 | 0.995 | 0.885 | 0.779 |
| sp|Q9D0S9|HINT2_MOUSE | Histidine triad nucleotide-binding protein 2, mitochondrial OS=Mus musculus GN=Hint2 PE=2 SV=1 | 14 | 0.977 | 0.899 | 0.788 |
| sp|Q9WTX6|CUL1_MOUSE | Cullin-1 OS=Mus musculus GN=Cul1 PE=1 SV=1 | 14 | 1.164 | InD | 0.798 |
| sp|P63321|RALA_MOUSE | Ras-related protein Ral-A OS=Mus musculus GN=Rala PE=1 SV=1 | 14 | 0.838 | 0.814 | 0.808 |
| sp|Q9DAK9|PHP14_MOUSE | 14 kDa phosphohistidine phosphatase OS=Mus musculus GN=Phpt1 PE=1 SV=1 | 14 | 1.000 | 0.545 | 0.822 |
| sp|P54103|DNJC2_MOUSE | DnaJ homolog subfamily C member 2 OS=Mus musculus GN=Dnajc2 PE=1 SV=2 | 14 | 1.533 | 0.000 | 0.839 |
| sp|Q9D1R9|RL34_MOUSE | 60S ribosomal protein L34 OS=Mus musculus GN=Rpl34 PE=3 SV=2 | 14 | 1.288 | 0.716 | 0.841 |
| sp|P52825|CPT2_MOUSE | Carnitine O-palmitoyltransferase 2, mitochondrial OS=Mus musculus GN=Cpt2 PE=1 SV=2 | 14 | 1.520 | 1.318 | 0.851 |
| sp|Q91YP2|NEUL_MOUSE | Neurolysin, mitochondrial OS=Mus musculus GN=Nln PE=2 SV=1 | 14 | 1.033 | 2.124 | 0.857 |
| sp|P42125|ECI1_MOUSE | Enoyl-CoA delta isomerase 1, mitochondrial OS=Mus musculus GN=Eci1 PE=2 SV=2 | 14 | 0.000 | InD | 0.858 |
| sp|Q8R317|UBQL1_MOUSE | Ubiquilin-1 OS=Mus musculus GN=Ubqln1 PE=1 SV=1 | 14 | 0.550 | 1.070 | 0.885 |
| sp|Q9DCS9|NDUBA_MOUSE | NADH dehydrogenase [ubiquinone] 1 beta subcomplex subunit 10 OS=Mus musculus GN=Ndufb10 PE=1 SV=3 | 14 | 0.960 | 0.632 | 0.900 |
| sp|Q99LS3|SERB_MOUSE | Phosphoserine phosphatase OS=Mus musculus GN=Psph PE=1 SV=1 | 14 | 1.182 | 1.027 | 0.946 |
| sp|P47753|CAZA1_MOUSE | F-actin-capping protein subunit alpha-1 OS=Mus musculus GN=Capza1 PE=1 SV=4 | 14 | InD | 1.069 | 0.952 |
| sp|Q9WV32|ARC1B_MOUSE | Actin-related protein 2/3 complex subunit 1B OS=Mus musculus GN=Arpc1b PE=1 SV=4 | 14 | 1.486 | 0.839 | 0.983 |
| sp|Q9Z1G3|VATC1_MOUSE | V-type proton ATPase subunit C 1 OS=Mus musculus GN=Atp6v1c1 PE=1 SV=4 | 14 | 1.045 | 0.000 | 0.986 |
| sp|Q8BHC4|DCAKD_MOUSE | Dephospho-CoA kinase domain-containing protein OS=Mus musculus GN=Dcakd PE=2 SV=1 | 14 | 1.429 | 0.763 | 0.990 |
| sp|Q9CX56|PSMD8_MOUSE | 26S proteasome non-ATPase regulatory subunit 8 OS=Mus musculus GN=Psmd8 PE=1 SV=2 | 14 | 1.277 | 0.908 | 1.000 |
| sp|P97315|CSRP1_MOUSE | Cysteine and glycine-rich protein 1 OS=Mus musculus GN=Csrp1 PE=1 SV=3 | 14 | 1.087 | 0.889 | 1.009 |
| sp|Q9CZW5|TOM70_MOUSE | Mitochondrial import receptor subunit TOM70 OS=Mus musculus GN=Tomm70a PE=1 SV=2 | 14 | InD | 1.061 | 1.023 |
| sp|Q7TNC4|LC7L2_MOUSE | Putative RNA-binding protein Luc7-like 2 OS=Mus musculus GN=Luc7l2 PE=1 SV=1 | 14 | 0.885 | 1.090 | 1.055 |
| sp|Q03173|ENAH_MOUSE | Protein enabled homolog OS=Mus musculus GN=Enah PE=1 SV=2 | 14 | 0.860 | 0.936 | 1.056 |
| sp|P63280|UBC9_MOUSE | SUMO-conjugating enzyme UBC9 OS=Mus musculus GN=Ube2i PE=1 SV=1 | 14 | 1.020 | 0.935 | 1.076 |
| sp|Q9CQI7|RU2B_MOUSE | U2 small nuclear ribonucleoprotein B&#039;&#039; OS=Mus musculus GN=Snrpb2 PE=2 SV=1 | 14 | InD | 1.165 | 1.080 |
| sp|Q6P8I4|PCNP_MOUSE | PEST proteolytic signal-containing nuclear protein OS=Mus musculus GN=Pcnp PE=1 SV=1 | 14 | 1.053 | 0.000 | 1.083 |
| sp|P10833|RRAS_MOUSE | Ras-related protein R-Ras OS=Mus musculus GN=Rras PE=2 SV=1 | 14 | 1.429 | 1.413 | 1.120 |
| sp|Q921F4|HNRLL_MOUSE | Heterogeneous nuclear ribonucleoprotein L-like OS=Mus musculus GN=Hnrpll PE=1 SV=3 | 14 | 1.074 | InD | 1.193 |
| sp|Q9WVA2|TIM8A_MOUSE | Mitochondrial import inner membrane translocase subunit Tim8 A OS=Mus musculus GN=Timm8a1 PE=1 SV=1 | 14 | 0.443 | 0.761 | 1.222 |
| sp|Q61081|CDC37_MOUSE | Hsp90 co-chaperone Cdc37 OS=Mus musculus GN=Cdc37 PE=2 SV=1 | 14 | 0.941 | 0.755 | 1.267 |
| sp|Q64674|SPEE_MOUSE | Spermidine synthase OS=Mus musculus GN=Srm PE=2 SV=1 | 14 | 1.043 | 0.965 | 1.358 |
| sp|O70310|NMT1_MOUSE | Glycylpeptide N-tetradecanoyltransferase 1 OS=Mus musculus GN=Nmt1 PE=1 SV=1 | 14 | 1.406 | 1.096 | 1.382 |
| sp|P62492|RB11A_MOUSE | Ras-related protein Rab-11A OS=Mus musculus GN=Rab11a PE=1 SV=3 | 14 | InD | 0.000 | InD |
| sp|Q61087|LAMB3_MOUSE | Laminin subunit beta-3 OS=Mus musculus GN=Lamb3 PE=2 SV=2 | 14 | 1.150 | 1.000 | InD |
| sp|Q9Z172|SUMO3_MOUSE | Small ubiquitin-related modifier 3 OS=Mus musculus GN=Sumo3 PE=2 SV=1 | 14 | 0.939 | InD | InD |
| sp|Q9D358|PPAC_MOUSE | Low molecular weight phosphotyrosine protein phosphatase OS=Mus musculus GN=Acp1 PE=1 SV=3 | 13 | 1.029 | 0.933 | 0.000 |
| sp|P46467|VPS4B_MOUSE | Vacuolar protein sorting-associated protein 4B OS=Mus musculus GN=Vps4b PE=1 SV=2 | 13 | 0.961 | 0.936 | 0.000 |
| sp|P16460|ASSY_MOUSE | Argininosuccinate synthase OS=Mus musculus GN=Ass1 PE=1 SV=1 | 13 | 0.556 | 0.800 | 0.452 |
| sp|Q9EPB4|ASC_MOUSE | Apoptosis-associated speck-like protein containing a CARD OS=Mus musculus GN=Pycard PE=1 SV=1 | 13 | 0.826 | 0.939 | 0.575 |
| sp|O88986|KBL_MOUSE | 2-amino-3-ketobutyrate coenzyme A ligase, mitochondrial OS=Mus musculus GN=Gcat PE=1 SV=2 | 13 | 1.136 | 0.971 | 0.609 |
| sp|O35566|CD151_MOUSE | CD151 antigen OS=Mus musculus GN=Cd151 PE=2 SV=2 | 13 | 0.826 | InD | 0.629 |
| sp|P27046|MA2A1_MOUSE | Alpha-mannosidase 2 OS=Mus musculus GN=Man2a1 PE=1 SV=2 | 13 | 1.117 | InD | 0.716 |
| sp|Q91XU3|PI42C_MOUSE | Phosphatidylinositol 5-phosphate 4-kinase type-2 gamma OS=Mus musculus GN=Pip4k2c PE=2 SV=1 | 13 | InD | 0.921 | 0.746 |
| sp|P56135|ATPK_MOUSE | ATP synthase subunit f, mitochondrial OS=Mus musculus GN=Atp5j2 PE=1 SV=3 | 13 | 1.039 | 0.875 | 0.767 |
| sp|Q6ZWY3|RS27L_MOUSE | 40S ribosomal protein S27-like OS=Mus musculus GN=Rps27l PE=2 SV=3 | 13 | 1.154 | 1.359 | 0.787 |
| sp|Q9D711|PIR_MOUSE | Pirin OS=Mus musculus GN=Pir PE=2 SV=1 | 13 | 1.985 | 1.000 | 0.790 |
| sp|Q6GQT9|NOMO1_MOUSE | Nodal modulator 1 OS=Mus musculus GN=Nomo1 PE=1 SV=1 | 13 | 0.958 | 0.000 | 0.796 |
| sp|P11031|TCP4_MOUSE | Activated RNA polymerase II transcriptional coactivator p15 OS=Mus musculus GN=Sub1 PE=1 SV=3 | 13 | 1.024 | 0.978 | 0.799 |
| sp|Q99JX4|EIF3M_MOUSE | Eukaryotic translation initiation factor 3 subunit M OS=Mus musculus GN=Eif3m PE=2 SV=1 | 13 | 1.281 | 1.138 | 0.805 |
| sp|Q60631|GRB2_MOUSE | Growth factor receptor-bound protein 2 OS=Mus musculus GN=Grb2 PE=1 SV=1 | 13 | 1.021 | 0.830 | 0.860 |
| sp|P62889|RL30_MOUSE | 60S ribosomal protein L30 OS=Mus musculus GN=Rpl30 PE=2 SV=2 | 13 | 1.044 | 1.061 | 0.865 |
| sp|Q9CQC9|SAR1B_MOUSE | GTP-binding protein SAR1b OS=Mus musculus GN=Sar1b PE=1 SV=1 | 13 | 1.302 | 0.965 | 0.868 |
| sp|Q7TNV0|DEK_MOUSE | Protein DEK OS=Mus musculus GN=Dek PE=1 SV=1 | 13 | 0.971 | 1.023 | 0.873 |
| sp|Q80Y81|RNZ2_MOUSE | Zinc phosphodiesterase ELAC protein 2 OS=Mus musculus GN=Elac2 PE=1 SV=1 | 13 | 1.026 | InD | 0.873 |
| sp|Q6P8X1|SNX6_MOUSE | Sorting nexin-6 OS=Mus musculus GN=Snx6 PE=1 SV=2 | 13 | 1.012 | 0.896 | 0.875 |
| sp|Q9D883|U2AF1_MOUSE | Splicing factor U2AF 35 kDa subunit OS=Mus musculus GN=U2af1 PE=1 SV=4 | 13 | 1.029 | 1.017 | 0.878 |
| sp|Q8R010|AIMP2_MOUSE | Aminoacyl tRNA synthase complex-interacting multifunctional protein 2 OS=Mus musculus GN=Aimp2 PE=1 SV=2 | 13 | 1.310 | 0.944 | 0.891 |
| sp|Q64520|KGUA_MOUSE | Guanylate kinase OS=Mus musculus GN=Guk1 PE=1 SV=2 | 13 | 0.787 | 1.199 | 0.917 |
| sp|P00405|COX2_MOUSE | Cytochrome c oxidase subunit 2 OS=Mus musculus GN=Mtco2 PE=1 SV=1 | 13 | 1.129 | 0.912 | 0.921 |
| sp|Q9JKB1|UCHL3_MOUSE | Ubiquitin carboxyl-terminal hydrolase isozyme L3 OS=Mus musculus GN=Uchl3 PE=1 SV=2 | 13 | 1.173 | 0.894 | 0.958 |
| sp|Q9D898|ARP5L_MOUSE | Actin-related protein 2/3 complex subunit 5-like protein OS=Mus musculus GN=Arpc5l PE=1 SV=1 | 13 | 0.895 | 0.847 | 0.964 |
| sp|Q3UHX2|HAP28_MOUSE | 28 kDa heat- and acid-stable phosphoprotein OS=Mus musculus GN=Pdap1 PE=1 SV=1 | 13 | 1.045 | 0.000 | 0.965 |
| sp|P97461|RS5_MOUSE | 40S ribosomal protein S5 OS=Mus musculus GN=Rps5 PE=2 SV=3 | 13 | InD | 0.976 | 0.988 |
| sp|Q6ZPJ3|UBE2O_MOUSE | Ubiquitin-conjugating enzyme E2 O OS=Mus musculus GN=Ube2o PE=1 SV=3 | 13 | 1.101 | 1.020 | 1.000 |
| sp|Q99JB2|STML2_MOUSE | Stomatin-like protein 2 OS=Mus musculus GN=Stoml2 PE=1 SV=1 | 13 | 1.112 | 1.061 | 1.000 |
| sp|Q8BHG1|NRDC_MOUSE | Nardilysin OS=Mus musculus GN=Nrd1 PE=1 SV=1 | 13 | 0.000 | 0.773 | 1.057 |
| sp|P70404|IDHG1_MOUSE | Isocitrate dehydrogenase [NAD] subunit gamma 1, mitochondrial OS=Mus musculus GN=Idh3g PE=1 SV=1 | 13 | 1.202 | InD | 1.069 |
| sp|Q9WUK2|IF4H_MOUSE | Eukaryotic translation initiation factor 4H OS=Mus musculus GN=Eif4h PE=1 SV=3 | 13 | 1.647 | 1.041 | 1.081 |
| sp|Q9CW46|RAVR1_MOUSE | Ribonucleoprotein PTB-binding 1 OS=Mus musculus GN=Raver1 PE=1 SV=2 | 13 | 1.348 | 1.392 | 1.084 |
| sp|Q8BMP6|GCP60_MOUSE | Golgi resident protein GCP60 OS=Mus musculus GN=Acbd3 PE=1 SV=3 | 13 | 0.779 | 0.927 | 1.107 |
| sp|Q9D3P8|PLRKT_MOUSE | Plasminogen receptor (KT) OS=Mus musculus GN=Plgrkt PE=1 SV=1 | 13 | 1.233 | InD | 1.125 |
| sp|Q8K224|NAT10_MOUSE | N-acetyltransferase 10 OS=Mus musculus GN=Nat10 PE=2 SV=1 | 13 | 2.098 | InD | 1.141 |
| sp|Q9ET54|PALLD_MOUSE | Palladin OS=Mus musculus GN=Palld PE=1 SV=2 | 13 | 1.500 | 0.571 | 2.405 |
| sp|P62137|PP1A_MOUSE | Serine/threonine-protein phosphatase PP1-alpha catalytic subunit OS=Mus musculus GN=Ppp1ca PE=1 SV=1 | 13 | InD | 0.792 | InD |
| sp|P47809|MP2K4_MOUSE | Dual specificity mitogen-activated protein kinase kinase 4 OS=Mus musculus GN=Map2k4 PE=1 SV=2 | 13 | 1.012 | 1.006 | InD |
| sp|Q9QYJ3|DNJB1_MOUSE | DnaJ homolog subfamily B member 1 OS=Mus musculus GN=Dnajb1 PE=2 SV=3 | 13 | 1.342 | 1.286 | InD |
| sp|P09041|PGK2_MOUSE | Phosphoglycerate kinase 2 OS=Mus musculus GN=Pgk2 PE=1 SV=4 | 13 | InD | InD | InD |
| sp|P59326|YTHD1_MOUSE | YTH domain family protein 1 OS=Mus musculus GN=Ythdf1 PE=2 SV=1 | 12 | 1.230 | 0.214 | 0.000 |
| sp|Q921G7|ETFD_MOUSE | Electron transfer flavoprotein-ubiquinone oxidoreductase, mitochondrial OS=Mus musculus GN=Etfdh PE=1 SV=1 | 12 | InD | 0.829 | 0.000 |
| sp|P04104|K2C1_MOUSE | Keratin, type II cytoskeletal 1 OS=Mus musculus GN=Krt1 PE=1 SV=4 | 12 | 0.000 | 1.020 | 0.000 |
| sp|Q6ZWM4|NAA38_MOUSE | N-alpha-acetyltransferase 38, NatC auxiliary subunit OS=Mus musculus GN=Naa38 PE=2 SV=3 | 12 | 0.758 | 1.023 | 0.000 |
| sp|Q8BH86|CN159_MOUSE | UPF0317 protein C14orf159 homolog, mitochondrial OS=Mus musculus PE=2 SV=1 | 12 | InD | 1.349 | 0.000 |
| sp|P46935|NEDD4_MOUSE | E3 ubiquitin-protein ligase NEDD4 OS=Mus musculus GN=Nedd4 PE=1 SV=3 | 12 | 0.974 | InD | 0.000 |
| sp|Q8CGK3|LONM_MOUSE | Lon protease homolog, mitochondrial OS=Mus musculus GN=Lonp1 PE=1 SV=2 | 12 | 1.022 | InD | 0.000 |
| sp|Q9WV80|SNX1_MOUSE | Sorting nexin-1 OS=Mus musculus GN=Snx1 PE=1 SV=1 | 12 | 1.367 | InD | 0.000 |
| sp|Q149F3|ERF3B_MOUSE | Eukaryotic peptide chain release factor GTP-binding subunit ERF3B OS=Mus musculus GN=Gspt2 PE=1 SV=1 | 12 | 1.450 | InD | 0.288 |
| sp|Q8R050|ERF3A_MOUSE | Eukaryotic peptide chain release factor GTP-binding subunit ERF3A OS=Mus musculus GN=Gspt1 PE=1 SV=2 | 12 | 1.547 | InD | 0.407 |
| sp|Q61823|PDCD4_MOUSE | Programmed cell death protein 4 OS=Mus musculus GN=Pdcd4 PE=1 SV=1 | 12 | 0.579 | 0.509 | 0.523 |
| sp|P56380|AP4A_MOUSE | Bis(5&#039;-nucleosyl)-tetraphosphatase [asymmetrical] OS=Mus musculus GN=Nudt2 PE=1 SV=3 | 12 | 0.572 | 1.076 | 0.578 |
| sp|Q921L3|TMCO1_MOUSE | Transmembrane and coiled-coil domain-containing protein 1 OS=Mus musculus GN=Tmco1 PE=2 SV=1 | 12 | 0.938 | 0.813 | 0.585 |
| sp|Q8BGV3|TACD2_MOUSE | Tumor-associated calcium signal transducer 2 OS=Mus musculus GN=Tacstd2 PE=2 SV=1 | 12 | 0.000 | 0.741 | 0.593 |
| sp|P55012|S12A2_MOUSE | Solute carrier family 12 member 2 OS=Mus musculus GN=Slc12a2 PE=1 SV=2 | 12 | 0.270 | 1.250 | 0.636 |
| sp|Q9D0F3|LMAN1_MOUSE | Protein ERGIC-53 OS=Mus musculus GN=Lman1 PE=2 SV=1 | 12 | 1.119 | InD | 0.654 |
| sp|Q8K3W0|BRE_MOUSE | BRCA1-A complex subunit BRE OS=Mus musculus GN=Bre PE=1 SV=2 | 12 | 1.047 | 1.012 | 0.659 |
| sp|Q64442|DHSO_MOUSE | Sorbitol dehydrogenase OS=Mus musculus GN=Sord PE=1 SV=3 | 12 | 1.014 | InD | 0.667 |
| sp|Q5SVR0|TBC9B_MOUSE | TBC1 domain family member 9B OS=Mus musculus GN=Tbc1d9b PE=2 SV=1 | 12 | 1.008 | 0.686 | 0.772 |
| sp|O35841|API5_MOUSE | Apoptosis inhibitor 5 OS=Mus musculus GN=Api5 PE=2 SV=2 | 12 | 1.103 | 0.000 | 0.782 |
| sp|Q63829|COMD3_MOUSE | COMM domain-containing protein 3 OS=Mus musculus GN=Commd3 PE=2 SV=1 | 12 | 1.017 | 0.849 | 0.789 |
| sp|Q811D0|DLG1_MOUSE | Disks large homolog 1 OS=Mus musculus GN=Dlg1 PE=1 SV=1 | 12 | 0.000 | InD | 0.791 |
| sp|P50136|ODBA_MOUSE | 2-oxoisovalerate dehydrogenase subunit alpha, mitochondrial OS=Mus musculus GN=Bckdha PE=1 SV=1 | 12 | 2.882 | 0.793 | 0.800 |
| sp|P83741|WNK1_MOUSE | Serine/threonine-protein kinase WNK1 OS=Mus musculus GN=Wnk1 PE=1 SV=2 | 12 | 1.028 | 1.077 | 0.803 |
| sp|Q9R1J0|NSDHL_MOUSE | Sterol-4-alpha-carboxylate 3-dehydrogenase, decarboxylating OS=Mus musculus GN=Nsdhl PE=2 SV=1 | 12 | 1.304 | 0.000 | 0.810 |
| sp|Q62376|RU17_MOUSE | U1 small nuclear ribonucleoprotein 70 kDa OS=Mus musculus GN=Snrnp70 PE=1 SV=2 | 12 | 0.000 | 0.000 | 0.822 |
| sp|Q9CXF4|TBC15_MOUSE | TBC1 domain family member 15 OS=Mus musculus GN=Tbc1d15 PE=1 SV=1 | 12 | 1.058 | 1.020 | 0.825 |
| sp|P62869|ELOB_MOUSE | Transcription elongation factor B polypeptide 2 OS=Mus musculus GN=Tceb2 PE=1 SV=1 | 12 | 0.918 | 0.814 | 0.843 |
| sp|Q9DBL1|ACDSB_MOUSE | Short/branched chain specific acyl-CoA dehydrogenase, mitochondrial OS=Mus musculus GN=Acadsb PE=1 SV=1 | 12 | 0.976 | 0.840 | 0.856 |
| sp|P20065|TYB4_MOUSE | Thymosin beta-4 OS=Mus musculus GN=Tmsb4x PE=1 SV=1 | 12 | InD | 1.410 | 0.867 |
| sp|Q99K48|NONO_MOUSE | Non-POU domain-containing octamer-binding protein OS=Mus musculus GN=Nono PE=1 SV=3 | 12 | 1.135 | 0.919 | 0.869 |
| sp|Q8VE88|F1142_MOUSE | Protein FAM114A2 OS=Mus musculus GN=Fam114a2 PE=1 SV=2 | 12 | 1.110 | 0.766 | 0.870 |
| sp|Q9ERS2|NDUAD_MOUSE | NADH dehydrogenase [ubiquinone] 1 alpha subcomplex subunit 13 OS=Mus musculus GN=Ndufa13 PE=1 SV=3 | 12 | 1.000 | 0.918 | 0.890 |
| sp|P02301|H3C_MOUSE | Histone H3.3C OS=Mus musculus GN=H3f3c PE=1 SV=3 | 12 | 0.907 | 1.231 | 0.897 |
| sp|P84228|H32_MOUSE | Histone H3.2 OS=Mus musculus GN=Hist1h3b PE=1 SV=2 | 12 | 0.907 | 1.231 | 0.897 |
| sp|P84244|H33_MOUSE | Histone H3.3 OS=Mus musculus GN=H3f3a PE=1 SV=2 | 12 | 0.907 | 1.231 | 0.897 |
| sp|P68433|H31_MOUSE | Histone H3.1 OS=Mus musculus GN=Hist1h3a PE=1 SV=2 | 12 | 0.909 | 1.244 | 0.897 |
| sp|Q8JZS0|LIN7A_MOUSE | Protein lin-7 homolog A OS=Mus musculus GN=Lin7a PE=1 SV=2 | 12 | 0.947 | 0.939 | 0.903 |
| sp|O88952|LIN7C_MOUSE | Protein lin-7 homolog C OS=Mus musculus GN=Lin7c PE=1 SV=2 | 12 | 0.947 | 0.939 | 0.909 |
| sp|P62830|RL23_MOUSE | 60S ribosomal protein L23 OS=Mus musculus GN=Rpl23 PE=1 SV=1 | 12 | 0.958 | 0.884 | 0.920 |
| sp|P47915|RL29_MOUSE | 60S ribosomal protein L29 OS=Mus musculus GN=Rpl29 PE=2 SV=2 | 12 | 1.057 | 0.989 | 0.927 |
| sp|Q60692|PSB6_MOUSE | Proteasome subunit beta type-6 OS=Mus musculus GN=Psmb6 PE=1 SV=3 | 12 | 1.025 | 1.059 | 0.944 |
| sp|O88738|BIRC6_MOUSE | Baculoviral IAP repeat-containing protein 6 OS=Mus musculus GN=Birc6 PE=1 SV=2 | 12 | 1.655 | 0.846 | 0.949 |
| sp|P49442|INPP_MOUSE | Inositol polyphosphate 1-phosphatase OS=Mus musculus GN=Inpp1 PE=1 SV=2 | 12 | 1.091 | 1.291 | 0.959 |
| sp|P97450|ATP5J_MOUSE | ATP synthase-coupling factor 6, mitochondrial OS=Mus musculus GN=Atp5j PE=1 SV=1 | 12 | 1.100 | 0.000 | 1.000 |
| sp|Q9JM62|REEP6_MOUSE | Receptor expression-enhancing protein 6 OS=Mus musculus GN=Reep6 PE=2 SV=1 | 12 | 1.063 | 0.959 | 1.023 |
| sp|P62849|RS24_MOUSE | 40S ribosomal protein S24 OS=Mus musculus GN=Rps24 PE=1 SV=1 | 12 | 1.139 | 1.000 | 1.024 |
| sp|Q9JL35|HMGN5_MOUSE | High mobility group nucleosome-binding domain-containing protein 5 OS=Mus musculus GN=Hmgn5 PE=1 SV=2 | 12 | 1.019 | 0.872 | 1.083 |
| sp|Q61160|FADD_MOUSE | Protein FADD OS=Mus musculus GN=Fadd PE=1 SV=1 | 12 | 1.318 | 1.007 | 1.138 |
| sp|O70435|PSA3_MOUSE | Proteasome subunit alpha type-3 OS=Mus musculus GN=Psma3 PE=1 SV=3 | 12 | 1.149 | 1.032 | 1.245 |
| sp|Q9CQF9|PCYOX_MOUSE | Prenylcysteine oxidase OS=Mus musculus GN=Pcyox1 PE=1 SV=1 | 12 | 1.096 | 0.853 | 1.707 |
| sp|Q80VJ3|RCL_MOUSE | Deoxyribonucleoside 5&#039;-monophosphate N-glycosidase OS=Mus musculus GN=Rcl PE=2 SV=2 | 12 | 1.018 | 0.770 | InD |
| sp|P47857|K6PF_MOUSE | 6-phosphofructokinase, muscle type OS=Mus musculus GN=Pfkm PE=1 SV=3 | 12 | 1.092 | 0.878 | InD |
| sp|Q9CQ92|FIS1_MOUSE | Mitochondrial fission 1 protein OS=Mus musculus GN=Fis1 PE=1 SV=1 | 12 | 0.879 | 0.954 | InD |
| sp|Q9DCF9|SSRG_MOUSE | Translocon-associated protein subunit gamma OS=Mus musculus GN=Ssr3 PE=1 SV=1 | 12 | 1.122 | 0.978 | InD |
| sp|Q9D1K2|VATF_MOUSE | V-type proton ATPase subunit F OS=Mus musculus GN=Atp6v1f PE=1 SV=2 | 12 | 1.316 | 1.154 | InD |
| sp|Q923G2|RPAB3_MOUSE | DNA-directed RNA polymerases I, II, and III subunit RPABC3 OS=Mus musculus GN=Polr2h PE=2 SV=3 | 12 | 1.308 | 1.155 | InD |
| sp|Q9D892|ITPA_MOUSE | Inosine triphosphate pyrophosphatase OS=Mus musculus GN=Itpa PE=1 SV=2 | 12 | 1.299 | 1.160 | InD |
| sp|Q9D0M3|CY1_MOUSE | Cytochrome c1, heme protein, mitochondrial OS=Mus musculus GN=Cyc1 PE=1 SV=1 | 12 | 1.046 | 1.167 | InD |
| sp|Q9WV02|RBMX_MOUSE | RNA-binding motif protein, X chromosome OS=Mus musculus GN=Rbmx PE=1 SV=1 | 11 | 0.000 | 0.000 | 0.000 |
| sp|Q9Z2W1|STK25_MOUSE | Serine/threonine-protein kinase 25 OS=Mus musculus GN=Stk25 PE=1 SV=2 | 11 | 1.129 | 0.686 | 0.000 |
| sp|Q9DBR1|XRN2_MOUSE | 5&#039;-3&#039; exoribonuclease 2 OS=Mus musculus GN=Xrn2 PE=1 SV=1 | 11 | 1.686 | 0.895 | 0.000 |
| sp|Q9ESX5|DKC1_MOUSE | H/ACA ribonucleoprotein complex subunit 4 OS=Mus musculus GN=Dkc1 PE=1 SV=4 | 11 | 1.359 | 0.930 | 0.000 |
| sp|Q9D8T7|SLIRP_MOUSE | SRA stem-loop-interacting RNA-binding protein, mitochondrial OS=Mus musculus GN=Slirp PE=1 SV=2 | 11 | 1.074 | 1.202 | 0.000 |
| sp|Q8CIH5|PLCG2_MOUSE | 1-phosphatidylinositol 4,5-bisphosphate phosphodiesterase gamma-2 OS=Mus musculus GN=Plcg2 PE=1 SV=1 | 11 | 1.092 | InD | 0.000 |
| sp|P53395|ODB2_MOUSE | Lipoamide acyltransferase component of branched-chain alpha-keto acid dehydrogenase complex, mitochondrial OS=Mus musculus GN=Dbt PE=2 SV=2 | 11 | 1.351 | InD | 0.523 |
| sp|P62862|RS30_MOUSE | 40S ribosomal protein S30 OS=Mus musculus GN=Fau PE=3 SV=1 | 11 | InD | 0.852 | 0.530 |
| sp|Q3UPH1|PRRC1_MOUSE | Protein PRRC1 OS=Mus musculus GN=Prrc1 PE=2 SV=1 | 11 | 0.957 | 0.903 | 0.614 |
| sp|Q80ZS3|RT26_MOUSE | 28S ribosomal protein S26, mitochondrial OS=Mus musculus GN=Mrps26 PE=2 SV=1 | 11 | 0.914 | 0.884 | 0.705 |
| sp|Q8BJU0|SGTA_MOUSE | Small glutamine-rich tetratricopeptide repeat-containing protein alpha OS=Mus musculus GN=Sgta PE=1 SV=2 | 11 | 1.071 | 0.802 | 0.707 |
| sp|Q6PEB6|PHOCN_MOUSE | MOB-like protein phocein OS=Mus musculus GN=Mob4 PE=1 SV=1 | 11 | 1.354 | 0.920 | 0.729 |
| sp|Q8VCA8|SCRN2_MOUSE | Secernin-2 OS=Mus musculus GN=Scrn2 PE=2 SV=1 | 11 | 1.011 | 0.944 | 0.741 |
| sp|Q9JM83|CALM4_MOUSE | Calmodulin-4 OS=Mus musculus GN=Calm4 PE=2 SV=2 | 11 | 0.000 | InD | 0.750 |
| sp|O89017|LGMN_MOUSE | Legumain OS=Mus musculus GN=Lgmn PE=1 SV=1 | 11 | 1.242 | 0.000 | 0.763 |
| sp|Q921Q7|RIN1_MOUSE | Ras and Rab interactor 1 OS=Mus musculus GN=Rin1 PE=1 SV=1 | 11 | 0.000 | 0.700 | 0.804 |
| sp|Q6DID3|SCAF8_MOUSE | Protein SCAF8 OS=Mus musculus GN=Scaf8 PE=1 SV=1 | 11 | 0.902 | 1.117 | 0.818 |
| sp|Q9Z2Q5|RM40_MOUSE | 39S ribosomal protein L40, mitochondrial OS=Mus musculus GN=Mrpl40 PE=2 SV=2 | 11 | 1.383 | 1.139 | 0.821 |
| sp|P83917|CBX1_MOUSE | Chromobox protein homolog 1 OS=Mus musculus GN=Cbx1 PE=1 SV=1 | 11 | 0.884 | 1.450 | 0.843 |
| sp|P62274|RS29_MOUSE | 40S ribosomal protein S29 OS=Mus musculus GN=Rps29 PE=2 SV=2 | 11 | 1.114 | 0.969 | 0.847 |
| sp|Q3UEB3|PUF60_MOUSE | Poly(U)-binding-splicing factor PUF60 OS=Mus musculus GN=Puf60 PE=2 SV=2 | 11 | InD | InD | 0.869 |
| sp|Q6ZWX6|IF2A_MOUSE | Eukaryotic translation initiation factor 2 subunit 1 OS=Mus musculus GN=Eif2s1 PE=1 SV=3 | 11 | 1.179 | 0.000 | 0.876 |
| sp|P61211|ARL1_MOUSE | ADP-ribosylation factor-like protein 1 OS=Mus musculus GN=Arl1 PE=2 SV=1 | 11 | 1.154 | 0.854 | 0.889 |
| sp|Q9D2M8|UB2V2_MOUSE | Ubiquitin-conjugating enzyme E2 variant 2 OS=Mus musculus GN=Ube2v2 PE=2 SV=4 | 11 | 0.000 | InD | 0.915 |
| sp|Q9CQ69|QCR8_MOUSE | Cytochrome b-c1 complex subunit 8 OS=Mus musculus GN=Uqcrq PE=1 SV=3 | 11 | 1.126 | 0.963 | 0.920 |
| sp|Q8CAY6|THIC_MOUSE | Acetyl-CoA acetyltransferase, cytosolic OS=Mus musculus GN=Acat2 PE=1 SV=2 | 11 | 1.009 | 0.966 | 0.935 |
| sp|P19253|RL13A_MOUSE | 60S ribosomal protein L13a OS=Mus musculus GN=Rpl13a PE=1 SV=4 | 11 | 1.035 | 0.926 | 0.953 |
| sp|Q6PD26|PIGS_MOUSE | GPI transamidase component PIG-S OS=Mus musculus GN=Pigs PE=1 SV=3 | 11 | 1.242 | 1.031 | 0.962 |
| sp|P32233|DRG1_MOUSE | Developmentally-regulated GTP-binding protein 1 OS=Mus musculus GN=Drg1 PE=1 SV=1 | 11 | 1.143 | 1.012 | 0.974 |
| sp|Q8CHT0|AL4A1_MOUSE | Delta-1-pyrroline-5-carboxylate dehydrogenase, mitochondrial OS=Mus musculus GN=Aldh4a1 PE=1 SV=3 | 11 | 0.841 | 0.722 | 0.995 |
| sp|Q78IK4|APOOL_MOUSE | Apolipoprotein O-like OS=Mus musculus GN=Apool PE=2 SV=1 | 11 | 1.219 | 0.807 | 1.005 |
| sp|Q8CH18|CCAR1_MOUSE | Cell division cycle and apoptosis regulator protein 1 OS=Mus musculus GN=Ccar1 PE=1 SV=1 | 11 | 1.455 | 0.588 | 1.014 |
| sp|Q80TV8|CLAP1_MOUSE | CLIP-associating protein 1 OS=Mus musculus GN=Clasp1 PE=1 SV=2 | 11 | 1.390 | InD | 1.027 |
| sp|Q8BX17|GEMI5_MOUSE | Gem-associated protein 5 OS=Mus musculus GN=Gemin5 PE=1 SV=2 | 11 | 1.269 | 1.326 | 1.036 |
| sp|Q9D554|SF3A3_MOUSE | Splicing factor 3A subunit 3 OS=Mus musculus GN=Sf3a3 PE=2 SV=2 | 11 | 0.000 | 0.781 | 1.052 |
| sp|Q62077|PLCG1_MOUSE | 1-phosphatidylinositol 4,5-bisphosphate phosphodiesterase gamma-1 OS=Mus musculus GN=Plcg1 PE=1 SV=2 | 11 | 1.032 | 1.192 | 1.169 |
| sp|Q9CQT1|MTNA_MOUSE | Methylthioribose-1-phosphate isomerase OS=Mus musculus GN=Mri1 PE=2 SV=1 | 11 | 0.574 | 1.231 | 1.212 |
| sp|O70378|EMC8_MOUSE | ER membrane protein complex subunit 8 OS=Mus musculus GN=Emc8 PE=1 SV=1 | 11 | 0.901 | 0.984 | 1.351 |
| sp|Q64669|NQO1_MOUSE | NAD(P)H dehydrogenase [quinone] 1 OS=Mus musculus GN=Nqo1 PE=1 SV=3 | 11 | 1.276 | 1.447 | 1.581 |
| sp|Q9R1C7|PR40A_MOUSE | Pre-mRNA-processing factor 40 homolog A OS=Mus musculus GN=Prpf40a PE=1 SV=1 | 11 | 0.946 | 0.000 | 1.747 |
| sp|Q8K4L4|POF1B_MOUSE | Protein POF1B OS=Mus musculus GN=Pof1b PE=1 SV=3 | 11 | 0.964 | 0.913 | 1.824 |
| sp|Q9CQC7|NDUB4_MOUSE | NADH dehydrogenase [ubiquinone] 1 beta subcomplex subunit 4 OS=Mus musculus GN=Ndufb4 PE=1 SV=3 | 11 | 0.852 | 0.819 | 1.851 |
| sp|P24668|MPRD_MOUSE | Cation-dependent mannose-6-phosphate receptor OS=Mus musculus GN=M6pr PE=1 SV=1 | 11 | 1.019 | 0.000 | InD |
| sp|Q9R0Q3|TMED2_MOUSE | Transmembrane emp24 domain-containing protein 2 OS=Mus musculus GN=Tmed2 PE=1 SV=1 | 11 | 1.194 | 0.000 | InD |
| sp|P63044|VAMP2_MOUSE | Vesicle-associated membrane protein 2 OS=Mus musculus GN=Vamp2 PE=1 SV=2 | 11 | 1.040 | 0.769 | InD |
| sp|Q8BGS2|BOLA2_MOUSE | BolA-like protein 2 OS=Mus musculus GN=Bola2 PE=1 SV=1 | 11 | 1.235 | 0.905 | InD |
| sp|Q9CPP0|NPM3_MOUSE | Nucleoplasmin-3 OS=Mus musculus GN=Npm3 PE=2 SV=3 | 11 | 1.067 | 0.927 | InD |
| sp|Q6ZQI3|MLEC_MOUSE | Malectin OS=Mus musculus GN=Mlec PE=2 SV=2 | 11 | 0.000 | 0.952 | InD |
| sp|Q921E2|RAB31_MOUSE | Ras-related protein Rab-31 OS=Mus musculus GN=Rab31 PE=1 SV=1 | 11 | 1.071 | 0.955 | InD |
| sp|P62843|RS15_MOUSE | 40S ribosomal protein S15 OS=Mus musculus GN=Rps15 PE=2 SV=2 | 11 | 1.127 | 0.976 | InD |
| sp|Q3TTY5|K22E_MOUSE | Keratin, type II cytoskeletal 2 epidermal OS=Mus musculus GN=Krt2 PE=1 SV=1 | 11 | 0.000 | 1.020 | InD |
| sp|Q3UV17|K22O_MOUSE | Keratin, type II cytoskeletal 2 oral OS=Mus musculus GN=Krt76 PE=2 SV=1 | 11 | InD | 1.143 | InD |
| sp|Q9CRB3|HIUH_MOUSE | 5-hydroxyisourate hydrolase OS=Mus musculus GN=Urah PE=1 SV=1 | 11 | 1.414 | 1.349 | InD |
| sp|Q9CPT4|CS010_MOUSE | UPF0556 protein C19orf10 homolog OS=Mus musculus GN=D17Wsu104e PE=2 SV=1 | 11 | 0.782 | InD | InD |
| sp|P30285|CDK4_MOUSE | Cyclin-dependent kinase 4 OS=Mus musculus GN=Cdk4 PE=1 SV=1 | 11 | 0.988 | InD | InD |
| sp|Q8R146|APEH_MOUSE | Acylamino-acid-releasing enzyme OS=Mus musculus GN=Apeh PE=2 SV=3 | 10 | 1.130 | 0.000 | 0.000 |
| sp|Q99LD4|CSN1_MOUSE | COP9 signalosome complex subunit 1 OS=Mus musculus GN=Gps1 PE=1 SV=1 | 10 | 1.165 | 0.904 | 0.000 |
| sp|Q9QZD9|EIF3I_MOUSE | Eukaryotic translation initiation factor 3 subunit I OS=Mus musculus GN=Eif3i PE=1 SV=1 | 10 | 0.957 | 1.055 | 0.000 |
| sp|Q9EQP2|EHD4_MOUSE | EH domain-containing protein 4 OS=Mus musculus GN=Ehd4 PE=1 SV=1 | 10 | 0.000 | 1.069 | 0.000 |
| sp|B2RRE7|OTUD4_MOUSE | OTU domain-containing protein 4 OS=Mus musculus GN=Otud4 PE=1 SV=1 | 10 | 0.929 | 1.106 | 0.000 |
| sp|Q8VE37|RCC1_MOUSE | Regulator of chromosome condensation OS=Mus musculus GN=Rcc1 PE=1 SV=1 | 10 | 0.000 | 1.541 | 0.000 |
| sp|P57016|LAD1_MOUSE | Ladinin-1 OS=Mus musculus GN=Lad1 PE=1 SV=1 | 10 | 1.000 | 1.898 | 0.000 |
| sp|Q99JX3|GORS2_MOUSE | Golgi reassembly-stacking protein 2 OS=Mus musculus GN=Gorasp2 PE=1 SV=3 | 10 | 0.992 | InD | 0.000 |
| sp|P58281|OPA1_MOUSE | Dynamin-like 120 kDa protein, mitochondrial OS=Mus musculus GN=Opa1 PE=1 SV=1 | 10 | 1.058 | InD | 0.000 |
| sp|Q99KH8|STK24_MOUSE | Serine/threonine-protein kinase 24 OS=Mus musculus GN=Stk24 PE=1 SV=1 | 10 | 1.156 | InD | 0.000 |
| sp|Q8BXA1|GOLI4_MOUSE | Golgi integral membrane protein 4 OS=Mus musculus GN=Golim4 PE=1 SV=1 | 10 | 1.301 | InD | 0.000 |
| sp|G5E870|TRIPC_MOUSE | E3 ubiquitin-protein ligase TRIP12 OS=Mus musculus GN=Trip12 PE=1 SV=1 | 10 | 0.000 | 0.831 | 0.536 |
| sp|Q505F5|LRC47_MOUSE | Leucine-rich repeat-containing protein 47 OS=Mus musculus GN=Lrrc47 PE=1 SV=1 | 10 | 1.283 | 0.984 | 0.696 |
| sp|Q8BIJ6|SYIM_MOUSE | Isoleucine--tRNA ligase, mitochondrial OS=Mus musculus GN=Iars2 PE=2 SV=1 | 10 | 1.019 | 0.994 | 0.723 |
| sp|Q9Z120|TRMB_MOUSE | tRNA (guanine-N(7)-)-methyltransferase OS=Mus musculus GN=Mettl1 PE=2 SV=1 | 10 | 1.099 | 0.989 | 0.738 |
| sp|Q61990|PCBP2_MOUSE | Poly(rC)-binding protein 2 OS=Mus musculus GN=Pcbp2 PE=1 SV=1 | 10 | 0.000 | 1.008 | 0.752 |
| sp|P16332|MUTA_MOUSE | Methylmalonyl-CoA mutase, mitochondrial OS=Mus musculus GN=Mut PE=2 SV=2 | 10 | 1.348 | 0.919 | 0.769 |
| sp|Q8R1Q8|DC1L1_MOUSE | Cytoplasmic dynein 1 light intermediate chain 1 OS=Mus musculus GN=Dync1li1 PE=1 SV=1 | 10 | 1.412 | 1.032 | 0.783 |
| sp|Q9D967|MGDP1_MOUSE | Magnesium-dependent phosphatase 1 OS=Mus musculus GN=Mdp1 PE=1 SV=1 | 10 | 1.179 | 0.921 | 0.809 |
| sp|Q9D0F9|PGM1_MOUSE | Phosphoglucomutase-1 OS=Mus musculus GN=Pgm1 PE=1 SV=4 | 10 | InD | 1.125 | 0.837 |
| sp|Q9DCZ4|APOO_MOUSE | Apolipoprotein O OS=Mus musculus GN=Apoo PE=2 SV=1 | 10 | 1.000 | 0.932 | 0.865 |
| sp|P36536|SAR1A_MOUSE | GTP-binding protein SAR1a OS=Mus musculus GN=Sar1a PE=2 SV=1 | 10 | 1.302 | InD | 0.868 |
| sp|Q9CRA5|GOLP3_MOUSE | Golgi phosphoprotein 3 OS=Mus musculus GN=Golph3 PE=2 SV=1 | 10 | InD | 1.072 | 0.871 |
| sp|Q80Y14|GLRX5_MOUSE | Glutaredoxin-related protein 5, mitochondrial OS=Mus musculus GN=Glrx5 PE=2 SV=2 | 10 | 1.055 | 0.927 | 0.879 |
| sp|Q91W90|TXND5_MOUSE | Thioredoxin domain-containing protein 5 OS=Mus musculus GN=Txndc5 PE=1 SV=2 | 10 | 0.750 | InD | 0.881 |
| sp|Q9WV54|ASAH1_MOUSE | Acid ceramidase OS=Mus musculus GN=Asah1 PE=1 SV=1 | 10 | 1.022 | 1.011 | 0.889 |
| sp|O70493|SNX12_MOUSE | Sorting nexin-12 OS=Mus musculus GN=Snx12 PE=2 SV=1 | 10 | 1.168 | 1.000 | 0.934 |
| sp|Q62093|SRSF2_MOUSE | Serine/arginine-rich splicing factor 2 OS=Mus musculus GN=Srsf2 PE=1 SV=4 | 10 | 1.253 | 0.952 | 0.937 |
| sp|Q8CGC6|RBM28_MOUSE | RNA-binding protein 28 OS=Mus musculus GN=Rbm28 PE=1 SV=4 | 10 | 0.818 | 1.000 | 0.949 |
| sp|Q9Z2I9|SUCB1_MOUSE | Succinyl-CoA ligase [ADP-forming] subunit beta, mitochondrial OS=Mus musculus GN=Sucla2 PE=1 SV=2 | 10 | 1.155 | 0.730 | 0.964 |
| sp|Q91YE6|IPO9_MOUSE | Importin-9 OS=Mus musculus GN=Ipo9 PE=1 SV=3 | 10 | 0.907 | InD | 0.978 |
| sp|Q62189|SNRPA_MOUSE | U1 small nuclear ribonucleoprotein A OS=Mus musculus GN=Snrpa PE=2 SV=3 | 10 | 0.822 | 0.954 | 0.983 |
| sp|Q8BJS4|SUN2_MOUSE | SUN domain-containing protein 2 OS=Mus musculus GN=Sun2 PE=1 SV=3 | 10 | 0.902 | 0.751 | 1.000 |
| sp|Q6IRU5|CLCB_MOUSE | Clathrin light chain B OS=Mus musculus GN=Cltb PE=2 SV=1 | 10 | 0.000 | 0.982 | 1.000 |
| sp|Q99LD9|EI2BB_MOUSE | Translation initiation factor eIF-2B subunit beta OS=Mus musculus GN=Eif2b2 PE=2 SV=1 | 10 | 1.303 | 1.366 | 1.009 |
| sp|Q99J36|THUM1_MOUSE | THUMP domain-containing protein 1 OS=Mus musculus GN=Thumpd1 PE=1 SV=1 | 10 | 0.929 | 0.846 | 1.017 |
| sp|P68181|KAPCB_MOUSE | cAMP-dependent protein kinase catalytic subunit beta OS=Mus musculus GN=Prkacb PE=1 SV=2 | 10 | 0.000 | 1.179 | 1.018 |
| sp|Q6P8M1|TATD1_MOUSE | Putative deoxyribonuclease TATDN1 OS=Mus musculus GN=Tatdn1 PE=2 SV=1 | 10 | 1.211 | 0.000 | 1.075 |
| sp|A2AGT5|CKAP5_MOUSE | Cytoskeleton-associated protein 5 OS=Mus musculus GN=Ckap5 PE=2 SV=1 | 10 | 0.812 | InD | 1.092 |
| sp|P47713|PA24A_MOUSE | Cytosolic phospholipase A2 OS=Mus musculus GN=Pla2g4a PE=1 SV=1 | 10 | 1.139 | 1.193 | 1.211 |
| sp|Q8CI11|GNL3_MOUSE | Guanine nucleotide-binding protein-like 3 OS=Mus musculus GN=Gnl3 PE=1 SV=2 | 10 | 0.000 | 1.306 | 1.386 |
| sp|Q8QZS1|HIBCH_MOUSE | 3-hydroxyisobutyryl-CoA hydrolase, mitochondrial OS=Mus musculus GN=Hibch PE=1 SV=1 | 10 | 0.841 | InD | 1.443 |
| sp|P60824|CIRBP_MOUSE | Cold-inducible RNA-binding protein OS=Mus musculus GN=Cirbp PE=1 SV=1 | 10 | 0.850 | 0.875 | InD |
| sp|Q9CQ22|LTOR1_MOUSE | Ragulator complex protein LAMTOR1 OS=Mus musculus GN=Lamtor1 PE=1 SV=1 | 10 | 1.045 | 0.884 | InD |
| sp|O08800|SPB8_MOUSE | Serpin B8 OS=Mus musculus GN=Serpinb8 PE=2 SV=2 | 10 | 1.422 | 0.909 | InD |
| sp|P32883|RASK_MOUSE | GTPase KRas OS=Mus musculus GN=Kras PE=1 SV=1 | 10 | InD | 0.950 | InD |
| sp|P84089|ERH_MOUSE | Enhancer of rudimentary homolog OS=Mus musculus GN=Erh PE=1 SV=1 | 10 | 1.029 | 0.987 | InD |
| sp|Q9R0H0|ACOX1_MOUSE | Peroxisomal acyl-coenzyme A oxidase 1 OS=Mus musculus GN=Acox1 PE=1 SV=5 | 10 | 1.180 | 1.012 | InD |
| sp|P56391|CX6B1_MOUSE | Cytochrome c oxidase subunit 6B1 OS=Mus musculus GN=Cox6b1 PE=1 SV=2 | 10 | 0.874 | 1.034 | InD |
| sp|Q3V1L4|5NTC_MOUSE | Cytosolic purine 5&#039;-nucleotidase OS=Mus musculus GN=Nt5c2 PE=1 SV=2 | 10 | 0.000 | 1.043 | InD |
| sp|P48428|TBCA_MOUSE | Tubulin-specific chaperone A OS=Mus musculus GN=Tbca PE=2 SV=3 | 10 | 1.067 | 1.044 | InD |
| sp|P12367|KAP2_MOUSE | cAMP-dependent protein kinase type II-alpha regulatory subunit OS=Mus musculus GN=Prkar2a PE=1 SV=2 | 10 | 0.794 | 1.064 | InD |
| sp|Q8K3C3|LZIC_MOUSE | Protein LZIC OS=Mus musculus GN=Lzic PE=1 SV=1 | 10 | InD | 1.138 | InD |
| sp|Q9D1L0|CHCH2_MOUSE | Coiled-coil-helix-coiled-coil-helix domain-containing protein 2, mitochondrial OS=Mus musculus GN=Chchd2 PE=1 SV=1 | 10 | 2.193 | 1.289 | InD |
| sp|Q05816|FABP5_MOUSE | Fatty acid-binding protein, epidermal OS=Mus musculus GN=Fabp5 PE=1 SV=3 | 10 | 1.248 | InD | InD |
| sp|Q9JHU2|PALMD_MOUSE | Palmdelphin OS=Mus musculus GN=Palmd PE=1 SV=1 | 9 | 0.943 | 0.000 | 0.000 |
| sp|Q80U93|NU214_MOUSE | Nuclear pore complex protein Nup214 OS=Mus musculus GN=Nup214 PE=1 SV=2 | 9 | 1.104 | 0.000 | 0.000 |
| sp|Q5SF07|IF2B2_MOUSE | Insulin-like growth factor 2 mRNA-binding protein 2 OS=Mus musculus GN=Igf2bp2 PE=1 SV=1 | 9 | InD | 0.000 | 0.000 |
| sp|Q9JLN9|MTOR_MOUSE | Serine/threonine-protein kinase mTOR OS=Mus musculus GN=Mtor PE=1 SV=2 | 9 | 1.121 | 0.764 | 0.000 |
| sp|Q5XKN4|JAGN1_MOUSE | Protein jagunal homolog 1 OS=Mus musculus GN=Jagn1 PE=2 SV=2 | 9 | 0.932 | 0.858 | 0.000 |
| sp|Q9QZQ1|AFAD_MOUSE | Afadin OS=Mus musculus GN=Mllt4 PE=1 SV=3 | 9 | 1.747 | 0.871 | 0.000 |
| sp|Q64105|SPRE_MOUSE | Sepiapterin reductase OS=Mus musculus GN=Spr PE=1 SV=1 | 9 | 1.514 | 0.880 | 0.000 |
| sp|P00416|COX3_MOUSE | Cytochrome c oxidase subunit 3 OS=Mus musculus GN=Mtco3 PE=3 SV=1 | 9 | 1.095 | 0.899 | 0.000 |
| sp|Q9R112|SQRD_MOUSE | Sulfide:quinone oxidoreductase, mitochondrial OS=Mus musculus GN=Sqrdl PE=2 SV=3 | 9 | 1.288 | 0.910 | 0.000 |
| sp|Q78IK2|USMG5_MOUSE | Up-regulated during skeletal muscle growth protein 5 OS=Mus musculus GN=Usmg5 PE=1 SV=1 | 9 | 1.050 | 0.926 | 0.000 |
| sp|Q9QZ23|NFU1_MOUSE | NFU1 iron-sulfur cluster scaffold homolog, mitochondrial OS=Mus musculus GN=Nfu1 PE=1 SV=2 | 9 | 1.119 | 0.972 | 0.000 |
| sp|Q9DB27|MCTS1_MOUSE | Malignant T-cell-amplified sequence 1 OS=Mus musculus GN=Mcts1 PE=2 SV=1 | 9 | 1.081 | 1.076 | 0.000 |
| sp|P32921|SYWC_MOUSE | Tryptophan--tRNA ligase, cytoplasmic OS=Mus musculus GN=Wars PE=1 SV=2 | 9 | 1.156 | 1.214 | 0.000 |
| sp|P97384|ANX11_MOUSE | Annexin A11 OS=Mus musculus GN=Anxa11 PE=1 SV=2 | 9 | InD | 1.408 | 0.439 |
| sp|Q9R1A9|TREX2_MOUSE | Three prime repair exonuclease 2 OS=Mus musculus GN=Trex2 PE=2 SV=1 | 9 | 0.469 | 0.486 | 0.494 |
| sp|P45952|ACADM_MOUSE | Medium-chain specific acyl-CoA dehydrogenase, mitochondrial OS=Mus musculus GN=Acadm PE=1 SV=1 | 9 | 0.966 | 0.879 | 0.522 |
| sp|Q8K2Q7|BROX_MOUSE | BRO1 domain-containing protein BROX OS=Mus musculus GN=Brox PE=2 SV=1 | 9 | 0.824 | 0.842 | 0.615 |
| sp|P35285|RB22A_MOUSE | Ras-related protein Rab-22A OS=Mus musculus GN=Rab22a PE=1 SV=2 | 9 | 0.855 | 1.032 | 0.650 |
| sp|Q06138|CAB39_MOUSE | Calcium-binding protein 39 OS=Mus musculus GN=Cab39 PE=1 SV=2 | 9 | 0.905 | 0.000 | 0.670 |
| sp|Q61239|FNTA_MOUSE | Protein farnesyltransferase/geranylgeranyltransferase type-1 subunit alpha OS=Mus musculus GN=Fnta PE=1 SV=1 | 9 | 0.842 | 0.717 | 0.685 |
| sp|Q8BH69|SPS1_MOUSE | Selenide, water dikinase 1 OS=Mus musculus GN=Sephs1 PE=2 SV=1 | 9 | 1.269 | 0.821 | 0.691 |
| sp|Q9CRD2|EMC2_MOUSE | ER membrane protein complex subunit 2 OS=Mus musculus GN=Emc2 PE=2 SV=1 | 9 | 0.000 | 0.980 | 0.707 |
| sp|Q3TLH4|PRC2C_MOUSE | Protein PRRC2C OS=Mus musculus GN=Prrc2c PE=1 SV=3 | 9 | InD | InD | 0.736 |
| sp|Q9R0M6|RAB9A_MOUSE | Ras-related protein Rab-9A OS=Mus musculus GN=Rab9a PE=1 SV=1 | 9 | InD | 1.450 | 0.755 |
| sp|Q80UM7|MOGS_MOUSE | Mannosyl-oligosaccharide glucosidase OS=Mus musculus GN=Mogs PE=2 SV=1 | 9 | InD | InD | 0.764 |
| sp|Q3TRM4|PLPL6_MOUSE | Neuropathy target esterase OS=Mus musculus GN=Pnpla6 PE=2 SV=2 | 9 | InD | 1.011 | 0.815 |
| sp|Q9CZR8|EFTS_MOUSE | Elongation factor Ts, mitochondrial OS=Mus musculus GN=Tsfm PE=2 SV=1 | 9 | 1.174 | 1.102 | 0.837 |
| sp|Q8R0X7|SGPL1_MOUSE | Sphingosine-1-phosphate lyase 1 OS=Mus musculus GN=Sgpl1 PE=2 SV=1 | 9 | 0.848 | InD | 0.839 |
| sp|Q3TCH7|CUL4A_MOUSE | Cullin-4A OS=Mus musculus GN=Cul4a PE=1 SV=1 | 9 | 0.000 | InD | 0.852 |
| sp|P99026|PSB4_MOUSE | Proteasome subunit beta type-4 OS=Mus musculus GN=Psmb4 PE=1 SV=1 | 9 | 0.755 | 0.000 | 0.858 |
| sp|Q99LB6|MAT2B_MOUSE | Methionine adenosyltransferase 2 subunit beta OS=Mus musculus GN=Mat2b PE=2 SV=1 | 9 | 0.000 | 0.000 | 0.911 |
| sp|Q9D2V8|MFS10_MOUSE | Major facilitator superfamily domain-containing protein 10 OS=Mus musculus GN=Mfsd10 PE=2 SV=1 | 9 | 0.000 | 0.705 | 0.915 |
| sp|Q8BHE8|CB047_MOUSE | Uncharacterized protein C2orf47 homolog, mitochondrial OS=Mus musculus PE=2 SV=1 | 9 | InD | 0.631 | 0.922 |
| sp|P28660|NCKP1_MOUSE | Nck-associated protein 1 OS=Mus musculus GN=Nckap1 PE=1 SV=2 | 9 | 1.252 | InD | 0.926 |
| sp|Q5M8N4|D39U1_MOUSE | Epimerase family protein SDR39U1 OS=Mus musculus GN=Sdr39u1 PE=1 SV=1 | 9 | 1.254 | InD | 0.928 |
| sp|Q9EST1|GSDMA_MOUSE | Gasdermin-A OS=Mus musculus GN=Gsdma PE=2 SV=1 | 9 | 0.989 | 0.894 | 0.934 |
| sp|P17439|GLCM_MOUSE | Glucosylceramidase OS=Mus musculus GN=Gba PE=1 SV=1 | 9 | 1.009 | 0.814 | 0.944 |
| sp|P70699|LYAG_MOUSE | Lysosomal alpha-glucosidase OS=Mus musculus GN=Gaa PE=1 SV=2 | 9 | 0.873 | 0.812 | 0.980 |
| sp|Q9DC61|MPPA_MOUSE | Mitochondrial-processing peptidase subunit alpha OS=Mus musculus GN=Pmpca PE=1 SV=1 | 9 | 1.145 | InD | 0.990 |
| sp|P70460|VASP_MOUSE | Vasodilator-stimulated phosphoprotein OS=Mus musculus GN=Vasp PE=1 SV=4 | 9 | 1.417 | 1.168 | 1.019 |
| sp|O55101|SNG2_MOUSE | Synaptogyrin-2 OS=Mus musculus GN=Syngr2 PE=2 SV=1 | 9 | 1.085 | 0.901 | 1.024 |
| sp|Q91ZU6|DYST_MOUSE | Dystonin OS=Mus musculus GN=Dst PE=1 SV=1 | 9 | 1.868 | InD | 1.060 |
| sp|Q9CQC6|BZW1_MOUSE | Basic leucine zipper and W2 domain-containing protein 1 OS=Mus musculus GN=Bzw1 PE=1 SV=1 | 9 | 1.213 | 0.000 | 1.061 |
| sp|Q9CXE7|TMED5_MOUSE | Transmembrane emp24 domain-containing protein 5 OS=Mus musculus GN=Tmed5 PE=2 SV=1 | 9 | InD | 0.000 | 1.074 |
| sp|Q3U9G9|LBR_MOUSE | Lamin-B receptor OS=Mus musculus GN=Lbr PE=1 SV=2 | 9 | 1.110 | InD | 1.081 |
| sp|P61022|CHP1_MOUSE | Calcineurin B homologous protein 1 OS=Mus musculus GN=Chp1 PE=1 SV=2 | 9 | 0.000 | 0.863 | 1.128 |
| sp|Q91WS0|CISD1_MOUSE | CDGSH iron-sulfur domain-containing protein 1 OS=Mus musculus GN=Cisd1 PE=1 SV=1 | 9 | 0.850 | 0.000 | 1.162 |
| sp|P28667|MRP_MOUSE | MARCKS-related protein OS=Mus musculus GN=Marcksl1 PE=1 SV=2 | 9 | 0.992 | 1.821 | 1.222 |
| sp|Q9DC50|OCTC_MOUSE | Peroxisomal carnitine O-octanoyltransferase OS=Mus musculus GN=Crot PE=1 SV=1 | 9 | 1.467 | 0.932 | 1.307 |
| sp|Q9DCL8|IPP2_MOUSE | Protein phosphatase inhibitor 2 OS=Mus musculus GN=Ppp1r2 PE=1 SV=3 | 9 | InD | 1.436 | 1.528 |
| sp|P84075|HPCA_MOUSE | Neuron-specific calcium-binding protein hippocalcin OS=Mus musculus GN=Hpca PE=1 SV=2 | 9 | InD | 0.000 | 1.674 |
| sp|O89079|COPE_MOUSE | Coatomer subunit epsilon OS=Mus musculus GN=Cope PE=2 SV=3 | 9 | 0.962 | 0.000 | InD |
| sp|Q9CX00|IST1_MOUSE | IST1 homolog OS=Mus musculus GN=Ist1 PE=2 SV=1 | 9 | 1.124 | 0.882 | InD |
| sp|Q9CPU2|NDUB2_MOUSE | NADH dehydrogenase [ubiquinone] 1 beta subcomplex subunit 2, mitochondrial OS=Mus musculus GN=Ndufb2 PE=2 SV=1 | 9 | 0.821 | 0.938 | InD |
| sp|O09174|AMACR_MOUSE | Alpha-methylacyl-CoA racemase OS=Mus musculus GN=Amacr PE=1 SV=4 | 9 | 1.046 | 1.101 | InD |
| sp|Q9D0J4|ARL2_MOUSE | ADP-ribosylation factor-like protein 2 OS=Mus musculus GN=Arl2 PE=1 SV=1 | 9 | 0.000 | 1.120 | InD |
| sp|Q8BH15|CNOTA_MOUSE | CCR4-NOT transcription complex subunit 10 OS=Mus musculus GN=Cnot10 PE=2 SV=1 | 9 | 0.855 | 1.243 | InD |
| sp|Q9QZQ8|H2AY_MOUSE | Core histone macro-H2A.1 OS=Mus musculus GN=H2afy PE=1 SV=3 | 9 | 0.773 | InD | InD |
| sp|P25976|UBF1_MOUSE | Nucleolar transcription factor 1 OS=Mus musculus GN=Ubtf PE=1 SV=1 | 9 | 1.083 | InD | InD |
| sp|Q8C166|CPNE1_MOUSE | Copine-1 OS=Mus musculus GN=Cpne1 PE=1 SV=1 | 8 | 0.000 | 0.000 | 0.000 |
| sp|Q9D8S9|BOLA1_MOUSE | BolA-like protein 1 OS=Mus musculus GN=Bola1 PE=1 SV=1 | 8 | 1.198 | 0.000 | 0.000 |
| sp|Q99KB8|GLO2_MOUSE | Hydroxyacylglutathione hydrolase, mitochondrial OS=Mus musculus GN=Hagh PE=2 SV=2 | 8 | 1.000 | 0.742 | 0.000 |
| sp|Q91V61|SFXN3_MOUSE | Sideroflexin-3 OS=Mus musculus GN=Sfxn3 PE=1 SV=1 | 8 | InD | 0.877 | 0.000 |
| sp|Q9WUM5|SUCA_MOUSE | Succinyl-CoA ligase [ADP/GDP-forming] subunit alpha, mitochondrial OS=Mus musculus GN=Suclg1 PE=1 SV=4 | 8 | 1.034 | 0.897 | 0.000 |
| sp|Q9JIA7|SPHK2_MOUSE | Sphingosine kinase 2 OS=Mus musculus GN=Sphk2 PE=1 SV=2 | 8 | InD | 0.914 | 0.000 |
| sp|Q11136|PEPD_MOUSE | Xaa-Pro dipeptidase OS=Mus musculus GN=Pepd PE=2 SV=3 | 8 | 0.000 | 0.942 | 0.000 |
| sp|Q99LX0|PARK7_MOUSE | Protein DJ-1 OS=Mus musculus GN=Park7 PE=1 SV=1 | 8 | 0.000 | 0.952 | 0.000 |
| sp|Q9EQ20|MMSA_MOUSE | Methylmalonate-semialdehyde dehydrogenase [acylating], mitochondrial OS=Mus musculus GN=Aldh6a1 PE=1 SV=1 | 8 | 1.247 | 1.102 | 0.000 |
| sp|Q9CPS7|PNO1_MOUSE | RNA-binding protein PNO1 OS=Mus musculus GN=Pno1 PE=2 SV=1 | 8 | 1.367 | 1.196 | 0.000 |
| sp|Q9R0K7|AT2B2_MOUSE | Plasma membrane calcium-transporting ATPase 2 OS=Mus musculus GN=Atp2b2 PE=1 SV=2 | 8 | 0.496 | 2.204 | 0.000 |
| sp|Q60967|PAPS1_MOUSE | Bifunctional 3&#039;-phosphoadenosine 5&#039;-phosphosulfate synthase 1 OS=Mus musculus GN=Papss1 PE=2 SV=1 | 8 | 0.107 | InD | 0.000 |
| sp|Q9DBE9|RRMJ3_MOUSE | pre-rRNA processing protein FTSJ3 OS=Mus musculus GN=Ftsj3 PE=1 SV=1 | 8 | 0.970 | InD | 0.000 |
| sp|Q6DIC0|SMCA2_MOUSE | Probable global transcription activator SNF2L2 OS=Mus musculus GN=Smarca2 PE=1 SV=1 | 8 | 1.054 | InD | 0.000 |
| sp|Q9D1Q6|ERP44_MOUSE | Endoplasmic reticulum resident protein 44 OS=Mus musculus GN=Erp44 PE=1 SV=1 | 8 | 1.096 | InD | 0.000 |
| sp|Q91VW5|GOGA4_MOUSE | Golgin subfamily A member 4 OS=Mus musculus GN=Golga4 PE=1 SV=2 | 8 | 1.196 | InD | 0.000 |
| sp|Q6Q899|DDX58_MOUSE | Probable ATP-dependent RNA helicase DDX58 OS=Mus musculus GN=Ddx58 PE=1 SV=2 | 8 | 1.405 | InD | 0.000 |
| sp|Q61249|IGBP1_MOUSE | Immunoglobulin-binding protein 1 OS=Mus musculus GN=Igbp1 PE=1 SV=1 | 8 | 0.990 | 0.162 | 0.224 |
| sp|Q8CEZ4|CB054_MOUSE | Uncharacterized protein C2orf54 homolog OS=Mus musculus PE=2 SV=2 | 8 | 0.635 | InD | 0.272 |
| sp|P57096|PSCA_MOUSE | Prostate stem cell antigen OS=Mus musculus GN=Psca PE=2 SV=1 | 8 | 0.000 | 0.697 | 0.488 |
| sp|P18828|SDC1_MOUSE | Syndecan-1 OS=Mus musculus GN=Sdc1 PE=1 SV=1 | 8 | 0.531 | InD | 0.525 |
| sp|P17809|GTR1_MOUSE | Solute carrier family 2, facilitated glucose transporter member 1 OS=Mus musculus GN=Slc2a1 PE=1 SV=4 | 8 | InD | InD | 0.591 |
| sp|Q8BU14|SEC62_MOUSE | Translocation protein SEC62 OS=Mus musculus GN=Sec62 PE=1 SV=1 | 8 | 0.000 | 1.144 | 0.655 |
| sp|Q8VDP4|K1967_MOUSE | DBIRD complex subunit KIAA1967 homolog OS=Mus musculus PE=1 SV=2 | 8 | 0.000 | InD | 0.705 |
| sp|P19536|COX5B_MOUSE | Cytochrome c oxidase subunit 5B, mitochondrial OS=Mus musculus GN=Cox5b PE=1 SV=1 | 8 | 1.209 | 1.218 | 0.716 |
| sp|Q7TMF3|NDUAC_MOUSE | NADH dehydrogenase [ubiquinone] 1 alpha subcomplex subunit 12 OS=Mus musculus GN=Ndufa12 PE=1 SV=2 | 8 | 1.042 | 0.882 | 0.753 |
| sp|P56376|ACYP1_MOUSE | Acylphosphatase-1 OS=Mus musculus GN=Acyp1 PE=2 SV=2 | 8 | 1.165 | 0.879 | 0.781 |
| sp|Q61532|MK06_MOUSE | Mitogen-activated protein kinase 6 OS=Mus musculus GN=Mapk6 PE=1 SV=3 | 8 | InD | 0.000 | 0.806 |
| sp|Q9DC51|GNAI3_MOUSE | Guanine nucleotide-binding protein G(k) subunit alpha OS=Mus musculus GN=Gnai3 PE=1 SV=3 | 8 | InD | 0.000 | 0.808 |
| sp|Q99KE1|MAOM_MOUSE | NAD-dependent malic enzyme, mitochondrial OS=Mus musculus GN=Me2 PE=2 SV=1 | 8 | InD | InD | 0.816 |
| sp|Q63844|MK03_MOUSE | Mitogen-activated protein kinase 3 OS=Mus musculus GN=Mapk3 PE=1 SV=5 | 8 | InD | 0.000 | 0.824 |
| sp|Q6P5G0|MK04_MOUSE | Mitogen-activated protein kinase 4 OS=Mus musculus GN=Mapk4 PE=1 SV=1 | 8 | InD | 0.000 | 0.824 |
| sp|Q9CY50|SSRA_MOUSE | Translocon-associated protein subunit alpha OS=Mus musculus GN=Ssr1 PE=1 SV=1 | 8 | InD | 1.326 | 0.825 |
| sp|Q6A068|CDC5L_MOUSE | Cell division cycle 5-like protein OS=Mus musculus GN=Cdc5l PE=1 SV=2 | 8 | 0.000 | InD | 0.831 |
| sp|Q3UYV9|NCBP1_MOUSE | Nuclear cap-binding protein subunit 1 OS=Mus musculus GN=Ncbp1 PE=1 SV=2 | 8 | 0.958 | InD | 0.841 |
| sp|P70288|HDAC2_MOUSE | Histone deacetylase 2 OS=Mus musculus GN=Hdac2 PE=1 SV=1 | 8 | InD | 1.529 | 0.850 |
| sp|Q9CQR6|PPP6_MOUSE | Serine/threonine-protein phosphatase 6 catalytic subunit OS=Mus musculus GN=Ppp6c PE=2 SV=1 | 8 | 0.985 | 0.000 | 0.867 |
| sp|Q9EPK7|XPO7_MOUSE | Exportin-7 OS=Mus musculus GN=Xpo7 PE=2 SV=3 | 8 | InD | InD | 0.883 |
| sp|Q62203|SF3A2_MOUSE | Splicing factor 3A subunit 2 OS=Mus musculus GN=Sf3a2 PE=2 SV=2 | 8 | InD | InD | 0.899 |
| sp|Q9DCS2|CP013_MOUSE | UPF0585 protein C16orf13 homolog OS=Mus musculus PE=1 SV=1 | 8 | 0.829 | InD | 0.913 |
| sp|O88545|CSN6_MOUSE | COP9 signalosome complex subunit 6 OS=Mus musculus GN=Cops6 PE=1 SV=1 | 8 | 0.000 | 1.277 | 0.917 |
| sp|Q9CQH3|NDUB5_MOUSE | NADH dehydrogenase [ubiquinone] 1 beta subcomplex subunit 5, mitochondrial OS=Mus musculus GN=Ndufb5 PE=1 SV=1 | 8 | 0.000 | InD | 0.927 |
| sp|Q64213|SF01_MOUSE | Splicing factor 1 OS=Mus musculus GN=Sf1 PE=1 SV=6 | 8 | 1.631 | InD | 0.983 |
| sp|Q8BUK6|HOOK3_MOUSE | Protein Hook homolog 3 OS=Mus musculus GN=Hook3 PE=1 SV=2 | 8 | InD | InD | 1.000 |
| sp|Q8R570|SNP47_MOUSE | Synaptosomal-associated protein 47 OS=Mus musculus GN=Snap47 PE=1 SV=1 | 8 | 1.183 | 1.201 | 1.036 |
| sp|Q02248|CTNB1_MOUSE | Catenin beta-1 OS=Mus musculus GN=Ctnnb1 PE=1 SV=1 | 8 | 0.000 | 1.429 | 1.053 |
| sp|Q80UU9|PGRC2_MOUSE | Membrane-associated progesterone receptor component 2 OS=Mus musculus GN=Pgrmc2 PE=1 SV=2 | 8 | 1.021 | 0.915 | 1.054 |
| sp|Q01730|RSU1_MOUSE | Ras suppressor protein 1 OS=Mus musculus GN=Rsu1 PE=2 SV=3 | 8 | 1.204 | InD | 1.061 |
| sp|Q9EQ61|PESC_MOUSE | Pescadillo homolog OS=Mus musculus GN=Pes1 PE=1 SV=1 | 8 | 0.000 | 0.912 | 1.103 |
| sp|Q8QZT1|THIL_MOUSE | Acetyl-CoA acetyltransferase, mitochondrial OS=Mus musculus GN=Acat1 PE=1 SV=1 | 8 | InD | 0.659 | 1.134 |
| sp|Q3TIX9|SNUT2_MOUSE | U4/U6.U5 tri-snRNP-associated protein 2 OS=Mus musculus GN=Usp39 PE=2 SV=2 | 8 | 0.000 | InD | 1.185 |
| sp|Q6EJB6|UT14B_MOUSE | U3 small nucleolar RNA-associated protein 14 homolog B OS=Mus musculus GN=Utp14b PE=1 SV=2 | 8 | 1.018 | 1.036 | 1.263 |
| sp|Q640M1|UT14A_MOUSE | U3 small nucleolar RNA-associated protein 14 homolog A OS=Mus musculus GN=Utp14a PE=2 SV=1 | 8 | 1.018 | 1.054 | 1.263 |
| sp|Q69Z37|SAM9L_MOUSE | Sterile alpha motif domain-containing protein 9-like OS=Mus musculus GN=Samd9l PE=2 SV=2 | 8 | 0.943 | InD | 1.312 |
| sp|Q07235|GDN_MOUSE | Glia-derived nexin OS=Mus musculus GN=Serpine2 PE=2 SV=2 | 8 | 1.495 | InD | 1.429 |
| sp|Q61335|BAP31_MOUSE | B-cell receptor-associated protein 31 OS=Mus musculus GN=Bcap31 PE=1 SV=4 | 8 | 1.704 | 0.981 | 1.667 |
| sp|Q8R3Q6|CCD58_MOUSE | Coiled-coil domain-containing protein 58 OS=Mus musculus GN=Ccdc58 PE=2 SV=1 | 8 | 1.024 | 0.000 | InD |
| sp|Q99KC8|VMA5A_MOUSE | von Willebrand factor A domain-containing protein 5A OS=Mus musculus GN=Vwa5a PE=1 SV=2 | 8 | 1.139 | 0.000 | InD |
| sp|Q923D4|SF3B5_MOUSE | Splicing factor 3B subunit 5 OS=Mus musculus GN=Sf3b5 PE=2 SV=1 | 8 | 0.619 | 0.733 | InD |
| sp|O88851|RBBP9_MOUSE | Putative hydrolase RBBP9 OS=Mus musculus GN=Rbbp9 PE=1 SV=2 | 8 | 1.231 | 0.886 | InD |
| sp|Q9DBG9|TX1B3_MOUSE | Tax1-binding protein 3 OS=Mus musculus GN=Tax1bp3 PE=1 SV=1 | 8 | 1.284 | 0.899 | InD |
| sp|Q9CQH7|BT3L4_MOUSE | Transcription factor BTF3 homolog 4 OS=Mus musculus GN=Btf3l4 PE=2 SV=1 | 8 | 1.161 | 0.907 | InD |
| sp|Q7TT50|MRCKB_MOUSE | Serine/threonine-protein kinase MRCK beta OS=Mus musculus GN=Cdc42bpb PE=1 SV=2 | 8 | 1.248 | 0.975 | InD |
| sp|Q8BIW1|PRUNE_MOUSE | Protein prune homolog OS=Mus musculus GN=Prune PE=2 SV=1 | 8 | 0.971 | 1.023 | InD |
| sp|Q61411|RASH_MOUSE | GTPase HRas OS=Mus musculus GN=Hras1 PE=1 SV=2 | 8 | 1.268 | 1.050 | InD |
| sp|Q9D3J5|ANR22_MOUSE | Ankyrin repeat domain-containing protein 22 OS=Mus musculus GN=Ankrd22 PE=2 SV=1 | 8 | 1.158 | 1.052 | InD |
| sp|P08556|RASN_MOUSE | GTPase NRas OS=Mus musculus GN=Nras PE=2 SV=1 | 8 | 1.268 | 1.057 | InD |
| sp|P83940|ELOC_MOUSE | Transcription elongation factor B polypeptide 1 OS=Mus musculus GN=Tceb1 PE=1 SV=1 | 8 | InD | 1.094 | InD |
| sp|Q922Y1|UBXN1_MOUSE | UBX domain-containing protein 1 OS=Mus musculus GN=Ubxn1 PE=1 SV=1 | 8 | InD | 1.329 | InD |
| sp|P54116|STOM_MOUSE | Erythrocyte band 7 integral membrane protein OS=Mus musculus GN=Stom PE=1 SV=3 | 8 | InD | 1.402 | InD |
| sp|O35459|ECH1_MOUSE | Delta(3,5)-Delta(2,4)-dienoyl-CoA isomerase, mitochondrial OS=Mus musculus GN=Ech1 PE=2 SV=1 | 8 | 1.078 | InD | InD |
| sp|Q9CRB2|NHP2_MOUSE | H/ACA ribonucleoprotein complex subunit 2 OS=Mus musculus GN=Nhp2 PE=2 SV=1 | 8 | 1.239 | InD | InD |
| sp|Q8CFE4|SCYL2_MOUSE | SCY1-like protein 2 OS=Mus musculus GN=Scyl2 PE=1 SV=1 | 8 | 1.329 | InD | InD |
| sp|P70336|ROCK2_MOUSE | Rho-associated protein kinase 2 OS=Mus musculus GN=Rock2 PE=1 SV=1 | 8 | 1.426 | InD | InD |
| sp|Q8BJW6|EIF2A_MOUSE | Eukaryotic translation initiation factor 2A OS=Mus musculus GN=Eif2a PE=2 SV=2 | 7 | 0.000 | 0.000 | 0.000 |
| sp|P29387|GBB4_MOUSE | Guanine nucleotide-binding protein subunit beta-4 OS=Mus musculus GN=Gnb4 PE=2 SV=4 | 7 | 0.949 | 0.000 | 0.000 |
| sp|Q08024|PEBB_MOUSE | Core-binding factor subunit beta OS=Mus musculus GN=Cbfb PE=1 SV=1 | 7 | 0.977 | 0.000 | 0.000 |
| sp|Q8CI94|PYGB_MOUSE | Glycogen phosphorylase, brain form OS=Mus musculus GN=Pygb PE=1 SV=3 | 7 | 1.279 | 0.000 | 0.000 |
| sp|Q8BKX1|BAIP2_MOUSE | Brain-specific angiogenesis inhibitor 1-associated protein 2 OS=Mus musculus GN=Baiap2 PE=1 SV=2 | 7 | 1.605 | 0.253 | 0.000 |
| sp|Q8BHL4|RAI3_MOUSE | Retinoic acid-induced protein 3 OS=Mus musculus GN=Gprc5a PE=2 SV=1 | 7 | InD | 0.864 | 0.000 |
| sp|Q91VR8|BRK1_MOUSE | Protein BRICK1 OS=Mus musculus GN=Brk1 PE=2 SV=1 | 7 | 1.235 | 0.922 | 0.000 |
| sp|Q9WVE8|PACN2_MOUSE | Protein kinase C and casein kinase substrate in neurons protein 2 OS=Mus musculus GN=Pacsin2 PE=1 SV=1 | 7 | 0.865 | 1.026 | 0.000 |
| sp|Q9JHS4|CLPX_MOUSE | ATP-dependent Clp protease ATP-binding subunit clpX-like, mitochondrial OS=Mus musculus GN=Clpx PE=1 SV=2 | 7 | 0.000 | 1.262 | 0.000 |
| sp|Q9CXW3|CYBP_MOUSE | Calcyclin-binding protein OS=Mus musculus GN=Cacybp PE=1 SV=1 | 7 | 1.259 | 1.534 | 0.000 |
| sp|Q6PDL0|DC1L2_MOUSE | Cytoplasmic dynein 1 light intermediate chain 2 OS=Mus musculus GN=Dync1li2 PE=1 SV=2 | 7 | 0.868 | InD | 0.000 |
| sp|Q9CQI3|GMFB_MOUSE | Glia maturation factor beta OS=Mus musculus GN=Gmfb PE=1 SV=3 | 7 | 1.088 | InD | 0.000 |
| sp|Q61210|ARHG1_MOUSE | Rho guanine nucleotide exchange factor 1 OS=Mus musculus GN=Arhgef1 PE=1 SV=2 | 7 | 1.316 | InD | 0.000 |
| sp|P62996|TRA2B_MOUSE | Transformer-2 protein homolog beta OS=Mus musculus GN=Tra2b PE=1 SV=1 | 7 | 0.000 | 0.000 | 0.355 |
| sp|O35344|IMA3_MOUSE | Importin subunit alpha-3 OS=Mus musculus GN=Kpna3 PE=1 SV=1 | 7 | 0.917 | InD | 0.516 |
| sp|Q3UMR5|MCU_MOUSE | Calcium uniporter protein, mitochondrial OS=Mus musculus GN=Mcu PE=2 SV=2 | 7 | 1.275 | InD | 0.680 |
| sp|P18654|KS6A3_MOUSE | Ribosomal protein S6 kinase alpha-3 OS=Mus musculus GN=Rps6ka3 PE=1 SV=2 | 7 | 2.099 | InD | 0.726 |
| sp|Q9Z0Y1|DCTN3_MOUSE | Dynactin subunit 3 OS=Mus musculus GN=Dctn3 PE=2 SV=2 | 7 | InD | 0.900 | 0.746 |
| sp|Q91YR1|TWF1_MOUSE | Twinfilin-1 OS=Mus musculus GN=Twf1 PE=1 SV=2 | 7 | 1.138 | InD | 0.750 |
| sp|Q8C8U0|LIPB1_MOUSE | Liprin-beta-1 OS=Mus musculus GN=Ppfibp1 PE=1 SV=3 | 7 | 0.861 | InD | 0.781 |
| sp|P62313|LSM6_MOUSE | U6 snRNA-associated Sm-like protein LSm6 OS=Mus musculus GN=Lsm6 PE=2 SV=1 | 7 | 0.000 | 0.837 | 0.793 |
| sp|Q8CD10|EFHA1_MOUSE | EF-hand domain-containing family member A1 OS=Mus musculus GN=Efha1 PE=2 SV=2 | 7 | 0.891 | InD | 0.807 |
| sp|Q3UGR5|HDHD2_MOUSE | Haloacid dehalogenase-like hydrolase domain-containing protein 2 OS=Mus musculus GN=Hdhd2 PE=1 SV=2 | 7 | 0.000 | InD | 0.811 |
| sp|Q0KL02|TRIO_MOUSE | Triple functional domain protein OS=Mus musculus GN=Trio PE=1 SV=3 | 7 | InD | 0.940 | 0.855 |
| sp|Q80VD1|FA98B_MOUSE | Protein FAM98B OS=Mus musculus GN=Fam98b PE=2 SV=1 | 7 | 0.000 | InD | 0.865 |
| sp|Q8BFQ8|PDDC1_MOUSE | Parkinson disease 7 domain-containing protein 1 OS=Mus musculus GN=Pddc1 PE=1 SV=1 | 7 | InD | 0.947 | 0.877 |
| sp|Q9CRB9|CHCH3_MOUSE | Coiled-coil-helix-coiled-coil-helix domain-containing protein 3, mitochondrial OS=Mus musculus GN=Chchd3 PE=1 SV=1 | 7 | 0.000 | 0.720 | 0.886 |
| sp|Q9D051|ODPB_MOUSE | Pyruvate dehydrogenase E1 component subunit beta, mitochondrial OS=Mus musculus GN=Pdhb PE=1 SV=1 | 7 | 0.962 | 0.000 | 0.894 |
| sp|Q9ERG2|STRN3_MOUSE | Striatin-3 OS=Mus musculus GN=Strn3 PE=1 SV=1 | 7 | InD | 0.000 | 0.911 |
| sp|P62311|LSM3_MOUSE | U6 snRNA-associated Sm-like protein LSm3 OS=Mus musculus GN=Lsm3 PE=2 SV=2 | 7 | 1.045 | 0.000 | 0.914 |
| sp|P50431|GLYC_MOUSE | Serine hydroxymethyltransferase, cytosolic OS=Mus musculus GN=Shmt1 PE=1 SV=3 | 7 | 1.169 | 1.224 | 0.917 |
| sp|Q8R180|ERO1A_MOUSE | ERO1-like protein alpha OS=Mus musculus GN=Ero1l PE=1 SV=2 | 7 | 0.000 | InD | 0.936 |
| sp|Q920A5|RISC_MOUSE | Retinoid-inducible serine carboxypeptidase OS=Mus musculus GN=Scpep1 PE=2 SV=2 | 7 | 0.898 | InD | 0.945 |
| sp|Q3UMB9|WASH7_MOUSE | WASH complex subunit 7 OS=Mus musculus GN=Kiaa1033 PE=2 SV=2 | 7 | 1.239 | InD | 0.945 |
| sp|Q8C878|UBA3_MOUSE | NEDD8-activating enzyme E1 catalytic subunit OS=Mus musculus GN=Uba3 PE=1 SV=2 | 7 | 1.036 | InD | 0.963 |
| sp|Q3TXU5|DHYS_MOUSE | Deoxyhypusine synthase OS=Mus musculus GN=Dhps PE=2 SV=2 | 7 | InD | 0.963 | 0.968 |
| sp|Q9D1H7|GET4_MOUSE | Golgi to ER traffic protein 4 homolog OS=Mus musculus GN=Get4 PE=2 SV=2 | 7 | 1.155 | 0.000 | 0.969 |
| sp|Q91VN4|CHCH6_MOUSE | Coiled-coil-helix-coiled-coil-helix domain-containing protein 6, mitochondrial OS=Mus musculus GN=Chchd6 PE=2 SV=2 | 7 | InD | 0.000 | 0.970 |
| sp|Q8C052|MAP1S_MOUSE | Microtubule-associated protein 1S OS=Mus musculus GN=Map1s PE=1 SV=2 | 7 | 0.000 | InD | 0.970 |
| sp|Q9CRT8|XPOT_MOUSE | Exportin-T OS=Mus musculus GN=Xpot PE=2 SV=3 | 7 | 0.000 | InD | 0.990 |
| sp|O35239|PTN9_MOUSE | Tyrosine-protein phosphatase non-receptor type 9 OS=Mus musculus GN=Ptpn9 PE=2 SV=2 | 7 | InD | 1.048 | 0.996 |
| sp|P06801|MAOX_MOUSE | NADP-dependent malic enzyme OS=Mus musculus GN=Me1 PE=1 SV=2 | 7 | InD | InD | 1.000 |
| sp|Q3U2P1|SC24A_MOUSE | Protein transport protein Sec24A OS=Mus musculus GN=Sec24a PE=1 SV=1 | 7 | 1.139 | InD | 1.041 |
| sp|Q9DBH5|LMAN2_MOUSE | Vesicular integral-membrane protein VIP36 OS=Mus musculus GN=Lman2 PE=2 SV=2 | 7 | 1.369 | 0.000 | 1.072 |
| sp|Q9D023|MPC2_MOUSE | Mitochondrial pyruvate carrier 2 OS=Mus musculus GN=Mpc2 PE=1 SV=1 | 7 | 0.000 | 0.953 | 1.202 |
| sp|Q922J9|FACR1_MOUSE | Fatty acyl-CoA reductase 1 OS=Mus musculus GN=Far1 PE=1 SV=1 | 7 | 1.022 | 1.171 | 1.250 |
| sp|Q8VCN5|CGL_MOUSE | Cystathionine gamma-lyase OS=Mus musculus GN=Cth PE=1 SV=1 | 7 | InD | InD | 1.333 |
| sp|P39749|FEN1_MOUSE | Flap endonuclease 1 OS=Mus musculus GN=Fen1 PE=1 SV=1 | 7 | 0.000 | 23.250 | 1.347 |
| sp|Q925B0|PAWR_MOUSE | PRKC apoptosis WT1 regulator protein OS=Mus musculus GN=Pawr PE=1 SV=2 | 7 | InD | 1.341 | 1.375 |
| sp|Q9DBS9|OSBL3_MOUSE | Oxysterol-binding protein-related protein 3 OS=Mus musculus GN=Osbpl3 PE=1 SV=2 | 7 | 0.981 | 0.000 | 1.632 |
| sp|Q9D855|QCR7_MOUSE | Cytochrome b-c1 complex subunit 7 OS=Mus musculus GN=Uqcrb PE=1 SV=3 | 7 | 1.201 | InD | 1.853 |
| sp|Q9D1M4|MCA3_MOUSE | Eukaryotic translation elongation factor 1 epsilon-1 OS=Mus musculus GN=Eef1e1 PE=2 SV=1 | 7 | 1.020 | 0.000 | 2.214 |
| sp|P55292|DSC2_MOUSE | Desmocollin-2 OS=Mus musculus GN=Dsc2 PE=2 SV=1 | 7 | 0.395 | 0.000 | InD |
| sp|Q80VP1|EPN1_MOUSE | Epsin-1 OS=Mus musculus GN=Epn1 PE=1 SV=3 | 7 | 1.203 | 0.000 | InD |
| sp|Q91W69|EPN3_MOUSE | Epsin-3 OS=Mus musculus GN=Epn3 PE=2 SV=1 | 7 | 1.203 | 0.000 | InD |
| sp|Q8K363|DDX18_MOUSE | ATP-dependent RNA helicase DDX18 OS=Mus musculus GN=Ddx18 PE=2 SV=1 | 7 | 1.213 | 0.000 | InD |
| sp|Q9CQZ5|NDUA6_MOUSE | NADH dehydrogenase [ubiquinone] 1 alpha subcomplex subunit 6 OS=Mus musculus GN=Ndufa6 PE=1 SV=1 | 7 | 1.216 | 0.000 | InD |
| sp|Q8BLN5|ERG7_MOUSE | Lanosterol synthase OS=Mus musculus GN=Lss PE=2 SV=2 | 7 | 1.327 | 0.000 | InD |
| sp|P63024|VAMP3_MOUSE | Vesicle-associated membrane protein 3 OS=Mus musculus GN=Vamp3 PE=1 SV=1 | 7 | 0.000 | 0.734 | InD |
| sp|Q9CQ40|RM49_MOUSE | 39S ribosomal protein L49, mitochondrial OS=Mus musculus GN=Mrpl49 PE=2 SV=1 | 7 | 1.206 | 0.857 | InD |
| sp|Q9JLQ0|CD2AP_MOUSE | CD2-associated protein OS=Mus musculus GN=Cd2ap PE=1 SV=3 | 7 | 0.949 | 0.868 | InD |
| sp|P58321|UCHL4_MOUSE | Ubiquitin carboxyl-terminal hydrolase isozyme L4 OS=Mus musculus GN=Uchl4 PE=1 SV=1 | 7 | 0.000 | 0.894 | InD |
| sp|P54726|RD23A_MOUSE | UV excision repair protein RAD23 homolog A OS=Mus musculus GN=Rad23a PE=1 SV=2 | 7 | 0.976 | 0.902 | InD |
| sp|Q9Z2Y8|PROSC_MOUSE | Proline synthase co-transcribed bacterial homolog protein OS=Mus musculus GN=Prosc PE=1 SV=1 | 7 | 1.145 | 0.910 | InD |
| sp|Q8BJL1|FBX30_MOUSE | F-box only protein 30 OS=Mus musculus GN=Fbxo30 PE=2 SV=2 | 7 | 0.954 | 0.941 | InD |
| sp|Q9JLJ5|ELOV1_MOUSE | Elongation of very long chain fatty acids protein 1 OS=Mus musculus GN=Elovl1 PE=2 SV=1 | 7 | InD | 0.946 | InD |
| sp|Q6TEK5|VKORL_MOUSE | Vitamin K epoxide reductase complex subunit 1-like protein 1 OS=Mus musculus GN=Vkorc1l1 PE=2 SV=1 | 7 | InD | 0.963 | InD |
| sp|Q69ZR2|HECD1_MOUSE | E3 ubiquitin-protein ligase HECTD1 OS=Mus musculus GN=Hectd1 PE=1 SV=2 | 7 | InD | 0.969 | InD |
| sp|Q8BXV2|BRI3B_MOUSE | BRI3-binding protein OS=Mus musculus GN=Bri3bp PE=2 SV=1 | 7 | 1.161 | 1.033 | InD |
| sp|Q9ERR7|SEP15_MOUSE | 15 kDa selenoprotein OS=Mus musculus GN=Sep15 PE=1 SV=3 | 7 | 0.935 | 1.063 | InD |
| sp|P28658|ATX10_MOUSE | Ataxin-10 OS=Mus musculus GN=Atxn10 PE=1 SV=2 | 7 | 1.437 | 1.089 | InD |
| sp|Q4KML4|ABRAL_MOUSE | Costars family protein ABRACL OS=Mus musculus GN=Abracl PE=2 SV=1 | 7 | 0.812 | 1.096 | InD |
| sp|P23492|PNPH_MOUSE | Purine nucleoside phosphorylase OS=Mus musculus GN=Pnp PE=1 SV=2 | 7 | 1.216 | 1.122 | InD |
| sp|Q9CQR2|RS21_MOUSE | 40S ribosomal protein S21 OS=Mus musculus GN=Rps21 PE=2 SV=1 | 7 | 1.271 | 1.157 | InD |
| sp|Q08943|SSRP1_MOUSE | FACT complex subunit SSRP1 OS=Mus musculus GN=Ssrp1 PE=1 SV=2 | 7 | 1.345 | 1.305 | InD |
| sp|Q9JII5|DAZP1_MOUSE | DAZ-associated protein 1 OS=Mus musculus GN=Dazap1 PE=2 SV=2 | 7 | 0.000 | 1.366 | InD |
| sp|Q9QY76|VAPB_MOUSE | Vesicle-associated membrane protein-associated protein B OS=Mus musculus GN=Vapb PE=2 SV=3 | 7 | 0.855 | InD | InD |
| sp|Q3TDN2|FAF2_MOUSE | FAS-associated factor 2 OS=Mus musculus GN=Faf2 PE=2 SV=2 | 7 | 0.965 | InD | InD |
| sp|P48024|EIF1_MOUSE | Eukaryotic translation initiation factor 1 OS=Mus musculus GN=Eif1 PE=2 SV=2 | 7 | 0.975 | InD | InD |
| sp|Q9CXU9|EIF1B_MOUSE | Eukaryotic translation initiation factor 1b OS=Mus musculus GN=Eif1b PE=2 SV=2 | 7 | 0.975 | InD | InD |
| sp|Q9JKV1|ADRM1_MOUSE | Proteasomal ubiquitin receptor ADRM1 OS=Mus musculus GN=Adrm1 PE=1 SV=2 | 7 | 1.020 | InD | InD |
| sp|Q8BZW8|NHLC2_MOUSE | NHL repeat-containing protein 2 OS=Mus musculus GN=Nhlrc2 PE=2 SV=1 | 7 | 1.037 | InD | InD |
| sp|O88696|CLPP_MOUSE | Putative ATP-dependent Clp protease proteolytic subunit, mitochondrial OS=Mus musculus GN=Clpp PE=2 SV=1 | 7 | 1.218 | InD | InD |
| sp|Q80UW5|MRCKG_MOUSE | Serine/threonine-protein kinase MRCK gamma OS=Mus musculus GN=Cdc42bpg PE=2 SV=2 | 7 | 1.369 | InD | InD |
| sp|Q05769|PGH2_MOUSE | Prostaglandin G/H synthase 2 OS=Mus musculus GN=Ptgs2 PE=1 SV=1 | 7 | 4.024 | InD | InD |
| sp|A2A8L5|PTPRF_MOUSE | Receptor-type tyrosine-protein phosphatase F OS=Mus musculus GN=Ptprf PE=1 SV=1 | 6 | 0.000 | 0.000 | 0.000 |
| sp|O88983|STX8_MOUSE | Syntaxin-8 OS=Mus musculus GN=Stx8 PE=1 SV=1 | 6 | 0.968 | 0.000 | 0.000 |
| sp|Q9R0Q6|ARC1A_MOUSE | Actin-related protein 2/3 complex subunit 1A OS=Mus musculus GN=Arpc1a PE=1 SV=1 | 6 | 1.021 | 0.000 | 0.000 |
| sp|Q8K0C9|GMDS_MOUSE | GDP-mannose 4,6 dehydratase OS=Mus musculus GN=Gmds PE=2 SV=1 | 6 | InD | 0.458 | 0.000 |
| sp|Q60766|IRGM1_MOUSE | Immunity-related GTPase family M protein 1 OS=Mus musculus GN=Irgm1 PE=1 SV=1 | 6 | 0.000 | 0.773 | 0.000 |
| sp|Q6ZQ73|CAND2_MOUSE | Cullin-associated NEDD8-dissociated protein 2 OS=Mus musculus GN=Cand2 PE=1 SV=2 | 6 | 0.960 | 0.812 | 0.000 |
| sp|Q62446|FKBP3_MOUSE | Peptidyl-prolyl cis-trans isomerase FKBP3 OS=Mus musculus GN=Fkbp3 PE=1 SV=2 | 6 | 1.280 | 0.866 | 0.000 |
| sp|Q8BRT1|CLAP2_MOUSE | CLIP-associating protein 2 OS=Mus musculus GN=Clasp2 PE=1 SV=1 | 6 | InD | 0.867 | 0.000 |
| sp|P70122|SBDS_MOUSE | Ribosome maturation protein SBDS OS=Mus musculus GN=Sbds PE=1 SV=4 | 6 | InD | 0.873 | 0.000 |
| sp|Q6NVF9|CPSF6_MOUSE | Cleavage and polyadenylation specificity factor subunit 6 OS=Mus musculus GN=Cpsf6 PE=1 SV=1 | 6 | 0.000 | 0.892 | 0.000 |
| sp|Q9WU28|PFD5_MOUSE | Prefoldin subunit 5 OS=Mus musculus GN=Pfdn5 PE=1 SV=1 | 6 | InD | 0.910 | 0.000 |
| sp|Q9DBQ7|PACE1_MOUSE | Protein-associating with the carboxyl-terminal domain of ezrin OS=Mus musculus GN=Scyl3 PE=1 SV=3 | 6 | InD | 1.150 | 0.000 |
| sp|Q8C0D5|ETUD1_MOUSE | Elongation factor Tu GTP-binding domain-containing protein 1 OS=Mus musculus GN=Eftud1 PE=2 SV=1 | 6 | InD | 1.380 | 0.000 |
| sp|Q8K4F5|ABHDB_MOUSE | Alpha/beta hydrolase domain-containing protein 11 OS=Mus musculus GN=Abhd11 PE=2 SV=1 | 6 | 1.104 | 1.543 | 0.000 |
| sp|Q9JI13|SAS10_MOUSE | Something about silencing protein 10 OS=Mus musculus GN=Utp3 PE=1 SV=1 | 6 | InD | 1.610 | 0.000 |
| sp|P29351|PTN6_MOUSE | Tyrosine-protein phosphatase non-receptor type 6 OS=Mus musculus GN=Ptpn6 PE=1 SV=2 | 6 | 0.000 | InD | 0.000 |
| sp|Q8JZR0|ACSL5_MOUSE | Long-chain-fatty-acid--CoA ligase 5 OS=Mus musculus GN=Acsl5 PE=2 SV=1 | 6 | 0.000 | InD | 0.000 |
| sp|Q9EPC1|PARVA_MOUSE | Alpha-parvin OS=Mus musculus GN=Parva PE=1 SV=1 | 6 | 0.000 | InD | 0.000 |
| sp|G5E8K5|ANK3_MOUSE | Ankyrin-3 OS=Mus musculus GN=Ank3 PE=1 SV=1 | 6 | 0.579 | InD | 0.000 |
| sp|Q9JIG7|CCD22_MOUSE | Coiled-coil domain-containing protein 22 OS=Mus musculus GN=Ccdc22 PE=1 SV=1 | 6 | 0.865 | InD | 0.000 |
| sp|P97390|VPS45_MOUSE | Vacuolar protein sorting-associated protein 45 OS=Mus musculus GN=Vps45 PE=1 SV=1 | 6 | 0.885 | InD | 0.000 |
| sp|Q63850|NUP62_MOUSE | Nuclear pore glycoprotein p62 OS=Mus musculus GN=Nup62 PE=1 SV=2 | 6 | 0.958 | InD | 0.000 |
| sp|Q0P678|ZCH18_MOUSE | Zinc finger CCCH domain-containing protein 18 OS=Mus musculus GN=Zc3h18 PE=1 SV=1 | 6 | 0.970 | InD | 0.000 |
| sp|Q8K1R3|PNPT1_MOUSE | Polyribonucleotide nucleotidyltransferase 1, mitochondrial OS=Mus musculus GN=Pnpt1 PE=1 SV=1 | 6 | 1.034 | InD | 0.000 |
| sp|Q91YR7|PRP6_MOUSE | Pre-mRNA-processing factor 6 OS=Mus musculus GN=Prpf6 PE=2 SV=1 | 6 | 1.133 | InD | 0.000 |
| sp|Q8BYL4|SYYM_MOUSE | Tyrosine--tRNA ligase, mitochondrial OS=Mus musculus GN=Yars2 PE=2 SV=2 | 6 | 1.217 | InD | 0.000 |
| sp|Q6A0A2|LAR4B_MOUSE | La-related protein 4B OS=Mus musculus GN=Larp4b PE=1 SV=2 | 6 | 1.241 | InD | 0.000 |
| sp|Q8K0D5|EFGM_MOUSE | Elongation factor G, mitochondrial OS=Mus musculus GN=Gfm1 PE=2 SV=1 | 6 | 1.271 | InD | 0.000 |
| sp|Q7TNP2|2AAB_MOUSE | Serine/threonine-protein phosphatase 2A 65 kDa regulatory subunit A beta isoform OS=Mus musculus GN=Ppp2r1b PE=1 SV=2 | 6 | InD | InD | 0.000 |
| sp|Q99LI8|HGS_MOUSE | Hepatocyte growth factor-regulated tyrosine kinase substrate OS=Mus musculus GN=Hgs PE=1 SV=2 | 6 | InD | InD | 0.000 |
| sp|Q3U1J4|DDB1_MOUSE | DNA damage-binding protein 1 OS=Mus musculus GN=Ddb1 PE=1 SV=2 | 6 | 0.000 | 1.229 | 0.410 |
| sp|P21278|GNA11_MOUSE | Guanine nucleotide-binding protein subunit alpha-11 OS=Mus musculus GN=Gna11 PE=1 SV=1 | 6 | InD | InD | 0.453 |
| sp|Q9CQF0|RM11_MOUSE | 39S ribosomal protein L11, mitochondrial OS=Mus musculus GN=Mrpl11 PE=2 SV=1 | 6 | InD | 1.000 | 0.578 |
| sp|P26450|P85A_MOUSE | Phosphatidylinositol 3-kinase regulatory subunit alpha OS=Mus musculus GN=Pik3r1 PE=1 SV=2 | 6 | 0.794 | InD | 0.635 |
| sp|Q9CXT8|MPPB_MOUSE | Mitochondrial-processing peptidase subunit beta OS=Mus musculus GN=Pmpcb PE=2 SV=1 | 6 | 1.667 | 0.000 | 0.680 |
| sp|O35382|EXOC4_MOUSE | Exocyst complex component 4 OS=Mus musculus GN=Exoc4 PE=1 SV=2 | 6 | 0.000 | InD | 0.719 |
| sp|Q8BZS9|DHX32_MOUSE | Putative pre-mRNA-splicing factor ATP-dependent RNA helicase DHX32 OS=Mus musculus GN=Dhx32 PE=2 SV=2 | 6 | InD | 1.198 | 0.777 |
| sp|P97346|NXN_MOUSE | Nucleoredoxin OS=Mus musculus GN=Nxn PE=1 SV=1 | 6 | 1.140 | InD | 0.832 |
| sp|Q9ERF3|WDR61_MOUSE | WD repeat-containing protein 61 OS=Mus musculus GN=Wdr61 PE=2 SV=1 | 6 | 1.132 | InD | 0.836 |
| sp|P46460|NSF_MOUSE | Vesicle-fusing ATPase OS=Mus musculus GN=Nsf PE=1 SV=2 | 6 | InD | InD | 0.873 |
| sp|Q64464|CP3AD_MOUSE | Cytochrome P450 3A13 OS=Mus musculus GN=Cyp3a13 PE=2 SV=1 | 6 | 0.000 | InD | 0.907 |
| sp|Q8BFZ9|ERLN2_MOUSE | Erlin-2 OS=Mus musculus GN=Erlin2 PE=1 SV=1 | 6 | InD | 0.779 | 0.923 |
| sp|Q32M21|GSDA2_MOUSE | Gasdermin-A2 OS=Mus musculus GN=Gsdma2 PE=2 SV=1 | 6 | InD | 0.894 | 0.934 |
| sp|Q5Y4Y6|GSDA3_MOUSE | Gasdermin-A3 OS=Mus musculus GN=Gsdma3 PE=1 SV=1 | 6 | InD | 0.894 | 0.934 |
| sp|Q6P9R2|OXSR1_MOUSE | Serine/threonine-protein kinase OSR1 OS=Mus musculus GN=Oxsr1 PE=1 SV=1 | 6 | InD | InD | 0.951 |
| sp|Q80X73|PELO_MOUSE | Protein pelota homolog OS=Mus musculus GN=Pelo PE=2 SV=3 | 6 | InD | InD | 0.989 |
| sp|P05132|KAPCA_MOUSE | cAMP-dependent protein kinase catalytic subunit alpha OS=Mus musculus GN=Prkaca PE=1 SV=3 | 6 | InD | 0.887 | 0.994 |
| sp|Q7TSQ8|PDPR_MOUSE | Pyruvate dehydrogenase phosphatase regulatory subunit, mitochondrial OS=Mus musculus GN=Pdpr PE=2 SV=1 | 6 | 0.000 | InD | 1.003 |
| sp|P55264|ADK_MOUSE | Adenosine kinase OS=Mus musculus GN=Adk PE=1 SV=2 | 6 | 1.947 | InD | 1.005 |
| sp|P97379|G3BP2_MOUSE | Ras GTPase-activating protein-binding protein 2 OS=Mus musculus GN=G3bp2 PE=1 SV=2 | 6 | InD | InD | 1.110 |
| sp|Q8K021|SCAM1_MOUSE | Secretory carrier-associated membrane protein 1 OS=Mus musculus GN=Scamp1 PE=1 SV=1 | 6 | 0.000 | InD | 1.151 |
| sp|Q99LI7|CSTF3_MOUSE | Cleavage stimulation factor subunit 3 OS=Mus musculus GN=Cstf3 PE=1 SV=1 | 6 | 0.865 | InD | 1.152 |
| sp|O88967|YMEL1_MOUSE | ATP-dependent zinc metalloprotease YME1L1 OS=Mus musculus GN=Yme1l1 PE=2 SV=1 | 6 | 0.788 | InD | 1.176 |
| sp|P97310|MCM2_MOUSE | DNA replication licensing factor MCM2 OS=Mus musculus GN=Mcm2 PE=1 SV=3 | 6 | 0.000 | InD | 1.486 |
| sp|Q8CIG8|ANM5_MOUSE | Protein arginine N-methyltransferase 5 OS=Mus musculus GN=Prmt5 PE=1 SV=3 | 6 | InD | 0.000 | 1.548 |
| sp|Q91ZA3|PCCA_MOUSE | Propionyl-CoA carboxylase alpha chain, mitochondrial OS=Mus musculus GN=Pcca PE=2 SV=2 | 6 | 0.000 | 0.000 | InD |
| sp|Q9QUR7|PIN1_MOUSE | Peptidyl-prolyl cis-trans isomerase NIMA-interacting 1 OS=Mus musculus GN=Pin1 PE=1 SV=1 | 6 | 0.866 | 0.000 | InD |
| sp|Q9CXS4|CENPV_MOUSE | Centromere protein V OS=Mus musculus GN=Cenpv PE=2 SV=2 | 6 | 0.930 | 0.000 | InD |
| sp|Q80UY1|CI041_MOUSE | UPF0586 protein C9orf41 homolog OS=Mus musculus PE=2 SV=1 | 6 | 1.175 | 0.000 | InD |
| sp|P11531|DMD_MOUSE | Dystrophin OS=Mus musculus GN=Dmd PE=1 SV=3 | 6 | 0.000 | 0.394 | InD |
| sp|Q9Z1Z2|STRAP_MOUSE | Serine-threonine kinase receptor-associated protein OS=Mus musculus GN=Strap PE=1 SV=2 | 6 | 1.108 | 0.810 | InD |
| sp|Q80X95|RRAGA_MOUSE | Ras-related GTP-binding protein A OS=Mus musculus GN=Rraga PE=2 SV=1 | 6 | InD | 0.883 | InD |
| sp|Q922B1|MACD1_MOUSE | O-acetyl-ADP-ribose deacetylase MACROD1 OS=Mus musculus GN=Macrod1 PE=2 SV=2 | 6 | 1.049 | 0.989 | InD |
| sp|P62627|DLRB1_MOUSE | Dynein light chain roadblock-type 1 OS=Mus musculus GN=Dynlrb1 PE=1 SV=3 | 6 | 1.046 | 1.014 | InD |
| sp|Q9CWL8|CTBL1_MOUSE | Beta-catenin-like protein 1 OS=Mus musculus GN=Ctnnbl1 PE=1 SV=1 | 6 | 0.000 | 1.022 | InD |
| sp|Q5XG71|UTP20_MOUSE | Small subunit processome component 20 homolog OS=Mus musculus GN=Utp20 PE=2 SV=2 | 6 | 0.944 | 1.077 | InD |
| sp|Q6PGL7|FAM21_MOUSE | WASH complex subunit FAM21 OS=Mus musculus GN=Fam21 PE=1 SV=1 | 6 | InD | 1.079 | InD |
| sp|Q9EQW7|KI13A_MOUSE | Kinesin-like protein KIF13A OS=Mus musculus GN=Kif13a PE=1 SV=1 | 6 | 0.000 | 1.108 | InD |
| sp|Q7TT37|ELP1_MOUSE | Elongator complex protein 1 OS=Mus musculus GN=Ikbkap PE=2 SV=2 | 6 | 0.949 | 1.174 | InD |
| sp|Q8BR90|CE051_MOUSE | UPF0600 protein C5orf51 homolog OS=Mus musculus PE=2 SV=1 | 6 | 0.943 | 1.287 | InD |
| sp|O35682|MYADM_MOUSE | Myeloid-associated differentiation marker OS=Mus musculus GN=Myadm PE=2 SV=2 | 6 | 0.212 | 4.143 | InD |
| sp|O88990|ACTN3_MOUSE | Alpha-actinin-3 OS=Mus musculus GN=Actn3 PE=2 SV=1 | 6 | 0.000 | InD | InD |
| sp|Q9CXZ1|NDUS4_MOUSE | NADH dehydrogenase [ubiquinone] iron-sulfur protein 4, mitochondrial OS=Mus musculus GN=Ndufs4 PE=1 SV=3 | 6 | 0.859 | InD | InD |
| sp|Q8BHH2|RAB9B_MOUSE | Ras-related protein Rab-9B OS=Mus musculus GN=Rab9b PE=2 SV=1 | 6 | 0.975 | InD | InD |
| sp|P08775|RPB1_MOUSE | DNA-directed RNA polymerase II subunit RPB1 OS=Mus musculus GN=Polr2a PE=1 SV=3 | 6 | 1.091 | InD | InD |
| sp|P62254|UB2G1_MOUSE | Ubiquitin-conjugating enzyme E2 G1 OS=Mus musculus GN=Ube2g1 PE=2 SV=3 | 6 | 1.157 | InD | InD |
| sp|P43247|MSH2_MOUSE | DNA mismatch repair protein Msh2 OS=Mus musculus GN=Msh2 PE=2 SV=1 | 6 | 1.175 | InD | InD |
| sp|Q91VK1|BZW2_MOUSE | Basic leucine zipper and W2 domain-containing protein 2 OS=Mus musculus GN=Bzw2 PE=1 SV=1 | 6 | 1.188 | InD | InD |
| sp|Q8VHE0|SEC63_MOUSE | Translocation protein SEC63 homolog OS=Mus musculus GN=Sec63 PE=1 SV=4 | 6 | 1.231 | InD | InD |
| sp|Q61699|HS105_MOUSE | Heat shock protein 105 kDa OS=Mus musculus GN=Hsph1 PE=1 SV=2 | 6 | 1.246 | InD | InD |
| sp|P61294|RAB6B_MOUSE | Ras-related protein Rab-6B OS=Mus musculus GN=Rab6b PE=1 SV=1 | 6 | 1.297 | InD | InD |
| sp|Q9D0B6|CX026_MOUSE | UPF0368 protein Cxorf26 homolog OS=Mus musculus PE=2 SV=1 | 6 | 1.329 | InD | InD |
| sp|Q9CQ54|NDUC2_MOUSE | NADH dehydrogenase [ubiquinone] 1 subunit C2 OS=Mus musculus GN=Ndufc2 PE=1 SV=1 | 6 | 1.436 | InD | InD |
| sp|Q9JMA9|S6A14_MOUSE | Sodium- and chloride-dependent neutral and basic amino acid transporter B(0+) OS=Mus musculus GN=Slc6a14 PE=2 SV=1 | 6 | 1.980 | InD | InD |
| sp|P10648|GSTA2_MOUSE | Glutathione S-transferase A2 OS=Mus musculus GN=Gsta2 PE=1 SV=3 | 6 | InD | InD | InD |
| sp|Q8BT60|CPNE3_MOUSE | Copine-3 OS=Mus musculus GN=Cpne3 PE=1 SV=2 | 6 | InD | InD | InD |

**Supplementary table LEGEND**

Total PC: Total peptide count; L/C: Lysate/Control;

InD: Indeterminate (means that the control was 0, i.e. when divided by control, the value is indeterminate).
